# The past, present, and future of coral reef growth in the Florida Keys

**DOI:** 10.1111/gcb.16295

**Published:** 2022-07-05

**Authors:** Lauren T. Toth, Travis A. Courtney, Michael A. Colella, Selena A. Kupfner Johnson, Robert R. Ruzicka

**Affiliations:** ^1^ U.S. Geological Survey St. Petersburg Coastal and Marine Science Center St. Petersburg Florida USA; ^2^ Scripps Institution of Oceanography University of California San Diego La Jolla California USA; ^3^ Department of Marine Sciences University of Puerto Rico Mayagüez Mayagüez Puerto Rico; ^4^ Fish & Wildlife Research Institute, Florida Fish & Wildlife Conservation Commission St. Petersburg Florida USA

**Keywords:** bioerosion, carbonate budgets, Florida Keys, reef accretion, restoration, thermal stress

## Abstract

Coral‐reef degradation is driving global‐scale reductions in reef‐building capacity and the ecological, geological, and socioeconomic functions it supports. The persistence of those essential functions will depend on whether coral‐reef management is able to rebalance the competing processes of reef accretion and erosion. Here, we reconstructed census‐based carbonate budgets of 46 reefs throughout the Florida Keys from 1996 to 2019. We evaluated the environmental and ecological drivers of changing budget states and compared historical trends in reef‐accretion potential to millennial‐scale baselines of accretion from reef cores and future projections with coral restoration. We found that historically, most reefs had positive carbonate budgets, and many had reef‐accretion potential comparable to the ~3 mm year^−1^ average accretion rate during the peak of regional reef building ~7000 years ago; however, declines in reef‐building *Acropora palmata* and *Orbicella* spp. corals following a series of thermal stress events and coral disease outbreaks resulted in a shift from positive to negative budgets for most reefs in the region. By 2019, only ~15% of reefs had positive net carbonate production. Most of those reefs were in inshore, Lower Keys patch‐reef habitats with low water clarity, supporting the hypothesis that environments with naturally low irradiance may provide a refugia from thermal stress. We caution that our estimated carbonate budgets are likely overly optimistic; comparison of reef‐accretion potential to measured accretion from reef cores suggests that, by not accounting for the role of nonbiological physical and chemical erosion, census‐based carbonate budgets may underestimate total erosion by ~1 mm year^−1^ (−1.15 kg CaCO_3_ m^−2^ year^−1^). Although the present state of Florida's reefs is dire, we demonstrate that the restoration of reef‐building corals has the potential to help mitigate declines in reef accretion in some locations, which could allow some key ecosystem functions to be maintained until the threat of global climate change is addressed.

## Graphical abstract

We used carbonate budgets to reconstruct reef‐accretion potential at 46 reefs throughout the Florida Keys from 1996–2019. Whereas in 1996 many reefs grew at rates comparable with millennial‐scale baselines from reef cores, over the next two decades, thermal stress drove substantial losses of reef‐building corals. As a result, erosion is now the dominant process on most reefs in the region. Although the present state of Florida’s reefs appears dire, we demonstrate that coral restoration has the potential to reverse long‐term declines in reef growth on a local scale and preserve key reef functions until global‐scale threats are mitigated.

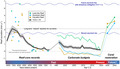

## 1 INTRODUCTION

The complex, three‐dimensional reef frameworks built by corals over hundreds to thousands of years serve as the foundation for the invaluable ecosystem services coral reefs provide to society (Kuffner & Toth, 2016; Perry & Alvarez‐Filip, 2019). Reefs create essential coastal habitats that are hotspots for biodiversity, support fisheries that offer revenue and food security, and buoy local economies through tourism (reviewed in Woodhead et al., 2019). Reef frameworks also act as natural barriers that dissipate wave energy and protect natural and human communities on reef‐lined coasts from flooding and erosion (Beck et al., 2018; Reguero et al., 2021). The persistence of these essential ecosystem services depends on the continued growth and maintenance of reef framework: a function that is increasingly threatened by coral‐reef degradation (Perry & Alvarez‐Filip, 2019; Pratchett et al., 2014; Woodhead et al., 2019).

For more than 50 years, climate change and other anthropogenic disturbances have driven declines in coral populations around the world (Bruno et al., 2019; Hughes et al., 2018), and the impacts on reef‐building species have been especially severe (Burman et al., 2012; Kuffner & Toth, 2016; Perry et al., 2015; Toth et al., 2019). These changes have caused global‐scale reductions in carbonate production and the capacity for continued reef accretion (Alvarez‐Filip et al., 2013; Courtney et al., 2020; Kennedy et al., 2013; Perry et al., 2013, 2015, 2018; Perry & Alvarez‐Filip, 2019). Rates of biological, physical, and chemical erosion are increasingly outpacing rates of carbonate production (Eyre et al., 2018; Perry et al., 2013, 2014, 2018). As a result, many reef frameworks are becoming less structurally complex (Alvarez‐Filip et al., 2013; Roff et al., 2020) and some are rapidly losing elevation (Kuffner et al., 2019; Yates et al., 2017). Without significant intervention, climate change is predicted to accelerate shifts toward net erosive states for more and more reefs around the world (Cornwall et al., 2021; Eyre et al., 2018; Kennedy et al., 2013).

The increasing role of erosion on coral reefs has the potential to reshape their geological, ecological, and socioeconomic functions (Perry & Alvarez‐Filip, 2019; Woodhead et al., 2019). For example, Beck et al. (2018) estimated that the loss of 1 m of reef elevation (cf. Yates et al., 2017) and associated declines in structural complexity (i.e., reef flattening) would more than double the impact of flooding and storms on reef‐lined coasts globally by the end of the century. The capacity of reefs to provide this coastal protection function will be further reduced by sea‐level rise (Beck et al., 2018; Reguero et al., 2021). Reef‐framework degradation will also reduce habitat for fish and other reef‐associated biota (Pratchett et al., 2014; Roff et al., 2020). These impending consequences highlight the importance of considering the balance between reef accretion and erosion to design effective coral‐reef management and restoration strategies (Kuffner & Toth, 2016).

Census‐based carbonate budget studies, which are aimed at quantifying the biological processes that regulate reef‐framework construction and destruction, have a long history in coral‐reef research (e.g., Chave et al., 1972; Eakin, 1996; Hubbard et al., 1990; Lange et al., 2020; Perry et al., 2012). Although this approach generally does not account for the impacts of event‐driven physical erosion or chemical dissolution, it provides a valuable tool for using existing coral‐reef monitoring data to assess the present state and reconstruct temporal changes in the balance between carbonate production and bioerosion (e.g., Estrada‐Saldívar et al., 2019; Januchowski‐Hartley et al., 2017; Molina‐Hernández et al., 2020; Perry et al., 2013, 2018). These methods can also be used to identify the environmental (de Bakker et al., 2019; Eakin, 1996; Lange & Perry, 2019) and ecological drivers of changing budget states (Courtney et al., 2020; Januchowski‐Hartley et al., 2017; Molina‐Hernández et al., 2020; Perry et al., 2014, 2015) and to predict how reefs may respond to climate change and other anthropogenic disturbances in the future (Cornwall et al., 2021; Kennedy et al., 2013). There is a critical need, however, to better quantify how carbonate budgets vary over space and time (Lange et al., 2020).

Although the reefs of south Florida are arguably among the best‐studied coral‐reef ecosystems in the world, rates of carbonate production and/or bioerosion have only been estimated for a handful of sites in the region (Cornwall et al., 2021; Courtney et al., 2020; Enochs et al., 2015; Kuffner et al., 2019; Manzello et al., 2018; Perry et al., 2018). Florida's reefs have suffered significant ecological degradation in recent decades (Burman et al., 2012; Ruzicka et al., 2013; Toth et al., 2014), and the geological process of reef building in this environmentally marginal, subtropical setting has been limited for at least 3000 years (Toth et al., 2019, 2021; Toth, Kuffner, Stathakopoulos, & Shinn, 2018). Despite the generally degraded status of Florida's reefs, regional environmental variability has produced considerable heterogeneity in ecological states and processes (Guest et al., 2018; Kuffner et al., 2013, 2020; Lenz et al., 2021), suggesting that carbonate budgets may likewise vary (Courtney et al., 2020; Manzello et al., 2018).

Here, we provide a broad perspective on the millennial‐ to decadal‐scale history, present state, and future prospects of the accretion–erosion balance in the Florida Keys. We reconstructed changes in carbonate production to estimate reef‐accretion potential at 46 reefs throughout the region from 1996 to 2019 and compared it with changes in reef accretion during the last 8500 years and with projected changes under future restoration scenarios. We show that declines in reef‐building corals over the last two decades have resulted in a regionwide decline in carbonate production such that erosion is now the dominant process on most reefs throughout the Florida Keys. This suggests that without active restoration of reef‐building corals, the persistence of key reef functions in this region may be in jeopardy.

## 2 METHODS

### 2.1 Regional setting

**FIGURE 1:**
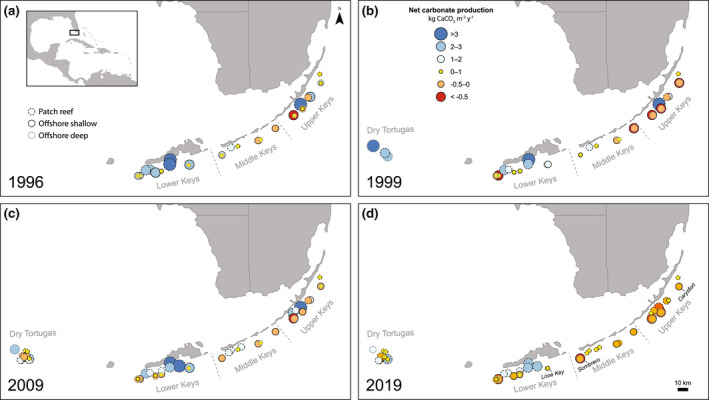
Changes in net carbonate production (kg CaCO_3_ m^−2^ year^−1^) from 1996 to 2019 at the 46 study sites throughout the Florida Keys reef tract. The outline of the points is formatted to differentiate the three habitat types: patch (dashed lines), offshore shallow (solid black lines), and offshore deep (solid gray lines) reefs. The three sites that are included in the Mission: Iconic Reefs restoration initiative (NOAA, 2021) are labeled in 2019 (panel d). Note that new sites were added over time (see Supplementary Methods). Map image is the intellectual property of Esri and is used herein under license. Copyright 2020 Esri and its licensors. All rights reserved.

The Florida Keys reef tract (FKRT) extends ~350 km along the islands of the Florida Keys from southern Miami to Dry Tortugas National Park (Figure 1). The Upper, Middle, and Lower Keys subregions are deliniated based on their distinct geologic histories and their proximity to the influence of inimical waters from Florida Bay (Toth, Kuffner, & Stathakopoulos, 2018). Within the Keys subregions, there are two primary reef habitats: shelf‐edge reefs, located 5–7 km offshore, and inshore patch reefs (Ruzicka et al., 2009). Dry Tortugas National Park is composed of bank‐barrier reefs, patch reefs, and pinnacle habitats (Ruzicka et al., 2009) and is located in an open‐ocean environment, more than 100 km from the inhabited islands of the Florida Keys.

### 2.2 Calculating carbonate budgets

We estimated the carbonate budgets of 46 sites across the FKRT that were surveyed annually by the Florida Fish and Wildlife Research Institute's Coral Reef Evaluation and Monitoring Program (CREMP) between 1996 and 2019 (Table S1; Ruzicka et al., 2013, 2009). The CREMP sites represent four subregions of the FKRT—the Upper, Middle, and Lower Keys, and the Dry Tortugas—and three habitat types—inshore patch reefs (1.8–12.8 m), offshore shallow reefs (1.8–7.3 m), and offshore deep reefs (10.7–16.5 m). At each site, CREMP collects benthic imagery along 2–4, ~22 m transects and quantifies the coverage of coral taxa and other benthos based on point counts (see Supplementary Methods for details on the CREMP surveys).

We combined data on the benthic coverage of hard corals and coralline algae from CREMP with the taxon‐specific, area‐normalized calcification rates provided in Courtney et al. (2021) to calculate annual mean (± standard error [SE]) gross carbonate production (kg CaCO_3_ m^−2^ year^−1^) using an adaptation of the ReefBudget v2 methodology (Perry & Lange, 2019; see Table S2 and Supplementary Methods in Appendix S1 for details). We also derived data on the abundance of the bioeroding urchin *Diadema antillarum*, surface area occupied by the bioeroding sponges *Cliothosa delitrix*, *Cliona caribbaea*, *Cliona varians*, and *Pione lampa* (macro‐endolithic bioerosion), and available (consolidated) substrate for microbioerosion from CREMP. Those census data were converted into estimates of mean (±SE) bioerosion (kg CaCO_3_ m^−2^ year^−1^) at each site following the ReefBudget v2 methodology (Perry & Lange, 2019) with a few necessary modifications (Table S2). Those modifications include: (1) using an empirical distribution of *D. antillarum* test sizes for the Florida Keys based on data collected in 2007 (Chiappone et al., 2008) to calculate bioerosion rates because test sizes of urchins were not measured by CREMP, (2) omitting bioerosion by the urchins *Echinometra lucunter*, *Echinometra viridis*, and *Eucidaris tribuloides* and the sponge *Siphonodictyon* spp., as these taxa were not recorded in the CREMP surveys, and (3) assuming sponge and microbioerosion did not vary significantly over time (i.e., using single site level means for those variables), as the necessary census data were not available for all years of our study (see Supplementary Methods).

CREMP does not monitor fish, so data on parrotfish bioerosion were instead derived from the Reef Visual Census (RVC) program (Brandt et al., 2009), now a part of the National Oceanic and Atmospheric Administration's (NOAA's) National Coral Reef Monitoring Program (NCRMP), which has conducted habitat‐ and depth‐stratified, random stationary fish surveys at hundreds of sites through the region annually (RVC: 1999–2012) or biannually (NCREMP‐RVC: 2014–2018) since 1999. We extracted size‐specific counts of the bioeroding parrotfish species *Sparisoma viride*, *Sp. aurofrenatum*, *Sp. rubripinne*, *Sp. chrysopterum*, *Scarus vetula*, *Sc. taeniopterus*, *Sc. iseri*, *Sc. guacamaia*, and *Sc. coelestinus* from all available years in the RVC database (https://grunt.sefsc.noaa.gov/rvc_analysis20/). We used data from RVC sites in similar reef habitats and depths within a 10‐km radius of each CREMP site to approximate 8‐year running means of parrotfish abundances at our sites. We converted those parrotfish size–frequency data into annual estimates of mean (±SE) parrotfish bioerosion based on the ReefBudget v2 methodology (Perry & Lange, 2019; see Supplementary Methods). Although our methodology allowed us to approximate the ReefBudget v2 estimates of bioerosion (Perry & Lange, 2019), the necessary spatial and temporal averaging to fill in data gaps limits our capacity to detect any major changes in bioerosion over the time series of this study (Table S2).

The gross carbonate production and bioerosion data were combined to estimate annual mean (±SE) net carbonate production (kg CaCO_3_ m^−2^ year^−1^) by reef biota at each site. We also converted the net carbonate production data into estimates of reef‐accretion potential (mm year^−1^) using the regional mean reef‐framework porosity for the Florida Keys of 0.63 (±0.02 SE; Toth, Kuffner, & Stathakopoulos, 2018; Toth, Kuffner, Stathakopoulos, & Shinn, 2018; see Supplementary Methods). We follow Perry et al. (2018) in considering the rates of reef accretion determined from the carbonate budgets to represent the estimates of reef‐accretion *potential*, which are likely overestimates of true reef accretion because they do not incorporate erosion by physical processes or chemical dissolution, and because Holocene framework porosity may overestimate framework volumes constructed by today's smaller, less rugose coral assemblages. To provide context for recent changes, reef‐accretion potential was compared with regional, Holocene accretion rates determined from radiometric dating of reef cores collected throughout the FKRT. These geological records of reef accretion are described in detail by Toth et al. (Toth, Kuffner, & Stathakopoulos, 2018; Toth, Kuffner, Stathakopoulos, & Shinn, 2018).

Finally, we used our carbonate budgets to evaluate the potential impacts of restoration at three sites included in both our study and the NOAA's Mission: Iconic Reefs (M:IR) restoration initiative (NOAA, 2021): Looe Key, Sombrero, and Carysfort reefs (Figure 1d). M:IR is an ambitious, multiagency effort aimed a scaling up coral restoration at seven “iconic” reefs on the FKRT, with the ultimate goal of not only restoring coral populations to historic baselines (i.e., before declines beginning in the 1980s) but also restoring reef function. Phase 1 of M:IR aims to increase average coral cover on the reefs to 15% over the first 10 years (by 2030 C.E.) and will focus on outplanting of *A. palmata* and *A. cervicornis* with a smaller contribution of massive taxa like *Orbicella* spp. During Phase 2, outplanting efforts will continue, with a focus on increasing coral diversity and reaching the 20‐year target of 25% coral cover by 2040 C.E. The proposed restoration plan under M:IR explicitly incorporates empirical estimates of coral‐colony growth and outplant mortality rates and has built‐in redundancy based on the assumption that stochastic mortality events will continue to occur in the future (NOAA, 2021).

We used the phased, species‐specific M:IR coral‐cover targets for *A. palmata*, *A. cervicornis*, and *Orbicella* spp. (provided in Table S3) for habitats analogous to CREMP's shallow survey locations at Looe Key, Sombrero, and Carysfort reefs to evaluate how reef‐accretion potential at those sites could change by 2030 and 2040 C.E. if the restoration plan is successful. We note that whereas other coral taxa are included in the M:IR plan, they were not included in our analysis because either the species‐level coral cover targets have not yet been defined (for “brain corals” and “other corals”) or because the proposed increases in cover were negligible (i.e., only 0.25% for *Dendrogyra cylindrus*). To evaluate the relative impacts of the different coral taxa targeted for restoration, we also ran generalized restoration scenarios for *A. palmata* and *A. cervicornis* (+5, 10, and 15% cover) and *Orbicella* spp. (+1, 2, and 5% cover) that bound the M:IR coral cover targets for those taxa (Tables S3 and S4). For comparison, we applied the coral cover targets for *Orbicella* spp. to *S. siderea*, a stress‐tolerant species whose relative abundance has been increasing regionally in recent decades (Burman et al., 2012; Toth et al., 2014). For each scenario, the theoretical restored coverages of those species were substituted for their observed percent cover at each reef in 2019, while holding the production and erosion rates of all other taxa constant. We then recalculated the carbonate budgets at each site to quantify how reef‐accretion potential could change if the restored coral cover targets were met.

### 2.3 Data analysis

All carbonate budget calculations and statistical analyses were conducted in RStudio (R Core Team, 2021) and our code and all supporting datasets are available on GitLab (Toth & Courtney, 2022; https://doi.org/10.5066/P9APPZHJ). We analyzed spatial and temporal variability in mean net carbonate production, reef‐accretion potential, and bioerosion at the 46 sites using linear‐mixed effects models (LMEs; *nlme* package; Pinheiro, DebRoy, Sarkar, & Team, 2021) with year (1996–2019), subregion (Upper, Middle, and Lower Keys and the Dry Tortugas), and habitat (inshore patch, offshore shallow, and offshore deep reefs) as fixed effects and site as a random intercept. We also used LMEs to identify the thresholds of coral cover needed to maintain positive net carbonate production by running models with coral cover as a fixed effect. To identify the coral taxa responsible for observed changes in carbonate production, we used LMEs (fixed factors: year, subregion, and habitat; random intercept: site) to analyze trends in the percent cover and the percent contribution to gross carbonate production for the seven coral taxa that had mean site‐level carbonate production rates >0.05 kg CaCO_3_ m^−2^ year^−1^ in any year: *Orbicella* spp., *Montastraea cavernosa*, *Siderastrea siderea*, *Porites astreoides*, *Colpophyllia natans*, *Acropora palmata*, and *A. cervicornis*. We focus on the results for the three taxa*—Orbicella* spp., *A. palmata*, and *S. siderea*—that had significant changes in both coral cover and relative carbonate production over time and present the results for the other taxa in the Supplementary Results & Discussion.

Although spatial autocorrelation in our datasets was negligible (Mantel tests: *r* = −0.09 to 0.08 for all variables and *p* > .05 for most variables; Table S5), there was evidence of significant time lags (*ACF* function). We therefore used the *corARMA* function (Pinheiro et al., 2021) to incorporate temporal autocorrelation structures into the LMEs. For each model, we evaluated six moving‐average autocorrelation structures (p and q = 0–2) and report the results from the best‐fit model based on Akaike's information criterion (Toth & Courtney, 2022). We evaluated the fixed effects of the LMEs using the *anova* function. Post hoc comparisons of significant fixed effects were conducted using the Tukey method in *emmeans* (Lenth, 2021) and were used to identify significant differences among habitats and subregions. We also evaluated the Tukey pairwise comparisons for the beginning and end of the study (1996 vs. 2019) and those representing CREMP surveys before and after high and low thermal stress events (Colella et al., 2012; Manzello, 2015) and Category 1–5 hurricane impacts (all of which primarily impacted the Lower Keys and Dry Tortugas subregions; https://coast.noaa.gov/hurricanes/), which we predicted *a priori* could cause significant changes on the reefs. This resulted in seven pairwise temporal comparisons that were used to evaluate: (1) the beginning versus the end of the time series (1996 vs. 2019), (2) the 1997–1998 coral‐bleaching event and Hurricane Georges (Category 1) in September of 1998 (1996 vs. 1999), (3) Hurricane Irene (Category 1) in October of 1999 (1999 vs. 2000), (4) Hurricane Charley in August 2004 and Hurricanes Dennis, Katrina, Rita, and Wilma in July–October of 2005 (all Category 2) as well as a regional coral‐bleaching event in 2005 (2003 vs. 2006), (5) the January 2010 cold‐water event (2009 vs. 2010), (6) the regional coral‐bleaching events in 2014 and 2015 (2013 vs. 2016), and (7) Hurricane Irma (Category 4) in September 2017 (2017 vs. 2018). The other temporal pairwise comparisons are available on GitLab (Toth & Courtney, 2022).

**FIGURE 2:**
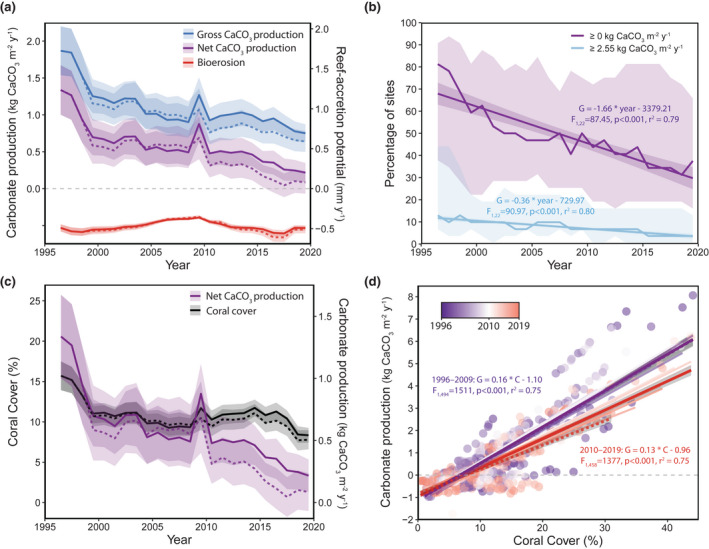
Regional changes in carbonate budgets from 1996 to 2019. (a) Changes in mean (lines) ± standard error (SE; shaded area) gross carbonate production (blue), bioerosion (red), net carbonate production (purple), and reef‐accretion potential (purple; secondary y‐axis) regionwide (site‐level budgets are plotted in Figures S2–S4). (b) Trends in the mean (solid lines) ± SE (shading) percentage of the 32 sites surveyed from 1996 to 2019 with net carbonate production (G) that was positive (purple) or at least as high as the western Atlantic mean of 2.55 kg m^−2^ year^−1^ (Perry et al., 2018) over time. (c) Changes in mean (lines) ± SE (shaded area) coral cover (black) and net carbonate production (purple). (d) Relationship between coral cover (C) and net carbonate production (G) from 1996 to 2009 (purple lines and points) and from 2010 to 2019 (red lines and points). Thin lines represent individual years and thick lines with shading (± SE) summarize how the relationship changed between those periods. In panels (a, c, and d), solid lines (and solid points in d) represent annual means for all 46 sites, whereas dashed lines are annual means for the 32 sites that were surveyed annually from 1996 to 2019. We note that the apparent increase in coral cover in panel (c) and, therefore, carbonate production (panels a and c) in 2009 is primarily an artifact of the addition of six new patch‐reef sites with relatively high coral cover in that year (see Supplementary Results & Discussion). Annual means ± SE were calculated using site‐level means.

Although those pairwise comparisons allowed us to evaluate the impact of pulse disturbance events, the timing of stony coral tissue loss disease (SCTLD) emergence varied across the reef tract (Muller et al., 2020); the effects of the disease first became apparent at our sites in the Upper Keys in 2017 but did not reach the Lower Keys sites until 2019. We therefore compared net carbonate budgets and cover of the two moderately susceptible species, *Orbicella* spp. and *S. siderea* (https://floridadep.gov/rcp/coral/documents/stony‐coral‐tissue‐loss‐disease‐sctld‐case‐definition), the first year SCTLD was observed at a site to the year prior using a nonparametric Wilcoxon signed rank test (data were not normal, Shapiro tests: *p* < .05). We also evaluated the impact of a significant decline in *Orbicella* spp. as a result of the regional cold‐water event in 2010 on the relationship between coral cover and gross carbonate production using linear regressions (LM; 1996–2009 vs. 2010–2019). Model residuals were not normally distributed (Shapiro–Wilk tests: *p* < .05), but the relationships were similar and still significant using ranked data (1996–2009: *F*_1,494_ = 790.5, *p* < .001; 2010–2019: *F*_1,458_ = 422.2, *p* < .001), suggesting the relationships are robust. Additionally, we used linear regressions to analyze trends from 1996 to 2019 in the percentage of sites with positive net carbonate production, and those that had net carbonate production comparable to the western Atlantic mean for 2009–2017 of 2.55 kg CaCO_3_ m^−2^ year^−1^ (Perry et al., 2018), using the subset of 32 sites from the Keys subregions that were surveyed during that entire period (model residuals were normally distributed, Shapiro–Wilk tests: *p* > .05). The overall results of all analyses were similar when only the 32 original CREMP sites were analyzed (see Supplementary Results & Discussion; dashed lines in Figure 2), indicating that the trends we describe below are robust to the addition of sites through time.

## 3 RESULTS

Net carbonate production at our sites throughout the FKRT ranged from −1.75 to 8.06 kg CaCO_3_ m^−2^ year^−1^ (Figures S1–S4; Table S6), with an average of 0.57 (± 0.04 SE) kg CaCO_3_ m^−2^ year^−1^ across all sites and years. This translates to an average estimated reef‐accretion potential of 0.53 mm y^−1^ (± 0.04) regionwide, with a range of −1.61 to 7.44 mm year^−1^ (Figure 2a; Figure S1a and Table S6). Compared with the offshore habitats, patch reefs had significantly higher net carbonate production (1.28 ± 0.09 vs. <0.12 kg CaCO_3_ m^−2^ year^−1^) and reef‐accretion potential (1.18 ± 0.08 vs. <0.11 mm year^−1^) on average (Figures S2 and S5a; LME_habitat_: *F*_2,40_ = 10.23, *p* < .001; Tukey test: *p* < .005). Net carbonate production and reef‐accretion potential were not significantly different among subregions (Figure S6a; LME_subregion_: *F*_3,40_ = 2.76, *p* = .09).

**FIGURE 3:**
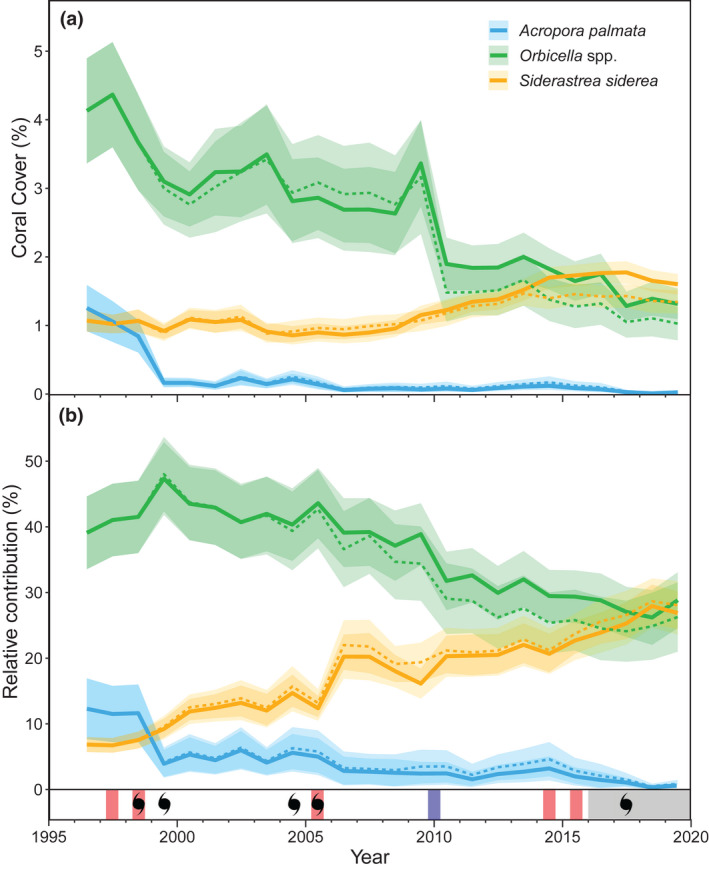
Trends in mean (solid lines) ± standard error (SE; shaded areas) (a) percent cover of *A. palmata*, *Orbicella* spp., and *S. siderea* and (b) their relative percent contribution to gross carbonate production. The timing of major high (red) and low (blue) thermal‐stress events, category 1–5 hurricanes (hurricane symbol), and the outbreak of SCTLD (gray shading) in the Florida Keys are shown on the bottom panel. Four separate hurricanes impacted the Florida Keys in 2005. Solid lines represent annual means for all 46 sites, whereas dashed lines are annual means for the 32 sites that were surveyed annually from 1996 to 2019. We note that the apparent increase in *Orbicella* spp. production in 2009 (a) is primarily an artifact of the addition of six new patch‐reef sites with relatively high coral cover in that year (see Supplementary Results & Discussion).

Regionwide, net carbonate production declined significantly over time from 1.33 (± 0.33) kg CaCO_3_ m^−2^ year^−1^ in 1996 to 0.22 (± 0.13) kg CaCO_3_ m^2^ year^−1^ in 2019 (LME_year_: *F*_23,887_ = 6.04, *p* < .001; Tukey test 1996 vs. 2019: *p* < .001), with reef‐accretion potential decreasing from 1.23 (± 0.31) to 0.20 (± 0.12) mm year^−1^. The largest decline occurred immediately following the 1997–1998 global bleaching event, with an ~76% reduction in net carbonate production on average across the FKRT (Figure 2a; Tukey test 1996 vs. 1999: *p* < .005). Net carbonate production experienced another significant decline following the 2010 cold‐water event both in the full dataset and the dataset with only the 32 sites surveyed each year from 1996 to 2019 (~42% and 55% declines, respectively; Tukey tests 2009 vs. 2010: *p* < .001), suggesting that this result is robust to the addition of six patch‐reef sites to the Keys subregions in 2009. We note that the addition of those sites was partially responsible for the apparent increase in regional coral cover (Figure 2c; particularly that of *Orbicella* spp. [Figure 3a]), and, therefore, carbonate production in the full dataset in 2009 (Figure 2a); however, there were also substantial increases in coral cover at two previously established patch‐reef sites (Admiral and Jaap Reef; see Figure S2) that also contributed to that trend. Net carbonate production experienced a final significant (~37%) decline following the outbreak of SCTLD at our sites between 2017 and 2019 (Wilcoxon signed rank test: W = 578, *p* = .002). There were no statistically detectable changes in net carbonate production associated with the hurricane impacts in 1999, 2004, 2005, and 2017, or the 2005, 2014, and 2015 thermal‐stress events (Tukey tests 1999 vs. 2000, 2003 vs. 2006, 2013 vs. 2016, and 2017 vs. 2018: *p* > .05).

The decline in net carbonate production from 1996 to present, drove a significant decrease in the percentage of sites with positive net carbonate production over time (LM: *F*_1,22_ = 87.45, *p* < .001, *r* ^2^ = 0.79) and those with net carbonate production greater than or equal to the western Atlantic mean of 2.55 kg m^−2^ year^−1^ reported by Perry et al. (2018) (Figure 2b; *F*_1,22_ = 90.97, *p* < .001, *r* ^2^ = .80). On average, ~80% (26) of the 32 reefs surveyed in 1996 had positive net carbonate production, and ~ 12.5% (4) had net carbonate production comparable to the western Atlantic mean of 2.55 kg m^−2^ year^−1^; however, by 1999, net carbonate production was positive at < 60% (19) of those reefs and < 40% (12) in 2019, with only ~9% (3) and ~3% (1) maintaining net carbonate production of 2.55 kg m^−2^ year^−1^ or higher in 1999 and 2019, respectively (Figure 2b). If the lower uncertainties (−1 SE) of the carbonate budgets are considered, the present status of Florida's reefs appears even more dire: only five of the 32 sites (~15%) in our study had net carbonate production rates that did not overlap with zero in 2019. All five were patch reefs and all but one was in the Lower Keys (Figure 1d; Figure S2 and Table S6). None of the reefs on the FKRT had lower uncertainties that were as high as 2.55 kg m^−2^ year^−1^ in 2019.

Bioerosion averaged 0.51 (± 0.01; range: 0.23–1.73) kg CaCO_3_ m^−2^ year^−1^ at our sites with parrotfishes and microbioerosion accounting for >96% of that total (Figures S7 and S8, Table S7; see Supplementary Results & Discussion). The necessity of using a single microbioerosion and sponge bioerosion estimate for each site and the time‐averaged estimates of parrotfish bioerosion (Table S2) limited our ability to quantify changes in bioerosion through time. We, therefore, do not explore temporal variability in bioerosion in this study.

As a result of the limited temporal variability in our bioerosion estimates, changes in net carbonate production primarily reflected trends in coral cover (Figure 2c; Figures S2–S4). Regionwide, the estimated coral cover threshold for maintaining positive net carbonate production was 6% (LME model predictions; patch reefs: 4–5%; offshore shallow: 10%; offshore deep: 5–6%). Our results suggest that changes in three coral taxa—*A. palmata*, *Orbicella* spp., and *S. siderea*—all of which experienced significant changes in both percent cover and their relative contribution to carbonate production from 1996 to 2019 (Figure 3), were the primary drivers of changes in net carbonate production on the FKRT.

Although acroporid populations were already low at the onset of this study, the mean cover of *A. palmata* was 3.34% (± 1.08) at our offshore shallow habitats in 1996 (its cover was negligible in other habitats; Figure 3a; Figure S9a; LME_habitat_: *F*_2,40_ = 7.90, *p* = .001 and *F*_2,40_ = 8.69, *p* < .001, for cover and contribution to carbonate production, respectively; Tukey test: *p* < .005). Following the 1997–1998 bleaching event, however, *A. palmata* cover in those habitats declined to <0.5% through the FKRT (Figure 3a; Figure S10a; LME_year_: *F*_23,887_ = 4.99, *p* < .001; Tukey tests 1996 vs. 1999: *p* < .001) and its contribution to gross carbonate production likewise decreased from ~12% (0.24 ± 0.09 kg m^−2^ year^−1^) in 1996 to <4% (0.03 ± 0.02 kg m^−2^ year^−1^) in 1999 (Figure 3b; Figure S11b; *F*_23,887_ = 3.22, *p* < .001; Tukey test 1996 vs. 1999: *p* < .001). The cover of *A. palmata* and its contribution to carbonate production remained low after this time, with no statistically detectable impacts of the later thermal stress events or hurricanes (Tukey tests 1999 vs. 2000, 2003 vs. 2006, 2009 vs. 2010, 2013 vs. 2016, and 2017 vs. 2018: *p* > .05; however, Tukey tests 1996 vs. 2019: *p* < .05). There were no statistically detectable effects of subregion on *A. palmata* (Figure S10a; LME_subregion_: *F*_3,40_ = 0.58, *p* = .63 and *F*_3,40_ = 0.57, *p* = .64 for cover and percent production, respectively).

*Orbicella* spp. were the dominant corals in our study, with an average of 2.38% (± 0.15) cover and a carbonate production rate of 0.57 (± 0.04) kg m^−2^ year^−1^ regionwide from 1996 to 2019; however, the cover of this taxon declined significantly through time across subregions and habitats (Figure 3a; Figures S9b, S10b, and S11b; LME_year_: *F*_23,887_ = 3.25, *p* < .001; Tukey test 1996 vs. 2019: *p* < .01; LME_subregion_: *F*_2,40_ = 1.14, *p* = .34; LME_habitat_: *F*_3,40_ = 1.82, *p* = .18). Although average *Orbicella* spp. cover declined from ~4 to 3% after the 1997–1998 bleaching event, that change was not statistically detectable nor were the impacts of hurricanes or the later coral bleaching events (Tukey tests 1996 vs. 1999, 1999 vs. 2000, 2003 vs. 2006, 2013 vs. 2016, and 2017 vs. 2018: *p* > .05). There was, however, a significant decline in *Orbicella* spp. following the 2010 cold event when its average cover fell below 2% (Figure 3a; Tukey test 2009 vs. 2010: *p* < .001). Although there were significant changes in the contribution of *Orbicella* spp. to carbonate production across all subregions and habitats (Figure 3b; Figures S9b and S10b; LME_year_: *F*_23,887_ = 2.14, *p* = .002; LME_subregion_: *F*_2,40_ = 2.09, *p* = .12; LME_habitat_: *F*_3,40_ = 0.66, *p* = .52), temporal changes were not statistically detectable in the pairwise comparisons associated with any of the identified disturbance events or between 1996 and 2019 (Tukey tests: *p* > .05); however, evaluation of the fixed effects of years in the LME model indicates that a decline in the contribution of *Orbicella* spp. to carbonate production occurred after the 2010 cold event (fixed effects of 2010, 2012, and all years from 2014 to 2019: *p* < .05). The reduction in *Orbicella* spp. populations in 2010 coincided with a decrease in the slope of the relationship between coral cover and gross carbonate production (Figure 2d; 1996–2009: 0.16 ± 0.004; 2010–2019: 0.13 ± 0.003). There was also a significant decline in *Orbicella* spp. cover following the outbreak of SCTLD at our sites (Wilcoxon signed rank test: W = 102, *p* = .002).

While the contribution of those reef‐building species was declining, the cover of the stress‐tolerant coral *S. siderea* increased significantly over time (Figure 3a; from ~1 to 1.6% cover; LME_year_: *F*_23,887_ = 2.47, *p* < .001). Because the year‐to‐year increases were small and gradual, there were no statistically detectable changes in *S. siderea* cover associated with any of thermal‐stress events or hurricanes or between 1996 and 2019 (Tukey tests: *p* > .05); however, the fixed effects of year in the LME model indicate that *S. siderea* cover was significantly higher from 2013 to 2018 (*p* < .05) than in 1996. As a result of the increase in *S. siderea* cover over time, its contribution to gross carbonate production also increased significantly, from just ~7% (0.09 ± 0.02 kg m^−2^ year^−1^) in 1996 to ~27% (0.13 ± 0.02 kg m^−2^ year^−1^) in 2019: a level similar to the ~29% (0.31 ± 0.09 kg m^−2^ year^−1^) contribution of *Orbicella* spp. in 2019 (Figure 3b; Figure S11b; LME_year_: *F*_23,887_ = 3.84, *p* < .001; Tukey test 1996 vs. 2019: *p* < .001). There was not a significant change in *S. siderea* cover or its contribution to carbonate production following the 1997–1998 bleaching event (Tukey test 1996 vs. 1999: *p* > .05), the 2010 cold event (Tukey test 2009 vs. 2010: *p* > .05), the hurricanes in 1999 and 2017 (Tukey test 1999 vs. 2000 and 2017 vs. 2018: *p* > .05), the coral‐bleaching events in 2014 and 2015 (Tukey test 2013 vs. 2016: *p* > .05), or the outbreak of SCTLD at our sites (Wilcoxon signed rank test: W = 161, *p* = .28); however, there was a significant increase in the relative contribution of *S. siderea* to carbonate production following the 2004–2005 hurricane season and the 2005 coral‐ bleaching event (Tukey test 2003 vs. 2006: *p* < .05). On average, *S. siderea* cover was highest in patch‐reef habitats (Figure S9c; LME_habitat_: *F*_2,40_ = 14.73, *p* < .001, Tukey test: *p* < .001), and its contribution to carbonate production was lowest in offshore shallow habitats (LME_habitat_: *F*_2,40_ = 3.83, *p* = .03; Tukey test: *p* < .05). Both cover and the relative contribution of *S. siderea* to carbonate production were lower in the Dry Tortugas subregion (Figure S10c; LME_subregion_: *F*_3,40_ = 9.02, *p* < .001 and *F*_3,40_ = 9.52, *p* < .001, respectively; Tukey test: *p* < .05 for all pairwise comparisons except Dry Tortugas vs. Lower Keys for relative carbonate production). We note that there were also increases in the relative contribution of the weedy coral *P. astreoides* to gross carbonate production over time (Figure S11c; LME_year_: *F*_23,887_ = 2.54, *p* < .001; Tukey test 1996 vs. 2019: *p* < .001); however, there were no consistent temporal changes in its cover (see Supplementary Results & Discussion).

**FIGURE 4:**
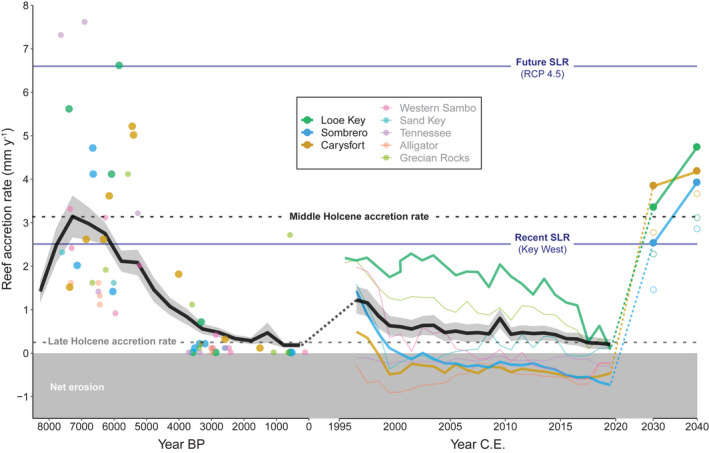
Estimates of past, present, and future reef accretion in the Florida Keys. Reef‐accretion potential estimates from this study (colored lines in middle panel) are compared with rates of reef accretion during the Holocene (8500 years before present [BP] to ~1950, which is the limit of radiocarbon dating and designated as “0” in this plot) reconstructed using reef cores (points in left panel; Toth, Kuffner, & Stathakopoulos, 2018) for sites that were evaluated in both studies. The dashed black line between the Holocene and the carbonate budget reconstructions highlights the ~1 mm year^−1^ difference between historic accretion estimates from geological versus ecological data. This difference is used to define the lower uncertainty (open circles) of the future accretion projections. Regionwide mean (black line) ± standard error (SE; gray shading) trends in reef accretion for both time periods are also plotted. Mean rates of reef accretion during the peak of regional reef accretion in the Middle Holocene (~7000 BP) and after reef accretion largely terminated ~3000 BP (Late Holocene) are indicated by horizontal dashed lines. Past changes are compared with projected increases in mean reef‐accretion potential under future coral‐restoration scenarios based on the 10‐ and 20‐year coral restoration targets of the Mission: Iconic Reefs initiative at three reefs (Table S4; NOAA, 2021). Filled circles represent optimal estimates of future reef‐accretion potential, whereas open circles account for the estimated ~1 mm year^−1^ erosion not quantified by the budgets. Trends in reef accretion are compared with mean rates of recent sea‐level rise (SLR; https://tidesandcurrents.noaa.gov/, station ID: 8724580 [Key West]) and projected rates of future (to 2100) SLR under Representative Concentration Pathway 4.5 (Perry et al., 2018) for the Florida Keys (horizontal blue lines).

We show that the restoration of *Acropora* and *Orbicella* spp. corals has the potential to significantly increase reef‐accretion potential at Looe Key, Sombrero, and Carysfort reefs (Table S4). Our carbonate budget models predict that if the M:IR coral cover targets for those taxa are met, average reef‐accretion potential at those sites could increase to 2.52–3.84 mm y^−1^ by 2030 (Phase 1) and 3.91–4.73 mm year^−1^ by 2040 (Table S4; Figure 4). Increases in *A. cervicornis* would have the largest impacts on reef‐accretion potential (but see discussion in section 4.3), followed by *A. palmata* and *Orbicella* spp., and 5% increases in any of those taxa could result in positive reef‐accretion potential on average at all three sites (Table S4). In contrast, increases in *S. siderea* would have negligible impacts on reef‐accretion potential.

## 4 DISCUSSION

The FKRT experienced a significant, regionwide decline in reef‐accretion potential from 1996 to 2019. Whereas most reefs had positive net carbonate production in 1996, by 2019, bioerosion was the dominant process on at least two‐thirds of reefs in the region (Figure 2b; Figures S1–S4). Although shifts from reef accretion to reef erosion are becoming an increasingly common trend globally (Estrada‐Saldívar et al., 2019; Januchowski‐Hartley et al., 2017; Perry et al., 2013), our study suggests that contemporary reef‐building capacity on the FKRT is especially low (Figure 2b; cf. Perry et al., 2018).

### 4.1 Ecological and environmental drivers of carbonate budgets

The decline in net carbonate production in our study largely reflects the decadal‐scale loss of reef‐building corals on the FKRT (Courtney et al., 2020; Ruzicka et al., 2013; Toth et al., 2014, 2019; cf. Perry et al., 2015). The more minor contribution of bioerosion in our study compared with most previous studies from Florida and the broader western Atlantic (Table S7; cf. Enochs et al., 2015; Manzello et al., 2018; Perry et al., 2018), resulted in a relatively low, 6%, coral cover threshold for maintaining positive net carbonate production on the FKRT compared with ~10% threshold estimated for the broader western Atlantic (Perry et al. 2013). Nonetheless, by 2019, a series of disturbances had caused coral cover on many reefs in the region to fall below that critical level.

For most reefs around the world, the increasing frequency and intensity of thermal extremes has been the dominant driver of coral mortality in recent decades (Bove et al., 2022; Bruno et al., 2019; Hughes et al., 2018). The shallow‐water habitats of the FKRT have warmed by ~0.8°C over the last century (Kuffner et al., 2015), with an estimated 0.57°C increase from 1993 to 2020 alone (Bove et al., 2022). There has also been a more than 20‐fold increase in the number of high‐temperature anomalies since the mid‐1990s (Manzello, 2015) and, since 2010, the frequency of “marine heat waves” has more than doubled (Bove et al., 2022). Additionally, unlike most tropical reefs, reef development in subtropical environments like south Florida is also limited by periodic impacts of winter cold‐water extremes (Colella et al., 2012; Lirman et al., 2011; Toth et al., 2021; Toth, Kuffner, & Stathakopoulos, 2018).

The most substantial reduction in coral cover, and consequently, carbonate production in our study occurred after the global 1997–1998 El Niño event (Figures 2 and 3). At our sites, thermal stress during that event caused substantial coral bleaching and bleaching‐related mortality (Ruzicka et al., 2013), and largely eliminated residual populations of the once‐dominant reef‐crest engineer, *A. palmata* (Figure 3a), that had survived the impacts of white‐band disease and cold‐water events in the 1970s (Precht & Miller, 2007). The most striking change in *Orbicella* spp., the dominant coral at our sites, occurred after an extreme winter cold‐water event in 2010 (Figure 3; Colella et al., 2012; Lirman et al., 2011). That event caused dramatic coral mortality in previously resilient inshore, patch‐reef environments of the Middle and Upper Keys (Figure S2; Colella et al., 2012; Guest et al., 2018), with cold‐sensitive *Orbicella* spp. suffering close to 100% mortality in some locations (Colella et al., 2012; Lirman et al., 2011). Finally, following the anomalously warm temperatures in 2014 and 2015 (Manzello, 2015), SCTLD began decimating the remnant coral populations on Florida's reefs (Muller et al., 2020; Precht et al., 2016). Interestingly, although both *Orbicella* spp. and *S. siderea* have similar susceptibilities to SCTLD (Muller et al., 2020; https://floridadep.gov/rcp/coral/documents/stony‐coral‐tissue‐loss‐disease‐sctld‐case‐definition), only *Orbicella* spp. experienced a significant decline in cover following the SCTLD outbreak, and that decline was associated with a significant reduction in regional net carbonate production. Although hurricanes can have significant local‐scale impacts on coral cover (Gardner et al., 2005), our study supports the conclusion that the eight hurricanes that impacted the FKRT from 1996 to 2019 had minimal impacts on regional coral cover or carbonate production (Courtney et al., 2020; Kobelt et al., 2020). We note that these hurricanes did cause significant declines in the populations of other reef biota in the region (Ruzicka et al., 2013), including important bioeroders like *D. antillarum* (Kobelt et al., 2020). Hurricane impacts could also explain the apparent decline in parrotfish bioerosion in our study following the 2004–2005 hurricane season (Figures 2a and 3; Figures S7a and S8a). Overall, however, the temporal trends in our carbonate budgets suggest that, together, warm and cold extremes and coral disease have been the primary drivers of declines in reef‐building corals and, therefore, carbonate production in the region over the last 24 years (Figure 3; cf. Courtney et al., 2020).

*Orbicella* spp. and *A. palmata* have been the dominant reef‐building corals throughout the western Atlantic for at least 600,000 years (Kuffner & Toth, 2016; Toth et al., 2019). The disproportionate loss of these ecosystem engineers in recent decades has driven an unprecedented shift in reef composition that is accelerating reductions in reef‐building capacity (Courtney et al., 2020; Estrada‐Saldívar et al., 2019; Perry et al., 2015; Toth et al., 2019). On the FKRT, declines in *Orbicella* spp. and *A. palmata* have been associated with relative increases in the cover of the stress‐tolerant coral *S. siderea* (Figure 3a; Burman et al., 2012; Courtney et al., 2020; Toth et al., 2014, 2019) and an increased role of both *S. siderea* and *P. astreoides* in carbonate production (Figure 3b; Courtney et al., 2020). Because the calcification rates of *S. siderea* and *P. astreoides* are two to three times lower than those of the reef‐building corals they replaced (Courtney et al., 2021), their net contribution to reef building remains minimal (Figures S11b and S12). We show that the dwindling role of *Orbicella* spp. and *A. palmata* on Florida's reefs has fundamentally changed the relationship between coral cover and carbonate production (Figure 2d; cf. Courtney et al., 2020; Perry et al., 2015). This result supports the conclusion that coral cover alone is an insufficient predictor of carbonate production (Alvarez‐Filip et al., 2013; Courtney et al., 2020; Perry et al., 2015) and highlights the importance of reef‐building species in maintaining positive carbonate budgets now and in the future (de Bakker et al., 2019; Kennedy et al., 2013; Toth et al., 2019).

The transformation of Florida reef assemblages has also caused the spatial homogenization of reef habitats (Burman et al., 2012). Consequently, whereas there was high variability in carbonate production among sites in 1996, many of Florida's reefs have now converged towards a state of diminished reef‐building capacity (Figures S1–S4; cf. Estrada‐Saldívar et al., 2019). One striking exception are the patch‐reef habitats of the Lower Keys (Figure 1). Patch‐reef habitats throughout the FKRT generally had higher coral cover and, therefore, higher net carbonate production than offshore habitats (Figure S5), and coral populations in those inshore environments were more resilient to the impacts of the 1997–1998 bleaching event (Ruzicka et al., 2013); however, the proximity of the Middle and Upper Keys patch reefs to highly variable water masses from the Florida Bay (Figure 1) made corals there particularly vulnerable to the impacts of the 2010 cold‐water event (Colella et al., 2012; Lirman et al., 2011). In contrast, all the Lower Keys patch reefs have maintained positive carbonate budgets despite the suite of disturbances in recent decades (Figure 1; Figure S2 and Table S6). We hypothesize that the combination of relatively low water clarity (lower irradiance) of inshore environments on the FKRT, in general, and distance of the Lower Keys patch reefs from tidal passes to the Florida Bay, in particular, have resulted in these reefs having high resilience to both high and low thermal anomalies, respectively, suggesting that they may be important targets for future management (Barnes et al., 2015; Elahi et al., 2022; Guest et al., 2018; Sully & van Woesik, 2020). We note that SCTLD was first observed in the Lower Keys during the final year of our study in 2019, so we likely did not capture its full impact on coral cover at our sites there.

### 4.2 Quantifying changes in reef accretion

One way to provide context for the recent decline in reef‐building capacity on the FKRT is to compare decadal‐scale reconstructions of reef‐accretion potential to accretion trends over millennial timescales (Toth et al., 2021; Toth, Kuffner, & Stathakopoulos, 2018). During the peak of regional reef growth ~7000 years ago (the Middle Holocene), when the climate was optimal for reef development, accretion rates on the FKRT averaged ~3 mm year^−1^; however, as temperatures cooled and became more variable, Florida's subtropical environment became marginal for reef growth, and by ~3000 years ago, accretion rates on the FKRT were negligible at <0.5 mm year^−1^ (Toth et al., 2021; Toth, Kuffner, & Stathakopoulos, 2018). By 2019, reef accretion potential was negative for most reefs in our study and averaged just 0.20 mm year^−1^ regionwide (Figure 4). The millennial‐to‐decadal‐scale history of reef building on the FKRT supports the hypothesis of Toth et al. (2018) that although Florida's reefs have been balanced at the precipice between accretion and erosion for thousands of years, it was not until the loss of reef‐building corals in recent decades that they were pushed past that tipping point and into a state of net erosion (Figure 4).

Interestingly, although the average regional accretion rate over the last 1000 years was just 0.17 mm year^−1^, average reef‐accretion potential in 1996 was estimated to be substantially higher at 1.23 mm year^−1^ (see dashed black line in Figure 4). The fact that the reef‐accretion potential of some reefs on the FKRT at the beginning of our study was nearly as high as the natural baseline of the Middle Holocene (i.e., Figure 4; Figures S1–S4) suggests that, in a few locations, there could have been a historical resurgence of accretion following the hiatus during recent millennia; however, a reconstruction of landscape‐scale net reef‐elevation change in the Florida Keys since the 1930s found that, on average, shallow‐water reef habitats eroded at a rate of −4.5 to −1.5 mm year^−1^ over this period (Yates et al., 2017). Although sediment transport likely played a significant role in the erosion measured in that study, it also supports the conclusion that regional reef‐framework accretion was likely not as high as our carbonate budgets suggest.

Instead, the discrepancy between accretion metrics for the FKRT supports the conclusion that reef‐accretion potential estimated by census‐based carbonate‐budget studies almost certainly underestimates total reef erosion and, therefore, overestimates realized reef accretion (Browne et al., 2021; Perry et al., 2018). One reason for this is the inherent complexity of fully parameterizing the spatial and temporal variability in both bioerosion and carbonate production with snapshot surveys and generalized rates of ecological processes (Lange et al., 2020). A related possibility is that because of the limitations in quantifying bioerosion using historical datasets (Table S2), our study could have underestimated the contribution and variability of bioerosion on the FKRT; however, the fact that the range of bioerosion rates among our sites was similar to ranges determined for other locations in outh Florida suggests that our estimates are likely reasonable (see Supplementary Results & Discussion). There are also important interactions between constructive and destructive processes that are not fully understood, such as the impact that habitat degradation (i.e., reef flattening) has on habitat partitioning for bioeroders and the relationship between topographic complexity and erosion rates (Kuffner et al., 2019; Lange et al., 2020; Perry et al., 2014; Perry & Alvarez‐Filip, 2019).

An even more significant source of uncertainty in census‐based carbonate budget studies is the omission of physical erosion, sediment transport, and chemical dissolution (Browne et al., 2021). The geological process of reef accretion is the time‐averaged result of *all* the constructive and destructive processes that occur on a reef over a scale of centuries‐to‐millennia. Whereas carbonate budget studies necessarily focus on identifying and/or projecting measurable trends in the most dominant short‐term processes, over longer timescales, episodic disturbances become increasingly important (Buddemeier & Hopley, 1988; Hubbard, 1988). Given the mismatch in processes and timescales evaluated in geologic versus census‐based carbonate budget estimates of reef accretion, the difference between measured rates of reef framework accretion during the last millennia and estimated reef accretion potential at the beginning of our study (Figure 4) is not surprising (Browne et al., 2021; Hubbard, 1988; Roff, 2020).

Although our results suggest that direct comparisons across disparate timescales should be treated with some caution (Browne et al., 2021), our study also offers a unique opportunity to quantify the contribution of erosive processes not typically captured by the ReefBudget methodology (Perry & Lange, 2019) to net reef accretion. We hypothesize that the ~1 mm year^−1^ (1.15 kg CaCO_3_ m^−2^ year^−1^) offset between recent accretion measured using reef cores (1950 C.E.) and estimated rates of reef accretion potential from the carbonate budgets (1996 C.E; Figure 4) provides a first‐order approximation of the contribution of physical and chemical erosion on the FKRT that can be treated as the uncertainty associated with using simplified census‐based carbonate budgets to estimate long‐term reef accretion. This estimate is likely conservatively low because populations of rapidly calcifying acroporids largely declined on the FKRT before the onset of our study in 1996 (Precht & Miller, 2007), which suggests that net production would have been higher at the end of the geologic record in 1950.

To illustrate the impact that this uncertainty would have on our results, we recalculated our estimates of present‐day reef accretion and the thresholds of coral cover needed to support net positive carbonate production after incorporating the −1.15 kg CaCO_3_ m^−2^ year^−1^ of erosion that could theoretically be missing from our budgets. That analysis suggests that average accretion at our sites in 2019 could be as low as −0.93 (±0.13 SE; range: −1.94 to 1.83) and that a higher threshold of coral cover would be needed to support positive budgets (LME predictions: 15% vs. 6% in the original model). If our 1 mm year^−1^ estimate of longer‐term physical and chemical erosion processes is reasonable, then previous estimates of reef‐accretion potential using carbonate budget methods may have, likewise, substantially overestimated true accretion rates (Perry et al., 2018); 1 mm year^−1^ represents 36% of the global median reef‐accretion potential rate of 2.80 mm year^−1^ (Cornwall et al., 2021) and 53% of the mean western Atlantic reef‐accretion potential rate of 1.87 mm year^−1^ (Perry et al., 2018). Ours is one of the few studies to date that has attempted to quantify the relative contribution of biological, chemical, and physical erosion in reef environments, and there is critical need to combine and cross‐validate methods for quantifying reef‐framework construction and erosion to develop more comprehensive assessments in the future (Courtney et al., 2016; Lange et al., 2020). We reiterate that census‐based carbonate budgets still provide valuable estimates of changes in the relative importance of biologically driven constructive and destructive processes through time, but caution the interpretation of these results to be representative of net reef accretion over longer timescales (Browne et al., 2021).

Although there are still some “oases” within the degraded reefscapes of the Florida Keys (Figure 1d; Courtney et al., 2020; Elahi et al., 2022; Guest et al., 2018), our estimate of average reef‐accretion potential on the FKRT at present of 0.20 mm year^−1^ is substantially lower than most other locations around the world (Cornwall et al., 2021; Perry et al., 2018). With the putative additional contributions of physical and chemical erosion, it is likely that reef building on the FKRT has declined even more dramatically than our study suggests, and erosion is now the dominant process regionwide (i.e., reef accretion is estimated at −0.93 mm year^−1^, on average). As a result, the persistence of the key ecological, geological, and socioeconomic functions of Florida's reefs is in jeopardy (Perry & Alvarez‐Filip, 2019; Woodhead et al., 2019). Given the central role of climate in controlling the growth of Florida's reefs both in recent decades and over millennial timescales (Precht & Miller, 2007; Toth et al., 2019, 2021; Toth, Kuffner, & Stathakopoulos, 2018), anthropogenic climate change will undoubtedly continue to limit regional reef building in the future. Climate change is predicted to both accelerate declines in carbonate production and increase bioerosion and carbonate dissolution on reefs, which would further depress regional carbonate budgets (Cornwall et al., 2021; Enochs et al., 2015; Eyre et al., 2018; Perry & Alvarez‐Filip, 2019). Clearly, there is an urgent need to rebalance the carbonate budgets of the FKRT before the reef frameworks that took thousands of years to build are lost.

### 4.3 Can coral restoration reverse long‐term declines in reef accretion?

One way that coral‐reef managers can work to tip the balance of reef processes back towards accretion is through restoration of reef‐building coral populations. Global‐scale action on climate change is critical to ensuring the persistence of reef ecosystems in the long term, but in the near term, restoration can jump‐start coral recovery while the larger threats to coral reefs are addressed (Boström‐Einarsson et al., 2020; Bruno et al., 2019; Hein et al., 2021). This may be especially important for locations like the FKRT that have shown little capacity for natural recovery following disturbance events (Figures 1, 2, 3). There remain a number of important questions about how and whether the small‐scale efforts that have characterized most coral restoration to date can be scaled up to the levels required to maintain key ecosystem functions and services in practice (Boström‐Einarsson et al., 2020; Hein et al., 2021; Storlazzi et al., 2021). Nonetheless, our study suggests that, in theory, restoration could help to mitigate long‐term declines in reef accretion in some locations. For example, if M:IR succeeds in increasing cover of reef‐building corals to ~15% by 2030 (Phase 1) and ~ 25% by 2040 (Phase 2; NOAA, 2021), coral cover at the seven reefs in the Florida Keys restored through that initiative would meet or exceed our high‐end estimate of the regional threshold for positive net carbonate production of 15%.

Our comparison of various restoration scenarios suggests that increases in *A. cervicornis*, the species most commonly used for coral restoration in the western Atlantic to date, would produce the most substantial short‐term increase in reef accretion potential (Table S4), but its paucity throughout the Holocene reef framework in Florida indicates that it likely contributes little to long‐term reef building in most locations (Toth et al., 2019). The relatively minimal impact of simulated increases in *S. siderea* cover on estimated reef‐accretion potential also reinforces the conclusion that despite its increasing dominance in south Florida in recent decades (Burman et al., 2012; Toth et al., 2014), *S. siderea* is a poor substitute for the reef‐building species it has replaced (Table S4; Figures S11b and S12; Alvarez‐Filip et al., 2013; Courtney et al., 2020; Kennedy et al., 2013). Living cover of any species can help mitigate erosion (Kuffner & Toth, 2016) and potentially maintain stable, albeit lower, rates of carbonate production (Courtney et al., 2020); however, restoration of *A. palmata* with supplemental enhancement of massive reef‐building corals like *Orbicella* spp. (e.g., via emerging microfragmentation techniques [Page et al., 2018]), particularly in patch‐reef habitats where acroporids are uncommon, likely provides the most optimal long‐term strategy for reviving accretion on Florida's reefs (Figure 4; Table S4). Kuffner et al. (2020) recently demonstrated that outplanting of *A. palmata* can even be successful in habitats where it was historically absent in south Florida.

We show that if M:IR meets its targets of restoring *Acropora* and *Orbicella* spp. populations, reef accretion potential at Looe Key, Sombrero, and Carysfort reefs could be restored to levels comparable to Middle Holocene baselines, even accounting for the hypothesized ~1 mm year^−1^ uncertainty in those estimates (open circles in Figure 4). By 2040, our high‐end estimates of reef‐accretion potential may also be sufficient to allow those restored reefs to come close to keeping pace with future sea‐level rise, if CO_2_ emissions are reduced (i.e., RCP 4.5; Figure 4; cf. Perry et al., 2018), but with the additive impacts of physical and chemical erosion and under less optimistic emission scenarios, additional interventions may be necessary. Although the M:IR restoration plan does account for the inevitability of continued coral mortality as a result of coral bleaching, disease, or other stochastic events like storms (NOAA, 2021), by increasing the frequency and severity of those disturbances, climate change will likely make it increasingly difficult for managers to mitigate the impacts of mortality events and maintain restored coral cover in the future. Furthermore, our analysis does not consider the potential for future climate change to alter the ecological drivers of carbonate budgets, for example, by causing declines in coral growth or increases in bioerosion and chemical dissolution (Cornwall et al., 2021; Enochs et al., 2015; Lange et al., 2020). As a result, our estimates of the possible impacts of restoration on reef‐accretion potential are likely overly optimistic, particularly in the longer term or under high‐emission climate‐change scenarios.

In conclusion, our study provides an important first‐order estimate of how restoration could reverse long‐term declines in the accretion of some of Florida's reefs; however, there is also a critical need to develop more complex, nuanced models to estimate the likely impacts of restoration under various climate‐change scenarios and to evaluate the real‐world impacts of ongoing restoration activities on carbonate budgets and reef growth. Whereas some of Florida's reefs have been relatively resilient to thermal stress in the past, particularly those in the inshore environments of the Lower Keys, the increasing threat of anthropogenic climate change will make active management of even the most resilient reefs increasingly important in the future (Hein et al., 2021). Despite the expanding scale and scope of coral‐reef restoration in south Florida in recent years (NOAA, 2021), current restoration efforts on the FKRT are likely not sufficient to reverse historic declines on a regional scale, especially with the ongoing threat of anthropogenic climate change. Nonetheless, our study suggests that it may be possible to rebalance the carbonate budgets of some of Florida's reefs to help restore reef‐building capacity and maintain the key ecosystem services they support on a local scale, while global‐scale action is taken to reduce carbon emissions.

## Supporting information


Appendix S1

Table S6

## Data Availability

Supporting data and the R script used to calculate and analyze the carbonate budgets in this study are provided in a U.S. Geological Survey software release available at https://doi.org/10.5066/P9APPZHJ
