# Supplementary material for: The past, present, and future of coral reef growth in the Florida Keys
Source: Glob Chang Biol. 2022 Jul 5;28(17):5294–309. doi: 10.1111/gcb.16295 (PMC9542952; doi:10.1111/gcb.16295)
Supplement: Supplementary file 1 — Appendix S1 [file GCB-28-5294-s002.pdf]

Supporting Information

for

The past, present, and future of coral reef growth in the Florida Keys

Lauren T. Toth[1], Travis A. Courtney[2,3], Michael A. Colella[4], Selena A. Kupfner Johnson[1], and Robert R. Ruzicka[4]

[1]U.S. Geological Survey, St. Petersburg Coastal and Marine Science Center, St. Petersburg, FL, 33701, U.S.A.

[2]Scripps Institution of Oceanography, University of California San Diego, La Jolla, CA, 92093, U.S.A.

[3]Department of Marine Sciences, University of Puerto Rico Mayagüez, Mayagüez, PR, 00681

[4]Fish & Wildlife Research Institute, Florida Fish & Wildlife Conservation Commission, St. Petersburg, FL, 33701, U.S.A.

Supplementary Methods

The following Supplementary Methods provide additional details about the data sources and their limitations, our analysis, and sources of uncertainties. All of the supporting data for this study as well as the annotated R code used to calculate the carbonate budgets and perform statistical analyses can be found in the USGS Software Release associated with this publication (Toth & Courtney, 2022; https://doi.org/10.5066/P9APPZHJ).

CREMP surveys/Site Descriptions

In 1996, the Florida Fish and Wildlife Research Institute (FWRI) Coral Reef Evaluation and Monitoring Project (CREMP) began annual surveys (typically May–September each year) of 34 reef sites throughout the Florida Keys. Site selection for CREMP followed a stratified random design based on the U.S. Environmental Protection Agency's Environmental Mapping and Assessment Program (https://archive.epa.gov/emap/archive-emap/web/html/index.html), wherein sites were partitioned across subregions and habitats using existing regional habitat maps and aerial photographs as a guide (Porter, 2001). This resulted in the establishment of 10 sites in the Upper Keys subregion, eight in the Middle Keys, and 16 in the Lower Keys. Of these, 12 are in offshore shallow habitats (1.8–7.3 m water depth), 11 are in offshore deep habitats (10.7–16.5 m), and 9 are in patch-reef habitats (1.8–10.4 m water depth). Offshore shallow sites and offshore deep sites are paired at the same reefs except for Grecian Rocks, which only has a shallow location. Two of the sites established in 1996 were excluded from our study because they are "back-country" patch-reef sites located north of the islands of the Florida Keys in Florida Bay. One patch-reef site and two offshore deep sites were added in the Dry Tortugas subregion in 1999, another four patch-reef sites were added in 2004, and one additional patch reef-site was added in 2005. Finally, in 2009, three additional patch-reef sites were added in the Dry Tortugas, and two additional patch-reef sites were added in each of the Keys subregions to better capture the high variability within these habitats. A summary of the CREMP sites is provided in Table S1.

For our statistical analysis of the carbonate budgets (main text), we excluded the data from three Dry Tortugas sites: Black Coral Rock, Palmata Patch, and Prolifera Patch (the budgets for those sites are included in Table S6). The offshore deep site Black Coral Rock was excluded because it is located in a considerably deeper reef habitat (21.6 m) than any of the other sites in our study. Palmata Patch and Prolifera Patch were excluded because they represent more-or-less mono-specific assemblages (of *Acropora palmata* and *A. prolifera*, respectively) and, therefore, are not comparable to the other patch-reef habitats in our study. We include the remaining 46 sites in all of the statistical analyses presented in the main text with the exception of the analysis of temporal trends in the percentage of sites with a positive carbonate budget or net carbonate production of at least 2.55 kg m^{-2} y^{-1} (cf. Perry et al. 2018), for which we only included the 32 reef sites that were surveyed each year from 1996–2019 (i.e., the Dry Tortugas sites and the six patch reef sites added to the Keys subregion in 2009 were excluded).

We note that the geomorphology of the reefs in the Dry Tortugas is somewhat distinct from that of the Keys subregions. In particular, several of the "patch reef" sites in the Dry Tortugas

subregion—Temptation Rock, Mayer's Peak, The Maze, Davis Rock, and Texas Rock—are actually isolated reef pinnacles, ranging in depth from 7.0 to 12.8 m. Although these sites are not strictly equivalent to the patch reefs in the Keys subregion, for simplicity, the Dry Tortugas sites are fit by CREMP into the same habitat designations as sites in the Florida Keys. This did not affect the overall conclusions of the study, as all of our results were similar when just the data from the 32 original sites in the Keys subregions were considered (see Supplementary Results & Discussion).

Each CREMP site had between 2 and 4 replicate stations (permanent transects) that were surveyed each year. From 2001–2010, 1–2 fewer stations were surveyed at the majority of sites as a result of independent statistical evaluation of the data, which suggested that station reduction did not significantly alter the estimates of percent cover for stony corals (Wheaton et al., 2001). Those excluded stations were added back into the CREMP surveys in 2011 to increase the study's statistical power. The primary benthic surveys consisted of 40-cm wide videographic or photographic transects of each station. The length of the permanent transects at each station varied between 18 and 27 m, with a mean length of ~22 m. The specific lengths for each station were used to calculate survey areas for the carbonate budgets. At FWRI, point count analysis (using the program PointCount '99) of the 30 x 40 cm transect images was used to quantify the percent cover of stony coral species, other benthic taxa, and substrate (Ruzicka et al., 2009). For each image, 10 (1996–2006) or 15 (2007–present) points were analyzed or ~80–120 points m^{-2}, a density that was determined based on pilot studies and statistical simulations to provide sufficient power to capture major changes in benthic composition (cf. Pante & Dustan, 2012). CREMP also conducted censuses of *Diadema antillarum* and bioeroding sponges, as described in the sections below.

Carbonate Production

Benthic cover of coral taxa and crustose coralline algae from the CREMP surveys were used to estimate gross carbonate production at each CREMP station for each year between 1996 and 2019. Crustose coralline algae were not distinguished from "substrate" in the CREMP surveys until 2010. Additionally, the percent coverage of these taxa in 2010 were anomalously low compared with the following years, so the annual data on crustose coralline algae data were only used from 2011–2019. We used the station-level means (±standard error [SE]) for 2011–2019 for all other years from 1996–2010. We note that crustose coralline algae are difficult to distinguish from other carbonate substrates using underwater imagery, so our estimates of carbonate production by crustose coralline algae are likely conservatively low. With an average site-level production rate of just 0.01 (<0.01 SE) kg m^{-2} y^{-1}, crustose coralline algae accounted for less than 1% of carbonate production at our sites on average, making it a relatively minor component of the total carbonate budgets.

Calcification rates for each coral species and for crustose coralline algae were multiplied by their percent cover at each of the CREMP stations in each year to estimate gross carbonate production. We used the taxa-specific calcification rates without bioerosion (i.e., see subsequent calculations of bioerosion in this study) derived by Courtney et al. (2021) for the online benthic analysis program CoralNet (https://coralnet.ucsd.edu/). These data are available at

https://doi.org/10.5281/zenodo.5140477 and as part of the USGS Software Release (Toth & Courtney, 2022). Like the benthic coverage data in CoralNet, the point-count data collected by CREMP provide percent cover based on planar, rather than the three-dimensional surface areas that are specified by the ReefBudget methodology (Perry & Lange, 2019). The Courtney et al. (2021) modification to the ReefBudget methodology produces taxa-specific area-normalized calcification rates (kg $CaCO_3$ m^{-2} yr^{-1}) that take into account empirical data on the linear extension, skeletal density, colony size, and colony rugosity for each taxa in the CREMP dataset (see Courtney et al. [2021] for further details). Additionally, we applied several calcification rate substitutions for CREMP taxa that did not have data in Courtney et al. (2021): *Madracis decactis* calcification rates for the *Madracis decactis* complex, *Mycetophyllia lamarckana* calcification rates for the *Mycetophyllia lamarckiana* complex, *Oculina* spp. calcification rates for *Oculina robusta*, the mean of *Porites divaricata/furcata/porites* calcification rates for the *Porites porites* complex, and *Tubastrea* spp. calcification rates for *Phyllangia americana* (based on both being azooxanthellate species with similar morphologies).

We note that direct measurements of mean calcification rates (by the buoyant weighing method; Jokiel et al. [1978]) in the Florida Keys are available for several taxa—*Siderastrea siderea* (9.9 kg $CaCO_3$ m^{-2} y^{-1}; Kuffner, Hickey, & Morrison, 2013), *Orbicella* spp. (8.5–13.0 kg $CaCO_3$ m^{-2} y^{-1}; Kuffner et al., 2019; Manzello, Enochs, Kolodziej, & Carlton, 2015), *Porites astreoides* (11.3 kg $CaCO_3$ m^{-2} y^{-1}; Lenz, Bartlett, Stathakopoulos, & Kuffner, 2021), and crustose coralline algae (0.5 kg $CaCO_3$ m^{-2} y^{-1}; Kuffner et al., 2013); however, the calcification rates in those studies are generally similar to the western Atlantic means provided in Courtney et al. (2021; *S. siderea*: 8.4 kg $CaCO_3$ m^{-2} y^{-1}, *Orbicella* spp.: 24.1 kg $CaCO_3$ m^{-2} y^{-1}, *P. astreoides*: 10.2 kg $CaCO_3$ m^{-2} y^{-1}, coralline algae: 0.4 kg $CaCO_3$ m^{-2} y^{-1}), with the exception of *Orbicella* spp., which had depressed rates in the Florida Keys in recent years due to thermal stress (Kuffner et al., 2019; Manzello et al., 2015). We also acknowledge that coral calcification rates can vary substantially over space and time; however, data are not currently available to fully parametrize that variability. We therefore chose to incorporate the regional growth-rate data aggregated by Perry and Lange (2019) in Courtney et al. (2021) to maintain consistency in methodologies across the dataset. We assume that the mean ± uncertainties in calcification rates captured in the broader, regional dataset provided a conservative measure of the variability in calcification across the variety of reef habitats represented by the CREMP sites, which is a common limitation of census-based studies.

Urchin Bioerosion

During each year of the CREMP surveys *D. antillarum* were counted. From 1996–2010, *D. antillarum* individuals were counted within a 2 m x ~22 m belt transect at each survey station by two divers; in cases where there was disagreement between the observers, the mean of the two counts was recorded. After 2010, the survey area was reduced to 1 m x 10 m belt transects at each station with a single observer. To standardize the data, *D. antillarium* abundances were converted into densities (individuals m^{-2}) by dividing the number of urchins observed by the total survey area for each station in each year.

Whereas researchers recorded the total number of *D. antillarum* individuals observed beginning in 2009, from 1996–2008 if more than four individuals were observed, their abundance was recorded as “4”. For our analysis, we used the number of urchins, as recorded by CREMP, even though “4” may have underestimated true urchin density in the 1996–2008 dataset. Four or more *D. antillarum* were only observed in three instances during this time period (two stations at Western Sambo Shallow in 1998 and one station at Rock Key Shallow in 2008), so this methodological choice is likely not a significant source of uncertainty. After 2009, several sites—Palmata Patch, Prolifera Patch, and Loggerhead Patch in the Dry Tortugas and Red Dunn Patch in the Lower Keys—frequently had more than four *D. antillarum* observed within the 1 x 10 m belt transects; however, only Palmata and Prolifera Patch, both sites that were not included in statistical analyses, typically had abundances in excess of 10 urchins per station in multiple years.

The rate of bioerosion (g $CaCO_3$ urchin^{-1} d^{-1}) by individual *D. antillarum* urchins can be estimated based on their test size (mm) using the empirical relationship described in the ReefBudget v2 methodology (Perry & Lange, 2019):

$$\textit{D. antillarum} \text{ bioerosion rate} = 0.0029 * \text{test size}^{1.6624}$$

Test size of the urchins was not recorded in the CREMP surveys, however, so the census data could not be directly converted to bioerosion rates. Instead, we extracted *D. antillarum* size (cm) distributions measured at 235 sites across the Florida Keys in 2007 from Figure 3 in Chiappone et al. (2008) using WebPlotDigitizer (Rohatgi, 2021). The mid-point, lower bound, and upper bound of each size bin (0.5–10 cm) were converted to mm and input into the equation above to determine the bioerosion rate for each size class. Those rates were then multiplied by the percentage of individuals in the respective size bin from Chiappone et al. (2008) and summed to calculate a weighted average of *D. antillarum* bioerosion rate with lower and upper bounds for the Florida Keys. The data were converted into kg $CaCO_3$ urchin^{-1} y^{-1} and were multiplied by site-level mean (±SE) urchin densities to estimate mean (±SE) urchin bioerosion (kg $CaCO_3$ m^{-2} y^{-1}) for each CREMP site in each year.

The largest potential source of uncertainty in our estimates of urchin bioerosion stems from the fact that the abundance of bioeroding urchins other than *D. antillarum* (i.e., *Echinometra lucunter*, *Echinometra viridis*, and *Eucidaris tribuloides*) were not recorded in the CREMP surveys. For example, Manzello et al. (2018) found that *Echinometra* spp. were the dominant bioeroding urchins at Cheeca Rocks patch reef in the Upper Florida Keys. To evaluate this potential bias in our data, we counted all visible urchin species within the 40 cm wide photographs taken along the ~22 m transects at each station (used by CREMP to assess benthic cover) in 2017, paying particular attention to cryptic areas on the reef. Although this method is almost certainly less accurate than *in situ* urchin surveys in estimating total abundances (indeed, only 52 urchins were observed from the photographs versus 105 *D. antillarum* observed *in situ* in 2017), it does provide a means of assessing the relative abundance of urchin species. Across the 49 offshore and patch reef sites surveyed in 2017, we recorded a total of 52 urchins from photographic transects. Of those observations, 42 (81%) were *D. antillarum*, 10 (19%) were *Echinometra* spp., and no *Eucidaris tribuloides* were observed. This supports the conclusion that

urchins other than *D. antillarum* are generally not common at the CREMP sites, but it is also possible that the other species are simply too cryptic during the day to be accurately recorded with standard census methods. Overall, because *D. antillarum* is the most abundant urchin at the CREMP sites included in our study and because urchin bioerosion is a relatively minor component of western Atlantic reef carbonate budgets in general (Perry et al., 2014) and the Florida Keys in particular (Kuffner et al., 2019; Manzello et al., 2018), the omission of other bioeroding urchin species likely had a relatively minor impact on our carbonate budgets.

Sponge Bioerosion

From 2001–2009, the planar surface area of bioeroding sponges was recorded by diver surveys along three 1 m x ~22 m belt transects within each CREMP station at each site. Each time a bioeroding sponge was encountered along the transects, a 40 x 40 cm quadrat divided into 5 x 5 cm sections was placed over the colony, allowing the colony's area to be estimated to the nearest 2.5 cm^2 (i.e., ½ the dimensions of the smallest division in the quadrat; see Callahan [2005]). From 2001–2004, the planar surface area occupied by the species *Cliothosa delitrix*, *Cliona caribbaea*, and *Pione lampa* were recorded at each station. Beginning in 2005, observations of *Cliona varians* were also added to the surveys. The bioeroding sponges *Siphonodictyon* spp. were not included in any of the CREMP surveys; however, this species is not common on most reefs in the Florida Keys. For example, in surveys along 18, 0.25 x 10 m transects (45 m^2 total survey area) at Hen & Chickens reef, an Upper Keys patch reef, *Siphonodictyon* spp. only accounted for ~10.6 cm^2 surface area on the reef (0.002% cover) and contributed < 0.002 kg m^{-2} y^{-1} to the total carbonate budget (Kuffner et al., 2019). Previous studies have demonstrated that the two most common bioeroding sponges in the Florida Keys are *Cliothosa delitrix* and *Cliona varians* (Kuffner et al., 2019; Manzello et al., 2018), which were both included in the surveys starting in 2005. Therefore, to eliminate potential underestimation of sponge bioerosion due to the late addition of *Cliona varians* to the dataset, we used the mean (±SE) surface area of sponges recorded by CREMP from 2005–2009 to estimate sponge bioerosion.

We followed the ReefBudget v2 methodology (Perry & Lange, 2019) wherein sponge bioerosion was estimated by multiplying the total surface area of bioeroding sponges (converted to m^2) at each CREMP station by species-specific bioerosion rates and dividing that value by the total survey area. For the bioeroding sponges *Cliothosa delitrix, Cliona varians*, and *Cliona caribbaea* we used the species-specific bioerosion rates based on de Bakker et al. (2018) as reported in Perry and Lange (2019) (*Cliothosa delitrix*=10.48 kg m^{-2} y^{-1}, *Cliona varians*=4.23 kg m^{-2} y^{-1}, *Cliona caribbaea*=4.67 kg m^{-2} y^{-1}). The sponge *P. lampa* is not included in the ReefBudget methodology, although it is an important bioeroder in the western Atlantic (Enochs et al., 2015). We estimated the rate of bioerosion by *P. lampa* at the CREMP stations using the generalized sponge bioerosion rate of 2.31 kg m^{-2} y^{-1} provided in the ReefBudget v1 methodology (Perry et al., 2012), which is based on data from Rose and Risk (1985) and Scoffin et al. (1980).

Because we did not have bioeroding sponge data for all years included in our study, we used site-level mean (±SE) sponge bioerosion rates across years in our estimates of total bioerosion for each year at each CREMP site. This approach did not allow us to evaluate potential changes in sponge bioerosion at the sites through time; however, because we did not detect any significant

temporal variability in sponge bioerosion from 2005–2009 (LME_{year}: $F_{4,546}$=1.03, p=0.39), we assume that the means are generally representative of the contribution of sponge bioerosion at each site throughout the study period. At two sites, Sand Key Shallow and Rock Key Shallow, all bioeroding sponges were combined into "*Cliona* sp." in the 2009 surveys, so only the data from 2005–2008 were used to calculate the site-level means for those two sites. Additionally, sponge surveys were not conducted at the six patch reef sites (two each in the Upper, Middle, and Lower Keys) added to the CREMP surveys in 2009 (Two Patches, Burr Fish, Rawa, Thor, Wonderland, Red Dun). We estimated sponge bioerosion at those sites using the 2005–2009 mean (±SE) from the other patch reefs in each respective region.

To summarize, our estimates of sponge bioerosion necessarily deviated from the ReefBudget methodology (Perry & Lange, 2019) in several ways (Table S2): 1) *Siphonodictyon* spp. sponges were not included because they were not recorded in the CREMP surveys; 2) our sponge bioerosion estimates do include *P. lampa*, which is not included in ReefBudget; and 3) we do not include temporal variability in sponge bioerosion in our budgets. Because of these deviations, our sponge bioerosion estimates are likely somewhat different from those that would be obtained using the standard ReefBudget methodology; however, because endolithic sponge bioerosion is a relatively minor contributor to total bioerosion in most western Atlantic locations (Perry et al., 2014; Perry et al., 2013), the uncertainties associated with our estimates of sponge bioerosion is likely not a significant source of uncertainty in our carbonate budgets as a whole.

Microbioerosion

To estimate microbioerosion, we first calculated the proportion of available, consolidated substrate by subtracting the proportion of "unconsolidated substrate" (i.e., sand, unconsolidated rubble, etc.) recorded in the CREMP data from one (i.e., consolidated substrate = 1 – unconsolidated substrate). Prior to 2010, the CREMP surveys did not distinguish between consolidated and unconsolidated substrate. Although there was significant variability in the unconsolidated substrate from 2010–2019 (LME_{year}: $F_{9,1765}$=11.38, p<0.001), with a significantly higher proportion of unconsolidated substrate from 2015–2018 (p<0.05), the range in the proportion of unconsolidated substrate among years only varied by ~3% (i.e., from 0.014 to 0.044). Additionally, like crustose coralline algae, unconsolidated versus consolidated substrates can be difficult to distinguish in underwater imagery, so there is inherent uncertainty in these estimates from year-to-year. We, therefore, make the simplifying assumption that the proportion of available, consolidated substrate did not vary significantly at a given site through time and calculate the mean (±SE) proportion of available substrate at each site using the data from 2010–2019. We then estimated mean (±SE) microbioerosion by multiplying the mean (±SE) proportion of available substrate at each site by the mean global reef microbioerosion rate of 0.24 kg $CaCO_3$ m^{-2} y^{-1} (Chazottes, Campion-Alsumard, & Peyrot-Clausade, 1995; Chazottes, Le Campion-Alsumard, Peyrot-Clausade, & Cuet, 2002; Tribollet & Golubic, 2005) suggested in the ReefBudget v2 methodology (Perry & Lange, 2019).

Parrotfish Bioerosion

CREMP does not conduct fish surveys as part of their annual reef monitoring, so to obtain data on the abundance of parrotfishes we instead relied on a regional dataset of fishery-independent reef fish surveys using the Reef Visual Census (RVC) protocol (Brandt et al., 2009; Smith et al., 2011). The RVC surveys have been conducted throughout the Florida Keys since 1999 by scientists from the National Oceanic and Atmospheric Administration (NOAA), FWRI, the National Park Service, and University of Miami's Rosenstiel School of Marine and Atmospheric Science. They are now part of NOAA's National Coral Reef Monitoring Program (NCRMP). During each year of the surveys (RVC: annually 1999 – 2012; NCRMP RVC: biannually 2014-2018), hundreds of survey sites throughout the region are selected using a habitat- and depth-stratified random design. At each primary survey location, two divers conduct simultaneous, timed stationary fish surveys (20–60 m apart), recording the number and mean fork length of all fish species observed within a 7.5 m radius (Brandt et al., 2009; Smith et al., 2011), based on the method developed by Bohnsack and Bannerot (1986). We note that the primary goal of the RVC surveys is to assess the populations of commercially important fishes (e.g., groupers, snappers; Brandt et al., 2009; Smith et al., 2011). For non-target species recorded in the RVC surveys, like parrotfishes, lengths are often recorded as minimums, maximums, and modes for a site, which are fit with a triangular distribution to produce the "length" values in the dataset (Grove, Blondeau, & Ault, 2021).

We downloaded all available RVC data from the Florida Keys and Dry Tortugas from https://grunt.sefsc.noaa.gov/rvc_analysis20/. We used the buffer and spatial join tools in ArcGIS (v.7.1.0) to extract all RVC surveys in each year that were within a 10 km radius of each CREMP site. We also explored using 1 km, 2 km, and 5 km radii; however, we found that the smaller selection radii resulted in a high proportion of CREMP sites for which no data were available in multiple years. This was particularly problematic for CREMP patch reef sites because ~50% fewer patch-reef habitats are included in the RVC surveys each year than fore-reef habitats. We settled on the 10 km selection radius, as we found that this area generally (83% of all years and sites) resulted in at least six, independent RVC surveys for each CREMP site in each year, analogous to the six, belt transect surveys recommended in the ReefBudget protocol (Perry & Lange, 2019; Perry et al., 2012).

We used the designations of habitat type ("stratum"), characteristics ("habitat class"), and depth of each primary survey location in the spatially aligned RVC dataset (Brandt et al., 2009) to further subset the data so that only reef habitats most similar to each of the CREMP sites were included. For the CREMP patch-reef sites in the Keys subregion, we selected sites from the spatially aligned RVC dataset with a patch-reef habitat designation. For the CREMP offshore shallow sites in the Keys subregion, we first subset the RVC data to only include shallow (<6 m water depth) and mid-depth (6–18 m water depth) fore-reef habitats. The mean water depth of the offshore shallow Keys CREMP sites is 4.8 m, with a range of 1.8–7.3 m. We, therefore, further subset the fore-reef RVC data to only include surveys with a water depth of 7.5 m or less for the CREMP offshore shallow sites. The offshore deep Keys CREMP sites have a mean depth of 14.5 m with a range of 10.7–16.5 m. To select data for those sites, we subset the mid-depth

fore-reef RVC habitats to only include surveys collected between 10.5 and 16.5 m water depth. We also removed all sites in the RVC datasets characterized by dominance of low-relief rubble. Habitats in the RVC data were classified somewhat differently in the Dry Tortugas subregion. To assign habitats for the CREMP sites there, M.A. Colella and S.A. Kupfner Johnson identified the RVC habitats most similar to each CREMP site based on their field observations of the CREMP sites and descriptions of the RVC habitats (Brandt et al., 2009; Smith et al., 2011). Based on their assessments, one CREMP site (Bird Key Reef) was assigned RVC data with the high-relief spur-and-groove habitat classification, seven pinnacle or patch reef sites (The Maze, Davis Rock, Texas Rock, Temptation Rock, Mayer's Peak, and Black Coral Rock) were assigned RVC data from the high-relief continuous reef habitat classification, and the remaining four sites (White Shoal, Palmata Patch, Prolifera Patch, and Loggerhead Patch) were assigned RVC data from the low-relief continuous reef habitat classification. We acknowledge that the unavailability of co-located parrotfish data, and our use instead of the stratified random RVC fish surveys, potentially introduces significant uncertainty into the parrotfish erosion terms of our carbonate budget models; however, by carefully selecting data from habitats that were as similar as possible to the CREMP sites, we assume that these data generally reflect the spatial distribution and habitat preferences of parrotfishes in the region.

We used the observations of parrotfishes from the RVC dataset to estimate parrotfish bioerosion based on the ReefBudget v2 methodology (Perry & Lange, 2019). First, data were sorted into 10-cm length bins for each of the bioeroding parrotfish species: *Sparisoma viride*, *Sp. aurofrenatum*, *Sp. rubripinne*, *Sp. chrysopterum*, *Scarus vetula*, *Sc. taeniopterus*, *Sc. iseri*, *Sc. guacamaia*, and *Sc. coelestinus*. We note that although Adam et al. (2018) concluded that *Sp. aurofrenatum*, *Sp. rubripinne*, *Sp. chrysopterum*, and *Sc. coeruleus* do not contribute significantly to bioerosion in most cases, we follow the ReefBudget v2 methodology (Perry & Lange, 2019) and include those taxa in our calculations. Those fishes were rare in our dataset and ultimately only contributed ~1.5% of the total estimated parrotfish bioerosion in our study, so their inclusion does not significantly impact our overall results. We do not include observations of *Sparisoma* or *Scarus* spp. that were not identified to the species level. For *Scarus* spp. this resulted in the elimination of just 117 observations of adult fishes out of nearly 100,000 total observations and all "*Sparisoma* spp." were juveniles, which are not assigned bioerosion rates under the ReefBudget v2 methodology (Perry & Lange, 2019).

Bioerosion rates were calculated by multiplying the abundances of parrotfishes by the estimated species- and size-specific bioerosion rates provided in the ReefBudget v2 methodology (Perry & Lange, 2019); included in the USGS Software Release [Toth & Courtney, 2022]), assuming the standard mean coral density of 1.76 g cm^{-3}. Juvenile parrotfishes (<10 cm fork length) were assigned a bioerosion rate of 0. In 945 cases, parrotfishes in the RVC surveys had a recorded length of "-9", which indicated that a fish was observed, but its length wasn't recorded (Jennifer Herbig, FWRI, personal written communication). In those cases, we substituted the mean lengths observed for those species throughout the study period. Bioerosion rates for each species were summed across size classes for each (typically two) replicate survey at each primary RVC site and converted to kg m^{-2} y^{-1} by dividing by the survey area of 177 m^2. The data from each replicate survey were averaged to produce a single mean value for each primary RVC site.

Because the RVC surveys do not differentiate between initial and terminal phase parrotfishes, we calculated a minimum and maximum estimate of bioerosion by separately calculating bioerosion using the rates for initial phase and terminal phase individuals and then used the mean of those estimates as the mean bioerosion rate for each primary RVC site. Those data were summarized to estimate the mean (±SE) parrotfish bioerosion at each CREMP site for each year of the RVC surveys.

We ultimately used 8-year running means of the dataset to derive the final estimates of parrotfish bioerosion for each CREMP site during each year in our study. We made this analytical choice for two reasons. First, annual RVC survey data were available from the Florida Keys subregions from 1999–2012 and then every other year thereafter. In the Dry Tortugas, RVC surveys were conducted 1999 and 2000 and then every other year starting in 2004. As a result, parrotfish data were not available for the first three years of the CREMP surveys (1996–1998) and were only available biannually in later years of the study. The use of a running mean of parrotfish bioerosion allowed us to fill those data gaps and estimate total bioerosion for each year of the CREMP surveys. Second, as is common with fish survey data, there was high variability in the observations of parrotfishes among primary sites in the RVC dataset. This meant that sites with anomalously low or high abundances of parrotfishes could have a significant impact on the year-to-year estimates of parrotfish bioerosion at the CREMP sites. Whereas such variability may reflect real changes in local parrotfish populations, because the RVC surveys were not actually co-located with the CREMP sites, it is not possible to determine whether data extremes are realistic for our specific study sites. The running means account for this source of uncertainty by smoothing the parrotfish bioerosion data to provide a representation of more broad-scale spatial and temporal variability at the CREMP sites.

Reef-accretion potential

We converted net carbonate production into estimates of reef-accretion potential (cf. Perry et al., 2018), following the method described by Kinsey (1985), in which

$$\text{Reef-accretion potential} = \frac{Net\ carbonate\ production}{\rho(1-porosity)}$$

We followed Kinsey (1985) and used a $CaCO_3$ density, ρ, of 2.9 g cm^{-3}. We calculated framework porosity from a previously published dataset of Holocene (~11,700 years ago to present) reef cores collected from the Keys and Dry Tortugas subregions (170 recovered intervals in 43 cores; Toth, Kuffner, & Stathakopoulos, 2018). That dataset had a mean regional framework porosity of 0.63 (±0.02 SE), which is similar to estimates from other locations in the western Atlantic (e.g., Hubbard, Miller, & Scaturo, 1990; Kinsey, 1985).

Reef porosity can vary based on framework composition. Indeed, the porosity of *A. palmata* framework in the Florida Keys of 0.72 (±0.04) was significantly higher than that of massive facies at 0.59 (±0.02; Kruskal-Wallis test: X^2=9.15, p=0.01; *A. palmata* vs. massive framework p=0.008), with intervals of mixed framework being intermediate to the two at 0.61 (±0.06). Although the porosity of massive coral framework may be more closely analogous to contemporary reef assemblages in the Florida Keys, that estimate is within uncertainty of the

overall reef-framework porosity value used in this study. We, therefore, make the simplifying assumption that the regional mean porosity of 0.63 (±0.02) provides a reasonable approximation for all reefs evaluated in this study, while acknowledging that if our estimate of porosity is too high for modern reef frameworks, then we may be overestimating reef-accretion potential.

Supplementary Results & Discussion

Contributions of coral taxa to carbonate production

To evaluate the coral taxa responsible for variability in carbonate production in the Florida Keys, we first identified which taxa had mean carbonate production rates >0.05 kg $CaCO_3$ m^{-2} y^{-1} in any year in our study. The seven coral taxa that met that criterion were: *Orbicella* spp., *Montastraea cavernosa*, *Siderastrea siderea*, *Porites astreoides*, *Colpophyllia natans*, *Acropora palmata*, and *A. cervicornis* (Fig. S11). Only three of those taxa—*Orbicella* spp., *A. palmata*, and *S. siderea*—experienced consistent changes in coral cover and their relative contribution to carbonate production over time. We present the trends for those taxa across the 46 CREMP sites included in the statistical analyses in the main text and describe the analysis of the other four taxa below. We evaluate the same pairwise temporal contrasts, based on known regional disturbance events and for 1996 vs. 2019, as in the main text (1996 vs. 1999, 1999 vs. 2000, 2003 vs. 2006, 2009 vs. 2010, and 2017 vs. 2018); however, in cases where there was a significant fixed effect of year, but there were no statistically detectable changes for any of those pairwise comparisons, we describe all significant contrasts between years. Trends in all seven taxa were similar when the dataset was analyzed using the subset of 32 CREMP sites that were surveyed annually from 1996–2019 (see section on "Carbonate budgets of sites established in 1996"), suggesting that the addition of sites over time did not bias the results.

Although there were not consistent increases in the cover of *P. astreoides* over time (LME_{year}: $F_{23,887}=2.83$, $p<0.001$; *P. astreoides* cover was only significantly higher in 2002 & 2003 vs. 2004 & 2017; Tukey tests: $p<0.05$), its contribution to gross carbonate production increased significantly from 1996 to 2019 (Fig. S11c; LME_{year}: $F_{23,887}=2.54$, $p<0.001$; Tukey test: $p>0.001$), putatively as a result of the decreasing contribution of other, reef-building taxa. Although there was no statistically detectable change in the contribution of *P. astreoides* to carbonate production following the 1997–1998, 2014, and 2015 bleaching events or the hurricane impacts in 1999 and 2017 (Tukey tests 1996 vs. 1999, 1999 vs. 2000, 2013 vs. 2016, and 2017 vs. 2018: $p>0.05$), there was a significant increase in the relative contribution of *P. astreoides* to carbonate production following the hurricanes in 2004 and 2005 and the coral bleaching event in 2005 (Tukey test 2003 vs. 2006: $p<0.05$). *Porites astreoides* cover and its contribution to carbonate production were also highest in offshore shallow habitats ($LME_{habitat}$: $F_{2,40}=5.19$, $p<0.01$ and $F_{2,40}=7.19$, $p<0.01$, respectively; Tukey test: $p<0.05$ for offshore shallow vs. offshore deep for both cover and percent carbonate production; $p<0.01$ for offshore shallow vs. patch reefs for percent carbonate production). There were no significant effects of subregion on *P. astreoides* cover ($LME_{subregion}$: $F_{3,40}=1.20$, $p=0.32$) or its contribution to gross carbonate production ($LME_{subregion}$: $F_{3,40}=0.25$, $p=0.86$).

The cover of *M. cavernosa* varied significantly over time (Fig. S11a; LME_{year}: $F_{23,887}=2.91$, $p<0.001$), with significantly lower cover in 2019 compared with 1996 (Tukey test: $p<0.01$); however, there were no statistically detectable changes associated with any of the identified thermal stress events or hurricanes from 1996–2019 (Tukey test 1996 vs. 1999, 1999 vs. 2000, 2003 vs. 2006, 2009 vs. 2010, 2013 vs. 2016, and 2017 vs. 2018: $p>0.05$). There was also no significant difference in the percent contribution of *M. cavernosa* to gross carbonate production over time (LME_{year}: $F_{23,887}=0.86$, $p=0.65$). Similarly, whereas the cover of *M. cavernosa* was significantly higher in the Lower Keys subregion compared with the Dry Tortugas and the Upper Keys ($LME_{subregion}$: $F_{3,40}=4.50$, $p=0.01$; Tukey test: $p<0.05$), there was no statistically detectable difference in its contribution to gross carbonate production by subregion ($LME_{subregion}$: $F_{3,40}=2.63$, $p=0.06$). Cover of *M. cavernosa* and its contribution to gross carbonate production were both significantly higher in patch-reef habitats compared with offshore habitats ($LME_{habitat}$: $F_{2,40}=7.92$, $p=0.001$ and $F_{2,40}=6.99$, $p=0.003$, respectively; Tukey tests: $p<0.005$ and $p<0.05$, respectively).

Although there was a significant effect of year on the cover of *C. natans* (LME_{year}: $F_{23,887}=1.96$, $p<0.005$), there were no statistically detectable changes in its cover between 1996 and 2019 or in response to any of the identified disturbance events (Tukey test: $p>0.05$); however, there was a significant decline in the cover of *C. natans* from 2017–2019 (Fig. S11a; Tukey test 2016 vs. 2019: $p<0.05$): a trend most likely driven by the extremely high vulnerability of this taxon to SCTLD (Gintert et al., 2019; Muller, Sartor, Alcaraz, & van Woesik, 2020); however, we did not directly evaluate the impacts of SCTLD on *C. natans* in this study. There were also significant differences in *C. natans* cover for 2007, 2009, 2010, 2012, and 2014 vs. 2019 and 2009 vs. 2018 (Tukey tests: $p<0.05$). Temporal changes in carbonate production over this period were only statistically detectable for 2007 vs. 2018 and 2019 (LME_{year}: $F_{23,887}=1.62$, $p=0.03$; Tukey test: $p<0.05$). The temporal changes were most pronounced in patch-reef habitats, where *C. natans* had the highest cover ($LME_{habitat}$: $F_{2,40}=6.12$, $p<0.005$; Tukey test: $p<0.005$) and the highest contribution to gross carbonate production ($LME_{habitat}$: $F_{2,40}=10.02$, $p<0.001$; Tukey test: $p<0.005$). Among subregions, the cover of *C. natans* was significantly higher in the Lower Keys compared with the Dry Tortugas ($LME_{subregion}$: $F_{3,40}=3.64$, $p=0.02$: Tukey test: $p<0.05$), but there was no statistically detectable difference in its contribution to gross carbonate production among subregions ($LME_{subregion}$: $F_{3,40}=2.88$, $p=0.05$; Tukey tests: $p>0.05$ for all pairwise comparisons).

The cover of *A. cervicornis* decreased significantly over time (Fig. S11a; LME_{year}: $F_{23,887}=1.60$, $p=0.04$; Tukey test 1996 vs. 2019: $p<0.005$), but there were no statistically detectable changes in response to any of the thermal stress events or hurricane impacts (Tukey tests: $p>0.05$). Additionally, there was no significant change in the contribution of *A. cervicornis* to gross carbonate production over time (LME_{year}: $F_{23,887}=1.34$, $p=0.13$). There was also no effect of habitat or subregion on the cover of *A. cervicornis* ($LME_{habitat}$: $F_{2,40}=1.03$, $p=0.37$; $LME_{subregion}$: $F_{3,40}=2.26$, $p=0.10$) or its contribution to gross carbonate production ($LME_{habitat}$: $F_{2,40}=0.92$, $p=0.41$; $LME_{subregion}$: $F_{3,40}=2.66$, $p=0.06$).

Contributions of bioeroding groups to carbonate budgets

Together parrotfishes and microbioeroders accounted for >96% of total bioerosion across all sites and years, with average rates of 0.26 (±0.01) and 0.23 (± <0.01), respectively (Table S7). The most significant parrotfish bioeroder was *Sparisoma viride*, accounting for two-thirds of all parrotfish bioerosion. Of the remaining bioeroding parrotfishes, *Scarus guacamaia* contributed 7%, *Scarus coeruleus* contributed 6%, *Scarus vetula* contributed 5%, and all others contributed <2%. Estimated bioerosion varied little through time at most sites (Figs. S1–S4; range: 0.61 to 0.39 on average): a trend that generally reflects the estimated changes in parrotfish bioerosion (Fig. S7 and S8). Because we were unable to fully constrain temporal variability in bioerosion in this study, however, we only statistically evaluated spatial variability in bioerosion and its components. Total bioerosion and parrotfish bioerosion were both significantly higher in offshore shallow habitats compared with offshore deep and patch-reef habitats ($LME_{habitat}$: $F_{2,40}$=26.87, p<0.001 and $F_{2,40}$=3.28, p=0.03, respectively; Tukey tests: p<0.001). In contrast, sponge bioerosion was higher in patch reefs than in the offshore habitats ($LME_{habitat}$: $F_{2,40}$=3.62, p=0.04; Tukey tests: p<0.05). Differences in microbioerosion among habitats were not statistically detectable ($LME_{habitat}$: $F_{2,40}$=3.11, p=0.06). Among subregions, total bioerosion was significantly lower in the Middle Keys subregion compared with the Upper Keys (Fig. S6c; $LME_{subregion}$: $F_{3,40}$=3.01, p=0.04; Tukey test: p<0.05): a trend likely driven by parrotfish bioerosion and *D. antillarum* bioerosion being lowest in the Middle Keys (Fig. S8); however, only parrotfish had a statistically detectable difference between the Middle and Upper Keys ($LME_{subregion}$: $F_{3,40}$=3.28, p=0.03; Tukey test: p<0.05). Bioerosion by *D. antillarum* was statistically higher in the Dry Tortugas compared with all the Keys subregions ($LME_{subregion}$: $F_{3,40}$=3.52, p=0.02; Tukey test: p<0.05) and the difference in microbioerosion among subregions was not statistically detectable ($LME_{subregion}$: $F_{3,40}$=0.26, p=0.85). Sponge bioerosion also varied among subregions, with significantly lower bioerosion in the Upper Keys compared with the Lower Keys and in the Dry Tortugas compared with both the Lower and Middle Keys ($F_{3,40}$=19.58, p<0.001; Tukey tests: p<0.001).

In our study, total bioerosion averaged 0.51 (±0.01; range: 1.95 to 0.23) kg $CaCO_3$ m^{-2} y^{-1} across all sites and years, which is lower than what has been reported for most other locations in the western Atlantic (Perry et al., 2018; Perry et al., 2014; but see Molina-Hernández, González-Barrios, Perry, & Álvarez-Filip, 2020). Although our estimates of bioerosion by sponges, urchins, and microbioeroders were typical for the region (Table S7), parrotfish bioerosion was more than 50% lower in our study than most other carbonate-budget studies from south Florida, and more than five-times lower than the average parrotfish bioerosion rate for the western Atlantic (Table S7; Kuffner et al., 2019; Manzello et al., 2018; Perry et al., 2018). To evaluate the likely drivers of the relatively low parrotfish bioerosion rates calculated in our study, we compared parrotfish densities (Table S8) and size frequency distributions (Fig. S13) from our dataset to other studies from south Florida. We also assessed the impact of methodological differences in calculating parrotfish bioerosion between this study and previous carbonate-budget studies from the region.

We calculated a mean parrotfish density of 8.65 individuals per 100 m^2 (± 0.23) throughout the Florida Keys based on RVC habitats similar to the CREMP sites. Although that value is similar to total densities of parrotfishes observed in other studies in the region (e.g., 9.72 ± 4.39 at Hen & Chickens Reef [Kuffner et al., 2019]; see also Adam, Kelley, Ruttenberg, & Burkepile [2015]), the density of the most important bioeroding species, *Sp. viride* (Perry et al., 2014), was only 0.57 individuals per 100 m^2 (± 0.02) in our study compared with ~1.4 to 4 individuals per 100 m^2 in several other recent studies in the region (Adam et al., 2015; Burkepile, 2012; Kuffner et al., 2019). Indeed, only ~7% of parrotfishes in our dataset were *Sp. viride*, and the most abundant parrotfish, accounting for ~63% of all parrotfishes observed, was *S. iseri* (Table S8; Fig. S13). By comparison, *S. iseri* only accounted for 21% of the parrotfish observations in a recent study at Hen & Chickens patch reef in the Upper Keys (Kuffner et al., 2019). Without *S. iseri*, average density of parrotfishes in our study was just 2.89 individuals per 100 m^2 (± 0.16). We note that our estimated densities of parrotfishes are similar to those calculated using the entire RVC dataset for the years 1999–2008 (Smith et al., 2011).

A recent study that evaluated populations of parrotfishes throughout the western Atlantic (Shantz, Ladd, & Burkepile, 2020), suggested that adult parrotfishes (10+ cm fork length in our study) are more abundant in Florida than in most other locations in the Caribbean. Our data from the RVC surveys largely support this conclusion, with fork lengths for most bioeroding species averaging at least 20 cm (Fig. S13). One important exception, however, is *Sp. viride*. Although the mean fork length of *Sp. viride* in our study was 16.36 cm (±0.10), one-third of the individuals observed in the RVC surveys were less than 10 cm in length (Fig. S13). Because juvenile parrotfishes are not considered in the bioerosion estimates in our study (following ReefBudget v2; Perry & Lange [2019]), many of the *Sp. viride* observations were not included in our bioerosion estimates. Smaller individuals were also common for *S. iseri*, *S. taeniopterus*, and *Sp. aurofrenatum*, but those parrotfishes typically have a smaller maximum size than the other bioeroding parrotfish species (www.fishbase.org; Perry & Lange, 2019).

The lower densities of parrotfishes overall, and the low density of *Sp. viride*, in particular, combined with the high abundance of juvenile *Sp. viride* individuals, explains the relatively low parrotfish bioerosion rates in our study. The cause of these differences is somewhat unclear, but one possibility could be that the RVC surveys were designed with the primary goal of assessing populations of commercially important fishes (e.g., groupers, snappers; Brandt et al., 2009; Smith et al., 2011). As a non-target group, parrotfishes could be undercounted in the RVC surveys; however, Brandt et al. (2009) noted that this bias is unlikely given that many non-target groups, including parrotfishes, are more abundant than target species in south Florida and should, therefore, be easier, not harder to detect. In a direct comparison of the RVC method used in this study to the belt-transect method advocated by the ReefBudget methodology (Perry & Lange, 2019; Perry et al., 2012), Sale (1997) concluded that the RVC method provides the most accurate method for quantifying fish populations; however, absolute densities of fishes nonetheless may be somewhat underestimated (J. Bohnsack, personal written communication). Conversely, a recent modeling study found that the belt-transect method, can substantially overestimate mobile species (Ward-Paige, Mills Flemming, & Lotze, 2010). That bias was most significant for highly mobile taxa like sharks, but Ward-Paige et al. (2010) suggested that instantaneous stationary

surveys, should also be more accurate at assessing parrotfish populations. Although no fish-survey method can be considered truly "accurate" (Bohnsack & Bannerot, 1986; Sale, 1997; Ward-Paige et al., 2010), we conclude that the RVC data used in our study provide a realistic estimate of parrotfish densities in south Florida. It is also possible that utilizing RVC data to approximate parrotfish densities at the CREMP sites, rather than being able to use co-located surveys, resulted in the somewhat lower parrotfish densities and estimates of bioerosion in our study; however, because we only include RVC data from the same general area and habitats as the CREMP sites, those data should reflect the general patterns of parrotfish abundance on those reefs.

A final factor contributing to the relatively low parrotfish bioerosion rates in our study is that previous studies from the region used an earlier version of the ReefBudget methodology (v1; Perry et al., 2012), which included juvenile parrotfishes in the bioerosion estimates, whereas our study did not (following the recommendation of ReefBudget v2 [Perry & Lange, 2019]). We estimated the impact of this difference by re-calculating our budgets using the ReefBudget v1 methodology (Perry et al., 2012; see Toth & Courtney, 2022). Including juvenile parrotfishes increased our parrotfish bioerosion estimates to 0.45 (± 0.01) kg $CaCO_3$ m^{-2} y^{-1}, which is nearly double the rates estimated in our study (0.26 ± 0.01). Assuming that the analytical choice to exclude juvenile parrotfishes in new ReefBudget methodology is more accurate, this suggests that parrotfish bioerosion may have been over-estimated in previous studies. Nonetheless, even with the juvenile data, our data still produce parrotfish bioerosion rates more than 1.5 times lower than most other studies in the region (Table S7), suggesting that while this methodological difference accounts for some portion of the relatively low parrotfish bioerosion rates in our study, it cannot fully explain the discrepancy.

Although the relatively lower rates of parrotfish bioerosion are not entirely unrealistic (cf. Dry Tortugas in Table S7; Molina-Hernández et al., 2020), it is possible that bioerosion is somewhat underestimated and, therefore, net carbonate production is somewhat overestimated in our study. To illustrate how this would impact our estimated budgets, we recalculated the mean (±SE) percentage of the 32 sites established in 1996 that had net positive carbonate production over time, substituting the western Atlantic mean bioerosion rate calculated by Perry et al. (2018; 1.64 kg $CaCO_3$ m^{-2} y^{-1}) for our estimated bioerosion rate. We evaluated the trends in those data with a linear regression (model residuals conformed to the assumption of normality; Shapiro-Wilk tests: $p>0.05$). As in the analysis using the bioerosion rates derived in this study, the regressions based on the Perry et al. (2018) bioerosion rates suggest a significant decline in the percentage of sites with positive net carbonate budgets over time (LM: $F_{1,22}=46.88$, $p<0.001$); however, if parrotfish bioerosion is in fact more comparable to the average rate estimated by Perry et al (Perry et al., 2018), then only ~6% (2) of reefs in our study would have positive carbonate budgets at present (Fig. S14; versus 15% using our bioerosion rates).

<u>Carbonate budgets of sites established in 1996</u>

Here, we evaluate the possibility that the addition of new sites at various points during the study may have influenced trends in our carbonate budgets. To account for that possibility, we repeated the statistical analyses presented in the main text using only the 32 sites that were surveyed

annually during the entire study periods from 1996–2019. The overall results of these analyses were very similar to those for the full dataset. One notable exception was the apparent increase of ~1% in the cover of *Orbicella* spp. in 2009, which was primarily an artifact of the addition of six new patch-reef sites in the Keys subregions in that year; however, real increases in *Orbicella* spp. cover at two previously established patch reefs (Admiral and Jaap Reef; Fig. S2) also contributed to that trend, with the "real" increase in average *Orbicella* spp. cover being ~0.34%. The increase in average *Orbicella* spp. cover in our dataset drove apparent increases in both total average coral cover of ~0.8% and carbonate production of ~0.05 kg $CaCO_3$ m^{-2} y^{-1} in 2009. Because the patch reefs that were added in 2009 experienced similar declines in coral cover to previously established patch reefs following the 2010 cold event (Fig. S2), the small increase in *Orbicella* spp. cover in 2009 had little overall effect on the overall trends in our study.

Mean net carbonate production of the 32 reefs in the Florida Keys subregions that were surveyed annually from 1996–2019 was 0.51 (± 0.05 SE) kg $CaCO_3$ m^{-2} y^{-1} (range= -0.97 to 8.06 kg $CaCO_3$ m^{-2} y^{-1}). This equates to a mean reef-accretion potential of 0.47 (±0.04) mm y^{-1} (range= -0.89 to 7.44 mm y^{-1}). Region-wide net carbonate production and reef-accretion potential declined significantly over time (LME_{year}: $F_{23,713}$=5.30, p<0.001; Tukey test 1996 vs. 2019: p<0.001), with significant declines after the 1997–1998 bleaching event (Tukey test 1996 vs. 1999: p<0.001) and after the 2010 cold event (Tukey test 2009 vs. 2019: p<0.001). There were no statistically detectable changes associated with the hurricanes in 1999, 2004, 2005, and 2017 or the thermal stress event in 2005 (Tukey tests: p>0.05). We did not evaluate the impact of SCTLD using the reduced dataset. Net carbonate production and reef-accretion potential were significantly higher in patch reefs compared with offshore habitats ($LME_{habitat}$: $F_{2,27}$=9.78, p<0.001; Tukey test: p<0.005) and were higher in the Lower Keys than in the Middle and Upper Keys subregions, but that trend was not statistically detectable ($LME_{subregion}$: $F_{2,27}$=3.18, p=0.06). Note that statistical results for these net carbonate production and reef-accretion potential are identical because mean reef-accretion potential rates are estimated by multiplying net carbonate production by ~0.92 (see Toth & Courtney, 2022). The predicted regional threshold of coral cover for maintaining net positive carbonate production on average when the LMEs were run with the subset of 32 sites was similar to that of the full dataset, 7% vs. 6%, respectively (patch reefs: 4–5%; offshore shallow: 10%; offshore deep: 5–6%).

Trends in the cover of the top carbonate-producing coral taxa and their contributions to carbonate production were also very similar when only the original 32 sites were analyzed. Those results are summarized in the following paragraphs. We highlight where there were minor deviations from the results based on the full dataset.

The cover of *A. palmata* and its percent contribution to carbonate production both declined significantly across the 32 sites (LME_{year}: $F_{23,713}$=5.55, p<0.001 and LME_{year}: $F_{23,713}$=2.66, p<0.001, respectively; Tukey test 1996 vs. 2019: p<0.01 for *A. palmata* cover; there was no statistically detectable difference in its contribution to carbonate production between 1996 and 2019; Tukey test: p>0.05). The most significant declines in both parameters occurred after the 1997–1998 bleaching event (Tukey tests 1996 vs. 1999: p<0.001), and there were no statistically detectable changes associated with any of the subsequent disturbance events (Tukey test:

p>0.05). The cover of *A. palmata* was significantly higher in offshore shallow habitats compared with offshore deep and patch-reef habitats ($LME_{habitat}$: $F_{2,27}$=6.15, p<0.01; Tukey test: p<0.05), and its contribution to carbonate production was likewise higher there ($LME_{habitat}$: $F_{2,27}$=6.30, p<0.01; Tukey test: p<0.05). There was no significant difference in *A. palmata* cover or its contribution to carbonate production among subregions ($LME_{subregion}$: $F_{2,27}$=0.64, p=0.54 and $F_{2,27}$=0.70, p=0.51, respectively).

The cover of *Orbicella* spp. declined significantly over time, with the most significant declines following the 2010 cold event (LME_{year}: $F_{23,713}$=2.38, p<0.001; Tukey test 2009–2010: p<0.001). The impacts of thermal stress events and hurricanes were not statistically detectable (Tukey test: p>0.05). The percent contribution of *Orbicella* spp. to carbonate production also declined over time (LME_{year}: $F_{23,713}$=1.83, p=0.01), and although none of the disturbance events had statistically detectable impacts (Tukey test: p>0.05), the fixed effects of years in the LME model indicate that this change occurred following the 2010 cold event (LME_{year} fixed effects of each year 2010–2019: p<0.05). There was no significant effect of habitat or subregion on the cover of *Orbicella* spp. ($LME_{habitat}$: $F_{2,27}$=2.08, p=0.14; $LME_{subregion}$: $F_{2,27}$=0.75, p=0.48) or its contribution to carbonate production ($LME_{habitat}$: $F_{2,27}$=0.30, p=0.74; $LME_{subregion}$: $F_{2,27}$=1.34, p=0.28).

The cover of *S. siderea* increased significantly over time (LME_{year}: $F_{23,713}$=2.07, p<0.005); however, there were no statistically detectable changes associated with any identified disturbance events or between 1996 and 2019 (Tukey tests: p>0.05). The percent contribution of *S. siderea* to carbonate production did increase significantly between 1996 and 2019 (LME_{year}: $F_{23,713}$=3.35, p<0.001; Tukey test: p<0.001) and following the hurricanes in 2004 and 2005 and the coral bleaching event in 2005 (Tukey test 2003 vs. 2006: p<0.05). None of the other disturbance events had statistically detectable impacts on the contribution of *S. siderea* to carbonate production (Tukey tests: p>0.05). Cover of *S. siderea* was significantly higher in patch reefs than in offshore habitats ($LME_{habitat}$: $F_{2,27}$=16.44, p<0.001; Tukey tests: p<0.001), but there was no difference in cover among the Keys subregions ($LME_{subregion}$: $F_{2,27}$=3.39, p=0.05; Tukey tests: p>0.05). The contribution of *S. siderea* to carbonate production was significantly lower in offshore shallow habitats compared with offshore deep habitats ($LME_{habitat}$: $F_{2,27}$=6.00, p<0.01; Tukey test: p<0.01), and significantly higher in the Middle Keys compared with the Lower and Upper Keys subregions ($LME_{subregion}$: $F_{2,27}$=6.95, p<0.005; Tukey tests: p<0.05). The difference among the Keys subregions was not statistically detectable in the full dataset.

The cover of *P. astreoides* was higher in some years, but it did not change consistently over time (LME_{year}: $F_{23,713}$=2.44, p<0.001; Tukey tests 2002 and 2003 vs. 2007: p<0.05). The contribution of *P. astreoides* to gross carbonate production did increase significantly over time (LME_{year}: $F_{23,713}$=2.41, p<0.001; Tukey test 1996 vs. 2019: p<0.001), but not in response to any of the disturbance events (Tukey tests: p>0.05). Its cover was also significantly higher in offshore shallow compared with offshore deep habitats ($LME_{habitat}$: $F_{2,27}$=3.52, p=0.04; Tukey test: p<0.05), but differences among habitats in its contribution to carbonate production were not statistically detectable ($F_{2,27}$=3.14, p=0.06). There were no significant effects of subregion on *P.*

astreoides cover ($LME_{subregion}$: $F_{2,27}$=1.54, p=0.23) or its contribution to gross carbonate production ($LME_{subregion}$: $F_{2,27}$=0.26, p=0.77).

The cover of *M. cavernosa* varied significantly over time (LME_{year}: $F_{23,713}$=2.40, p<0.001), but not in response to any of the identified disturbance events (Tukey tests: p>0.05). There was also no significant difference in the percent contribution of *M. cavernosa* to gross carbonate production over time (LME_{year}: $F_{23,713}$=0.90, p=0.60). Cover of *M. cavernosa* and its contribution to gross carbonate production were both significantly higher in patch reef habitats than in the offshore habitats ($LME_{habitat}$: $F_{2,27}$=8.89, p=0.001 and $F_{2,27}$=9.61, p<0.001, respectively; Tukey tests: p<0.01). Unlike in the analysis of the full dataset where we detected significantly higher cover of *M. cavernosa* in the Lower Keys, we found no difference in its cover in the reduced dataset ($LME_{subregion}$: $F_{2,27}$=2.33, p=0.12). As in the full dataset, there was no difference in the relative contribution of *M. cavernosa* to carbonate production by subregion ($LME_{subregion}$: $F_{2,27}$=1.88, p=0.17).

The cover of *C. natans* changed significantly over time (LME_{year}: $F_{23,713}$=1.69, p<0.05); however, none of the pairwise comparisons among between any years from 1996–2019 were statistically significant. The fixed effects of year in the LME model indicates that significant declines in *C. natans* cover beginning in 2016 (relative to 1996) were responsible for this result (LME_{year} fixed effects of each year 2016–2019 as well as 2000, 2006, and 2013: p<0.05). There was no statistically detectable change in the relative contribution of *C. natans* to gross carbonate production over time (LME_{year}: $F_{23,713}$=1.38, p=0.11). *C. natans* had the highest cover and the highest contribution to gross carbonate production in patch-reef habitats ($LME_{habitat}$: $F_{2,27}$=6.46, p<0.01 and $F_{2,27}$=6.26, p<0.01, respectively; Tukey tests: p<0.05). The cover of *C. natans* and its relative contribution to gross carbonate production were not significantly different among subregions in the analysis of the reduced dataset ($LME_{subregion}$: $F_{2,27}$=2.06, p=0.15 and $F_{2,27}$=2.07, p=0.15, respectively), whereas we found higher cover in the Lower Keys relative to the Dry Tortugas in the analysis of the full dataset.

In the reduced dataset, there were no statistically detectable differences in the cover of *A. cervicornis* (LME_{year}: $F_{23,713}$=1.54, p>0.05; $LME_{habitat}$: $F_{2,27}$=0.62, p=0.54; $LME_{subregion}$: $F_{2,27}$=0.60, p=0.56) or its contribution to gross carbonate production (LME_{year}: $F_{23,713}$=1.39, p=0.10; $LME_{habitat}$: $F_{2,27}$=0.02, p=0.97; $LME_{subregion}$: $F_{2,27}$=0.08, p=0.92) across space or time.

The trends in bioerosion based on the reduced dataset were very similar to those based on the full dataset. Bioerosion was significantly higher in offshore shallow habitats than in offshore deep and patch-reef habitats ($LME_{habitat}$: $F_{2,27}$=23.73, p<0.001; Tukey test: p<0.001) and significantly higher in the Upper Keys compared with the Middle Keys subregion ($LME_{subregion}$: $F_{2,27}$=5.06, p<0.05; Tukey test: p<0.05).

Supplementary Tables

Table S1. Description of the CREMP sites where we calculated carbonate budgets. Site names are listed with the CREMP site numbers in parentheses for reference. The latitude, longitude, subregion (DT=Dry Tortugas, LK=Lower Keys, MK=Middle Keys, and UK=Upper Keys), habitat type (OD=offshore deep reef, OS=offshore shallow reef, P=patch reef), approximate water depth (in meters [m]), and first year surveys were conducted are given for each site. The two LK back-county patch reef (BCP) sites in gray were not included in our study because they are in a different habitat than the primary FKRT. Although we calculated carbonate budgets for the three sites indicated with an asterisk, they were not included in the main text or the statistical analyses (their carbonate budgets are included in Table S6). Additionally, we did not include any of the Dry Tortugas sites or the six patch reef sites added to the Keys subregions in 2009 in our calculations of changes in the percentage of sites with positive carbonate budgets through time.

Site Name (#)	*Latitude*	*Longitude*	*Subregion*	*Habitat*	*Depth (m)*	*First Year*
Bird Key Reef (82)	24.6117	-82.8702	DT	OD	13.7	1999
Black Coral Rock (83)*	24.6993	-83.0022	DT	OD	21.6	1999
White Shoal (41)	24.6414	-82.8962	DT	P	8.8	1999
Mayer's Peak (45)	24.608	-82.944	DT	P	9.4	2004
Palmata Patch (42)*	24.6207	-82.8673	DT	P	2.7	2004
Prolifera Patch (43)*	24.6206	-82.8696	DT	P	2.1	2004
Temptation Rock (44)	24.6431	-82.9307	DT	P	7.0	2004
Loggerhead Patch (46)	24.6350	-82.9163	DT	P	2.1	2005
Davis Rock (28)	24.6871	-82.9072	DT	P	10.1	2009
Texas Rock (29)	24.6805	-82.8853	DT	P	10.4	2009
The Maze (27)	24.6090	-82.9496	DT	P	12.8	2009
Content Keys (18)	24.8221	-81.4889	LK	BCP	5.5	1996
Smith Shoal (39)	24.7197	-81.9195	LK	BCP	7.9	1996
Eastern Sambo Deep (78)	24.4884	-81.6659	LK	OD	14.6	1996
Looe Key Deep (77)	24.5421	-81.4153	LK	OD	12.5	1996
Rock Key Deep (80)	24.4532	-81.8568	LK	OD	12.8	1996
Sand Key Deep (81)	24.4517	-81.8798	LK	OD	10.7	1996
Western Sambo Deep (79)	24.4780	-81.7171	LK	OD	12.2	1996
Eastern Sambo Shallow (58)	24.4917	-81.6636	LK	OS	2.4	1996
Looe Key Shallow (57)	24.5453	-81.4079	LK	OS	7.3	1996
Rock Key Shallow (60)	24.4545	-81.8589	LK	OS	4.6	1996
Sand Key Shallow (61)	24.4520	-81.8775	LK	OS	6.4	1996
Western Sambo Shallow (59)	24.4796	-81.7176	LK	OS	4.3	1996
Cliff Green (38)	24.5036	-81.7677	LK	P	7.9	1996
Jaap Reef (40)	24.5857	-81.5826	LK	P	2.7	1996

Site Name (#)	***Latitude***	***Longitude***	***Subregion***	***Habitat***	***Depth (m)***	***First Year***
West Washer Women (36)	24.5475	-81.5866	LK	P	7.0	1996
Western Head (37)	24.4977	-81.8056	LK	P	10.4	1996
Red Dun Reef (26)	24.5209	-81.6621	LK	P	6.7	2009
Wonderland (25)	24.5603	-81.5021	LK	P	6.4	2009
Alligator Deep (74)	24.8452	-80.6209	MK	OD	11.3	1996
Sombrero Deep (76)	24.6223	-81.1120	MK	OD	15.2	1996
Tennessee Deep (75)	24.7527	-80.7578	MK	OD	13.4	1996
Alligator Shallow (54)	24.8457	-80.6240	MK	OS	4.9	1996
Sombrero Shallow (56)	24.6253	-81.1116	MK	OS	5.2	1996
Tennessee Shallow (55)	24.7450	-80.7812	MK	OS	6.4	1996
Dustan Rocks (35)	24.6895	-81.0302	MK	P	4.3	1996
West Turtle Shoal (34)	24.6993	-80.9669	MK	P	6.7	1996
Rawa Reef (23)	24.7176	-80.9357	MK	P	6.1	2009
Thor (24)	24.6715	-81.0506	MK	P	5.5	2009
Carysfort Deep (70)	25.2208	-80.2099	UK	OD	15.8	1996
Conch Deep (73)	24.9519	-80.4513	UK	OD	16.5	1996
Molasses Deep (72)	25.0072	-80.3756	UK	OD	13.4	1996
Carysfort Shallow (50)	25.2201	-80.2105	UK	OS	1.8	1996
Conch Shallow (53)	24.9553	-80.458	UK	OS	5.5	1996
Grecian Rocks (51)	25.1075	-80.3068	UK	OS	3.4	1996
Molasses Shallow (52)	25.0088	-80.3765	UK	OS	5.8	1996
Admiral (33)	25.0447	-80.3948	UK	P	1.8	1996
Porter Patch (32)	25.1032	-80.3243	UK	P	4.6	1996
Turtle (30)	25.2947	-80.2191	UK	P	4.0	1996
Burr Fish (22)	24.9992	-80.4669	UK	P	4.6	2009
Two Patches (21)	25.0219	-80.439	UK	P	2.4	2009

Table S2. Summary of the primary data sources used to estimate the components of our carbonate budgets and the necessary deviations that were made from the ReefBudget methodology.

Budget parameter	***Primary data description***	***Deviations from ReefBudget***
Gross carbonate production	Percent cover of coral taxa and coralline algae recorded at 2–4 permanent transects at each CREMP site from 1996–2019	• Area-normalized calcification rates that incorporate colony size and rugosity from Courtney et al. (2021) were used instead of ReefBudget calcification rate parameterizations because CREMP quantifies planar percent cover rather than three-dimensional surface area (ReefBudget).<br>• 2011–2019 site-level averages of crustose coralline algae cover were used to approximate cover for 1996–2010 when crustose coralline algae was not recorded.
Urchin bioerosion	Density of *D. antillarum* at 2–4 permanent transects at each CREMP site from 1996–2019	• The bioeroding *Echinometra lucunter*, *Echinometra viridis*, and *Eucidaris tribuloides* were not included in the CREMP surveys.<br>• CREMP does not record urchin test sizes so empirical size distributions from Chiappone et al. (2008) were used to estimate urchin bioerosion rates.
Sponge bioerosion	Surface area of *Cliothosa delitrix*, *Cliona varians*, *Cliona caribbaea*, and *Pione lampa* at 2–4 permanent transects at each CREMP sites from 2005–2009	• Site-level means (±SE) rather than annual sponge bioerosion data were used because the complete sponge surveys were only conducted by CREMP from 2005–2009.<br>• Mean (±SE) sponge bioerosion from other patch reefs in each subregion were used to estimate sponge bioerosion for the six Keys patch reefs added by CREMP in 2009, as sponges were not surveyed at those sites.<br>• *Siphonodictyon* spp. was not recorded in the CREMP surveys.<br>• *Pione lampa* was included in the CREMP surveys, but is not included in ReefBudget so the generalized sponge bioerosion rate of 2.31 kg m^{-2} y^{-1} (Perry et al., 2012) was used for this taxon.
Microbioerosion	Percent cover of available (consolidated substrate) at 2–4 permanent transects at each CREMP sites from 2010–2019	• Site-level means (±SE) rather than annual data on the cover of available substrate used because CREMP only distinguished consolidated and unconsolidated substrates beginning in 2010.
Parrotfish bioerosion	Reef visual census (RVC) data collected annually (1999–2012) or biannually (2014–2018) at 100s of stratified random survey locations throughout south Florida	• RVC surveys from similar habitats and depths within a 10 km radius of each CREMP site were used to approximate parrotfish abundances, as parrotfish were not surveyed at the CREMP sites.<br>• RVC data were not available in all years so we used eight-year running means of the parrotfish bioerosion data.<br>• RVC data are based on stationary fish surveys rather than belt transects suggested by ReefBudget.<br>• Parrotfish life phases are not recorded in the RVC surveys so parrotfish bioerosion was calculated based on the rates for both initial and terminal phase individuals and those data were averaged to estimate the mean parrotfish bioerosion for each RVC data point.

Table S3. Percent increases in coral cover of *Acropora palmata*, *Acropora cervicornis*, and *Orbicella* spp. in spur-and-groove top habitats (offshore shallow habitats in our study) at Looe Key, Sombrero, and Carysfort reefs based on the targets for Mission: Iconic Reefs (NOAA, 2021) used to model the impacts of restoration on reef-accretion potential.

	Looe Key Reef	***Sombrero Reef***	***Carysfort Reef***
Coral taxon	Phase 1		
A. palmata	8%	8%	10%
A. cervicornis	4%	4%	6%
Orbicella spp.	1%	1%	0.5%
Coral taxon	Phase2		
A. palmata	2%	2%	0%
A. cervicornis	2%	2%	0%
Orbicella spp.	1%	1%	1.5%

Table S4. Estimated changes in reef-accretion potential (mm y^{-1}) under various coral restoration scenarios compared with Holocene baselines (before 3000 years ago when regional reef accretion had ceased [Toth, Kuffner, Stathakopoulos, & Shinn, 2018]) and to historical (CREMP 1996, this study) and present-day rates (CREMP 2019, this study) for three sites included in this study that are targeted for restoration through NOAA's Mission: Iconic Reefs (M:IR) initiative (NOAA, 2021). The Phase 1 (10 years, 2030) and Phase 2 (20 years, 2040) M:IR scenarios are based on target increases in the cover of *Acropora palmata*, *Acropora cervicornis*, and *Orbicella* spp. for spur-and-groove top habitats at each site (Table S3). Generalized targets bracket those values for *Acropora* and *Orbicella* spp. and we also include a scenario for *Siderastrea siderea* based on the targets for *Orbicella* spp. Reef-accretion potential in bold are mean estimates and ranges based on the SEs of our carbonate budget models are provided in parentheses. Coloration of mean reef-accretion potential follows the dark blue to red color ramp used for net carbonate production values in Figure 1 (main text), where blue values represent positive reef-accretion potential, red and orange values represent negative reef-accretion potential, and yellow values represent reef-accretion potential that overlaps with 0 (i.e., neutral reef-accretion potential).

Restoration Scenario	***Looe Key Reef***	***Sombrero Reef***	***Carysfort Reef***
Holocene baseline	**5.43**<br>(4.1 to 6.6)	**5.08**<br>(1.4 to 13.2)	**3.42**<br>(1.5 to 5.2)
CREMP 1996	**2.20**<br>(-0.78 to 7.90)	**1.46**<br>(-0.34 to 3.70)	**0.51**<br>(-0.45 to 4.04)
CREMP 2019	**0.09**<br>(-1.16 to 1.75)	**-0.73**<br>(-1.23 to -0.01)	**-0.46**<br>(-0.63 to -0.25)
M:IR Phase 1	**3.34**<br>(1.31 to 5.97)	**2.52**<br>(1.25 to 4.20)	**3.84**<br>(2.65 to 5.33)
M:IR Phase 2	**4.73**<br>(2.41 to 7.73)	**3.91**<br>(2.34 to 5.96)	**4.17**<br>(2.94 to 5.72)
+5% *A. palmata*	**0.96**<br>(-0.57 to 2.96)	**0.14**<br>(-0.63 to 1.19)	**0.41**<br>(-0.04 to 0.95)
+10% *A. palmata*	**1.82**<br>(0.03 to 4.17)	**1.01**<br>(-0.04 to 2.40)	**1.27**<br>(0.56 to 2.16)
+15% *A. palmata*	**2.69**<br>(0.62 to 5.38)	**1.87**<br>(0.55 to 3.61)	**2.13**<br>(1.15 to 3.37)
+5% *A. cervicornis*	**2.14**<br>(0.50 to 4.29)	**1.32**<br>(0.43 to 2.52)	**1.59**<br>(1.03 to 2.28)
+10% *A. cervicornis*	**4.19**<br>(2.16 to 6.83)	**3.37**<br>(2.10 to 5.06)	**3.63**<br>(2.69 to 4.82)
+15% *A. cervicornis*	**6.23**<br>(3.83 to 9.37)	**5.42**<br>(3.76 to 7.60)	**5.68**<br>(4.36 to 7.36)
+1% *Orbicella* spp.	**0.31**<br>(-0.97 to 2.01)	**-0.50**<br>(-1.03 to 0.24)	**-0.24**<br>(-0.44 to 0.00)
+2% *Orbicella* spp.	**0.54**<br>(-0.77 to 2.67)	**-0.28**<br>(-0.84 to 0.50)	**-0.02**<br>(-0.24 to 0.26)
+5% *Orbicella* spp.	**1.20**<br>(-0.19 to 3.04)	**0.39**<br>(-0.26 to 1.27)	**0.65**<br>(0.34 to 1.03)
+1% *S. siderea*	**0.17**<br>(-1.09 to 1.84)	**-0.65**<br>(-1.16 to 0.08)	**-0.38**<br>(-0.56 to -0.17)
+2% *S. siderea*	**0.25**<br>(-1.03 to 1.93)	**-0.57**<br>(-1.10 to 0.17)	**-0.31**<br>(-0.50 to -0.08)
+5% *S. siderea*	**0.48**<br>(-0.83 to 2.20)	**-0.34**<br>(-0.90 to 0.43)	**-0.07**<br>(-0.30 to 0.19)

Table S5. Results of Mantel's tests used to evaluate spatial autocorrelation in the variables analyzed with LME models. The few variables that showed significant spatial autocorrelation are indicated with an asterisk; however, they all had extremely low correlation coefficients (Mantel r) suggesting that spatial autocorrelation is negligible in our dataset.

Variable	***Mantel r***	***p-value***
Net carbonate production (all 46 sites)	-0.03	0.99
Net carbonate production (32 sites surveyed 1996–2019)	0.02	0.06
Reef-accretion potential (all 46 sites)	-0.03	0.98
Reef-accretion potential (32 sites surveyed 1996–2019)	0.02	0.04*
Bioerosion (all 46 sites)	-0.03	1.00
Bioerosion (32 sites surveyed 1996–2019)	<0.01	0.41
A. palmata cover (all 46 sites)	-0.02	0.91
A. palmata cover (32 sites surveyed 1996–2019)	0.02	0.02*
A. palmata relative percent production (all 46 sites)	0.02	0.14
A. palmata relative percent production (32 sites surveyed 1996–2019)	0.01	0.11
Orbicella spp. cover (all 46 sites)	-0.02	0.84
Orbicella spp. cover (32 sites surveyed 1996–2019)	-0.01	0.73
Orbicella spp. relative percent production (all 46 sites)	0.01	0.28
Orbicella spp. relative percent production (32 sites surveyed 1996–2019)	<0.01	0.31
S. siderea cover (all 46 sites)	-0.09	1.00
S. siderea cover (32 sites surveyed 1996–2019)	0.04	0.001*
S. siderea relative percent production (all 46 sites)	-0.02	0.86
S. siderea relative percent production (32 sites surveyed 1996–2019)	<-0.01	0.62
P. astreoides cover (all 46 sites)	-0.05	1.00
P. astreoides cover (32 sites surveyed 1996–2019)	0.04	0.002*
P. astreoides relative percent production (all 46 sites)	-0.01	0.67
P. astreoides relative percent production (32 sites surveyed 1996–2019)	-0.01	0.75
M. cavernosa cover (all 46 sites)	-0.05	1.00
M. cavernosa cover (32 sites surveyed 1996–2019)	0.05	0.001*
M. cavernosa relative percent production (all 46 sites)	-0.03	0.95
M. cavernosa relative percent production (32 sites surveyed 1996–2019)	<-0.01	0.66
A. cervicornis cover (all 46 sites)	0.08	0.001*
A. cervicornis cover (32 sites surveyed 1996–2019)	-0.01	0.87
A. cervicornis relative percent production (all 46 sites)	0.01	0.27
A. cervicornis relative percent production (32 sites surveyed 1996–2019)	<-0.01	0.64
C. natans cover (all 46 sites)	-0.06	1.00
C. natans cover (32 sites surveyed 1996–2019)	0.06	0.001*
C. natans relative percent production (all 46 sites)	-0.02	0.88
C. natans relative percent production (32 sites surveyed 1996–2019)	<0.01	0.41
Parrotfish bioerosion (all 46 sites)	0.04	0.009*
Parrotfish bioerosion (32 sites surveyed 1996–2019)	<0.01	0.32
Microbioerosion (all 46 sites)	0.01	0.17
Microbioerosion (32 sites surveyed 1996–2019)	<0.01	0.44
Diadema bioerosion (all 46 sites)	<-0.01	0.53
Diadema bioerosion (32 sites surveyed 1996–2019)	-0.01	0.83
Sponge bioerosion (all 46 sites)	-0.01	0.83
Sponge bioerosion (32 sites surveyed 1996–2019)	-0.01	0.76

Table S6. Mean (±standard error [SE]) coral cover, gross carbonate production, bioerosion, net carbonate production, and reef-accretion potential through time the 49 sites analyzed in this study. Provided as a supplementary file.

Table S7. Comparison of mean bioerosion rates estimated in this study to means from other published carbonate budget studies from south Florida and other locations throughout the western Atlantic (bold values). For locations where mean bioerosion rates are based on more than one site or year of surveys, the range of values among sites and/or years are given in parentheses. Otherwise, uncertainties in parentheses are given as standard errors of transect-level estimates.

	Parrotfishes	***Microbioerosion***	***Sponges***	***Urchins***	***Total***
This study	**0.26**<br>(<0.01 to 1.49)	**0.23**<br>(0.22 to 0.24)	**0.01**<br>(0.00 to 0.03)	**0.01**<br>(0.00 to 1.63)	**0.51**<br>(0.23 to 1.73)
Dry Tortugas<br>(Perry et al., 2018)	**0.22**<br>(± 0.08)	**0.38**<br>(± 0.04)	**0.01**<br>(± <0.01)	**0.01**<br>(± 0.01)	**0.62**<br>(± 0.09)
Cheeca Rocks<br>(Manzello et al., 2018)	**0.78**<br>(0.51 to 1.21)	**0.25**<br>(0.22 to 0.28)	**<0.01**<br>(<0.01 to 0.01)	**0.02**<br>(0.01 to 0.18)	**1.12**<br>(0.87 to 1.56)
Hen & Chickens<br>(Kuffner et al., 2019)	**1.55**<br>(± 0.78)	**0.22**<br>(± 0.01)	**0.13**<br>(± 0.02)	**0.00**<br>(± 0.00)	**1.92**<br>(± 0.47)
Southeast Florida<br>(Perry et al., 2018)	**0.88**<br>(0.44 to 1.68)	**0.26**<br>(0.24 to 0.29)	**0.01**<br>(0.00 to 0.02)	**0.01**<br>(0.00 to 0.02)	**1.17**<br>(0.71 to 2.00)
Western Atlantic<br>(Perry et al., 2018)	**1.54**<br>(0.01 to 6.83)	**0.31**<br>(0.13 to 0.61)	**0.05**<br>(0.00 to 0.24)	**0.10**<br>(0.00 to 1.64)	**1.64**<br>(0.21 to 5.66)

Table S8. Mean (±standard error [SE]) densities (individuals 100 m^{-2}) of bioeroding parrotfish species in the Florida Keys based on the Reef Visual Census data aligned with our study sites.

Species	***Density***
Scarus iseri	5.44 (±0.17)
Sparisoma aurofrenatum	1.91 (±0.06)
Sparisoma viride	0.57 (±0.02)
Sparisoma chrysopterum	0.20 (±0.02)
Scarus taeniopterus	0.19 (±0.02)
Sparisoma rubripinne	0.17 (±0.01)
Scarus coelestinus	0.06 (±0.01)
Scarus vetula	0.05 (±0.01)
Scarus guacamaia	0.06 (±0.01)
**All parrotfishes**	**8.65 (±0.22)**

Supplementary Figures

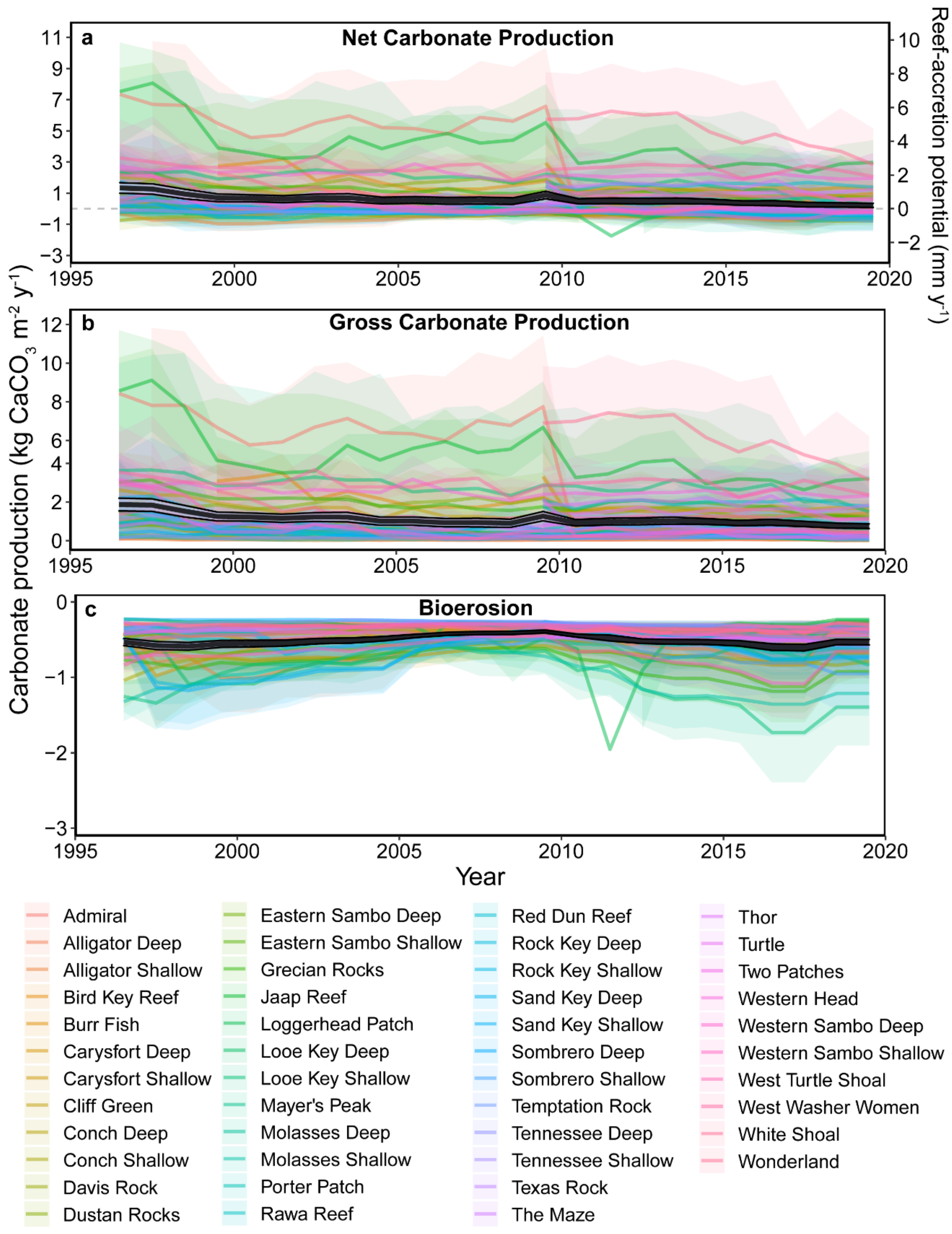

Figure S1. Mean (solid lines) ±1 standard error (SE; shading) a) net carbonate production and reef-accretion potential, b) gross carbonate production, and c) bioerosion over time at each of the 46 CREMP sites analyzed in this study compared with region-wide trends (black line and gray shading).

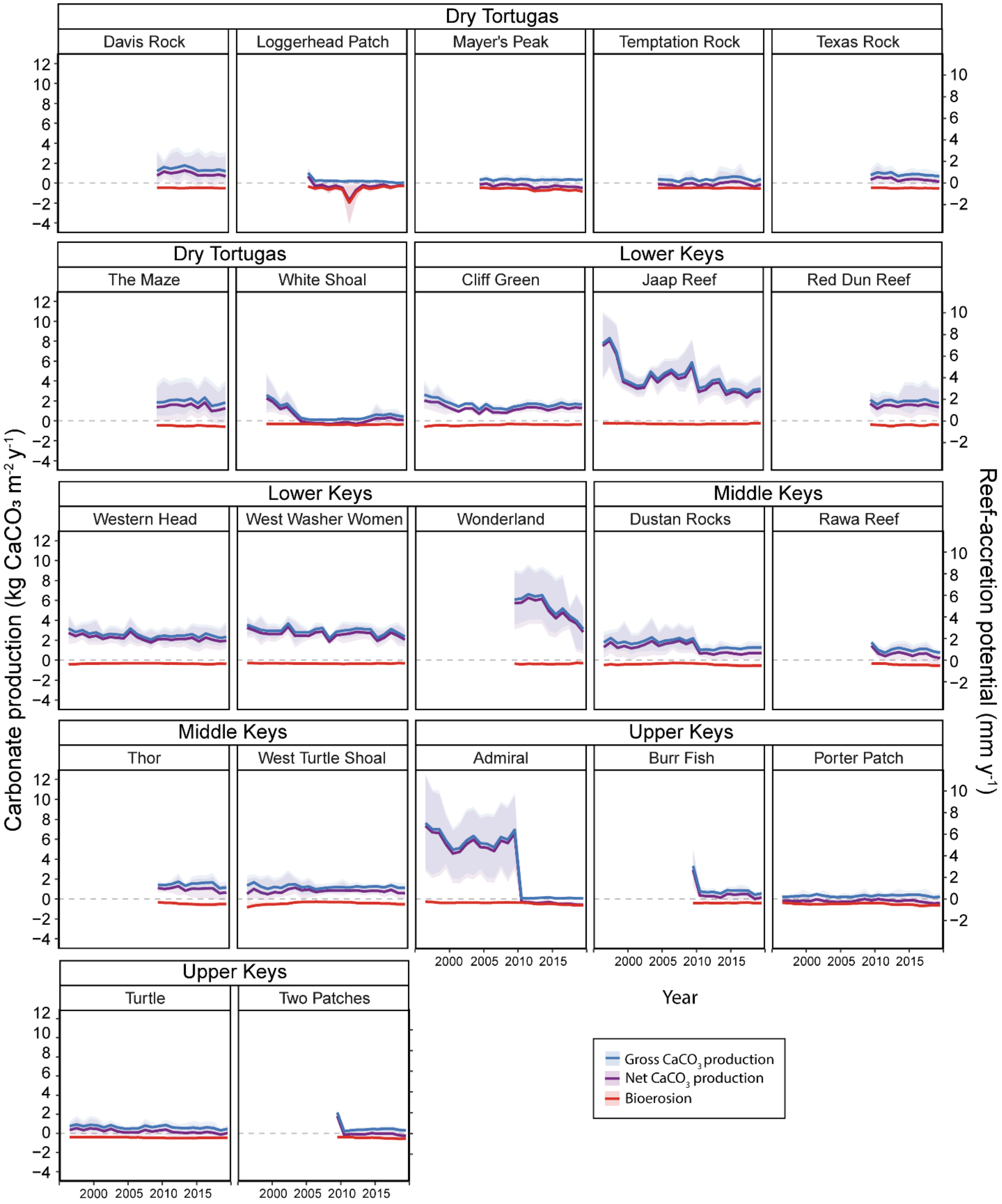

Figure S2. Mean (solid lines) ±1 standard error (SE; shading) net carbonate production and reef-accretion potential, gross carbonate production, and bioerosion for each site in patch-reef habitats across the Florida Keys reef tract arranged by subregion.

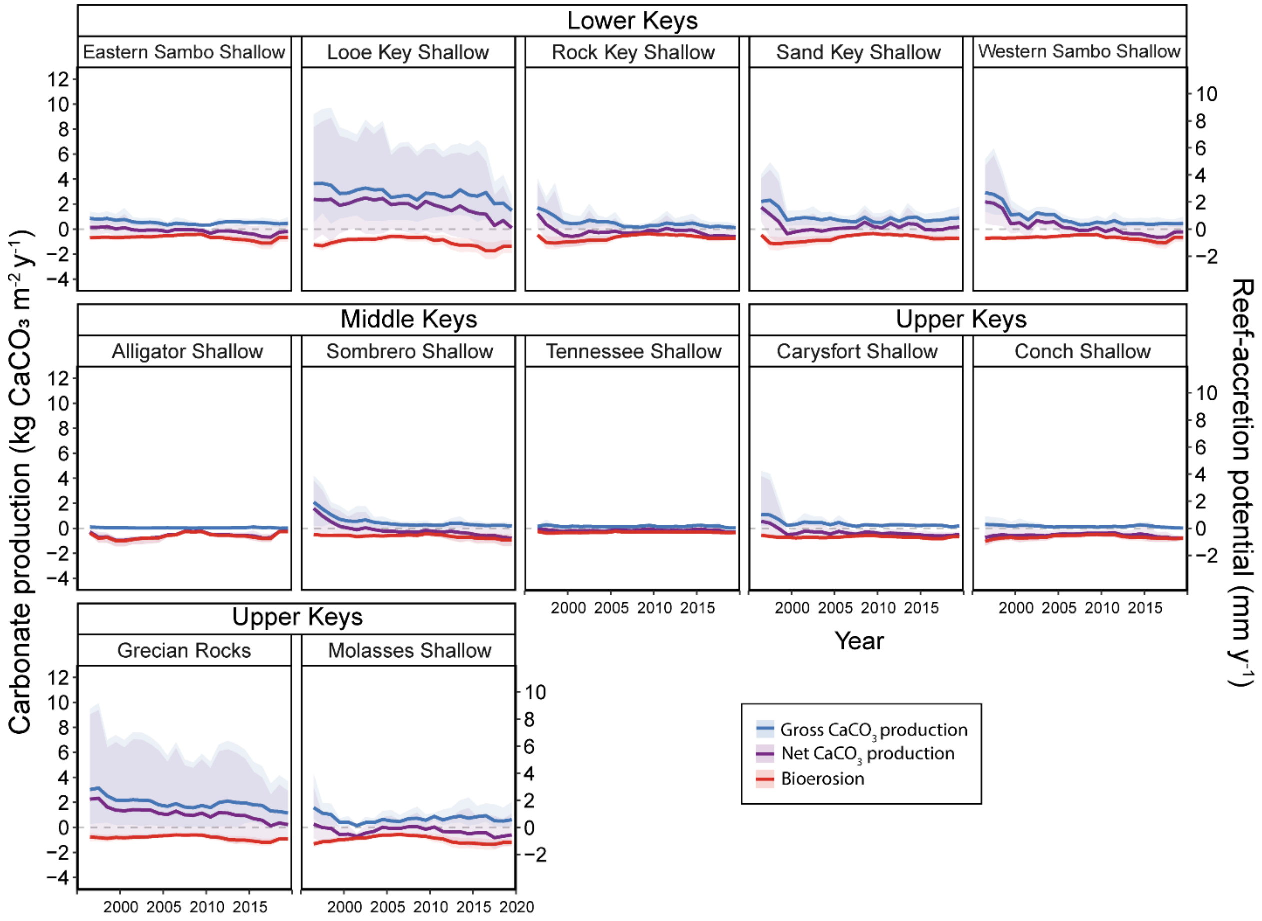

Figure S3. Mean (solid lines) ±1 standard error (SE; shading) net carbonate production and reef-accretion potential, gross carbonate production, and bioerosion for each site in offshore shallow reef habitats across the Florida Keys reef tract arranged by subregion.

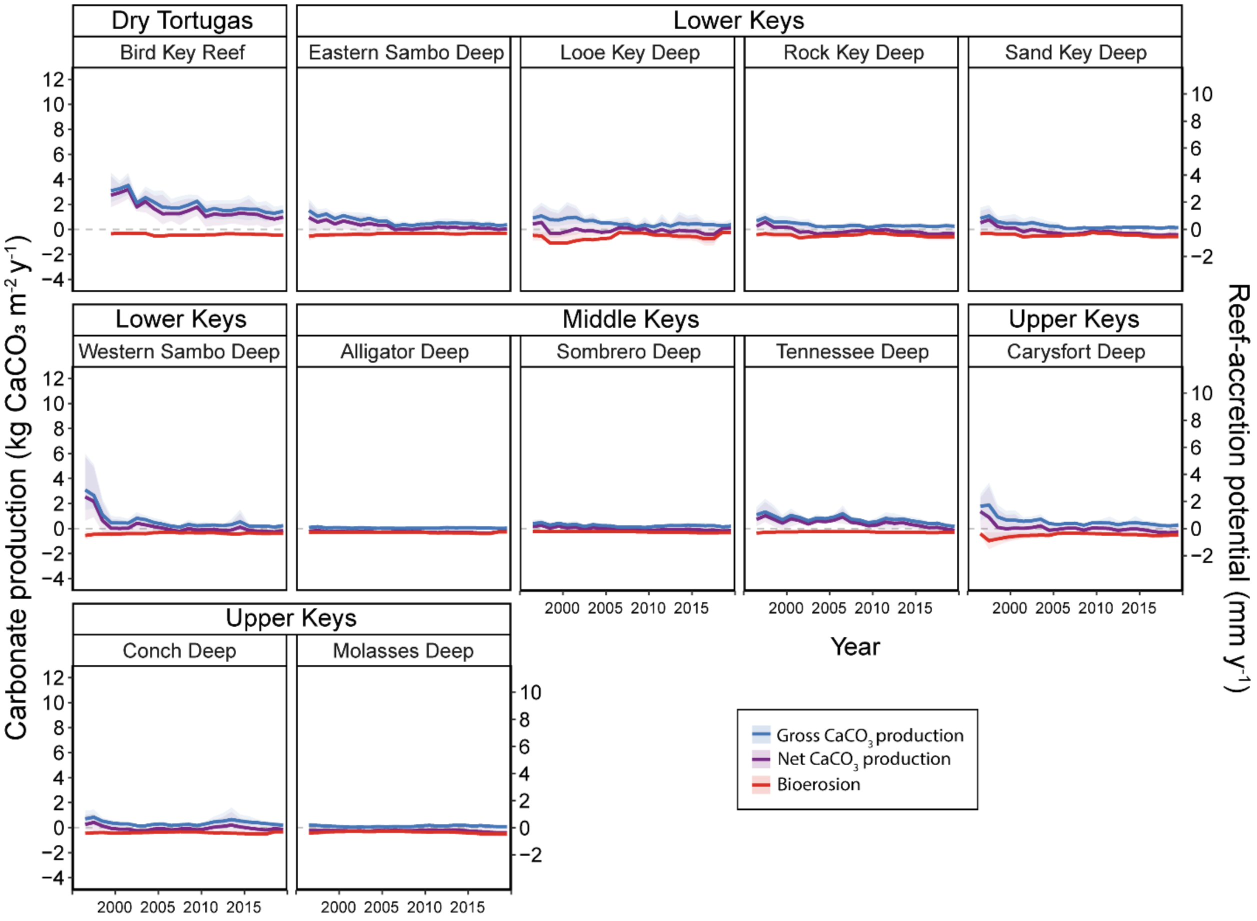

Figure S4. Mean (solid lines) ±1 standard error (SE; shading) net carbonate production and reef-accretion potential, gross carbonate production, and bioerosion for each site in offshore deep reef habitats across the Florida Keys reef tract arranged by subregion.

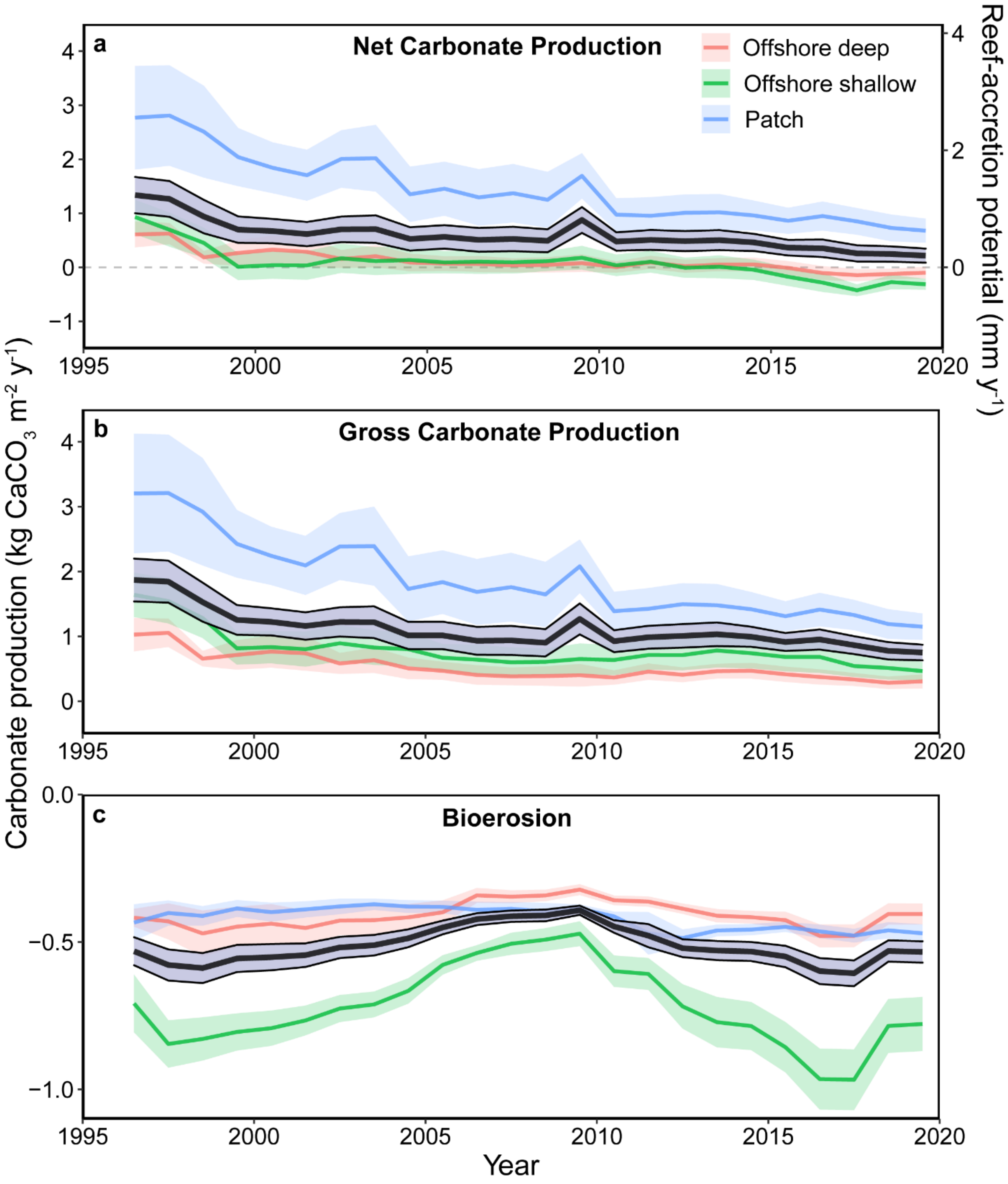

Figure S5. Mean (solid lines) ±1 standard error (SE; shading) a) net carbonate production, b) gross carbonate production, and c) bioerosion over time summarized by habitat compared with region-wide trends (black line and gray shading).

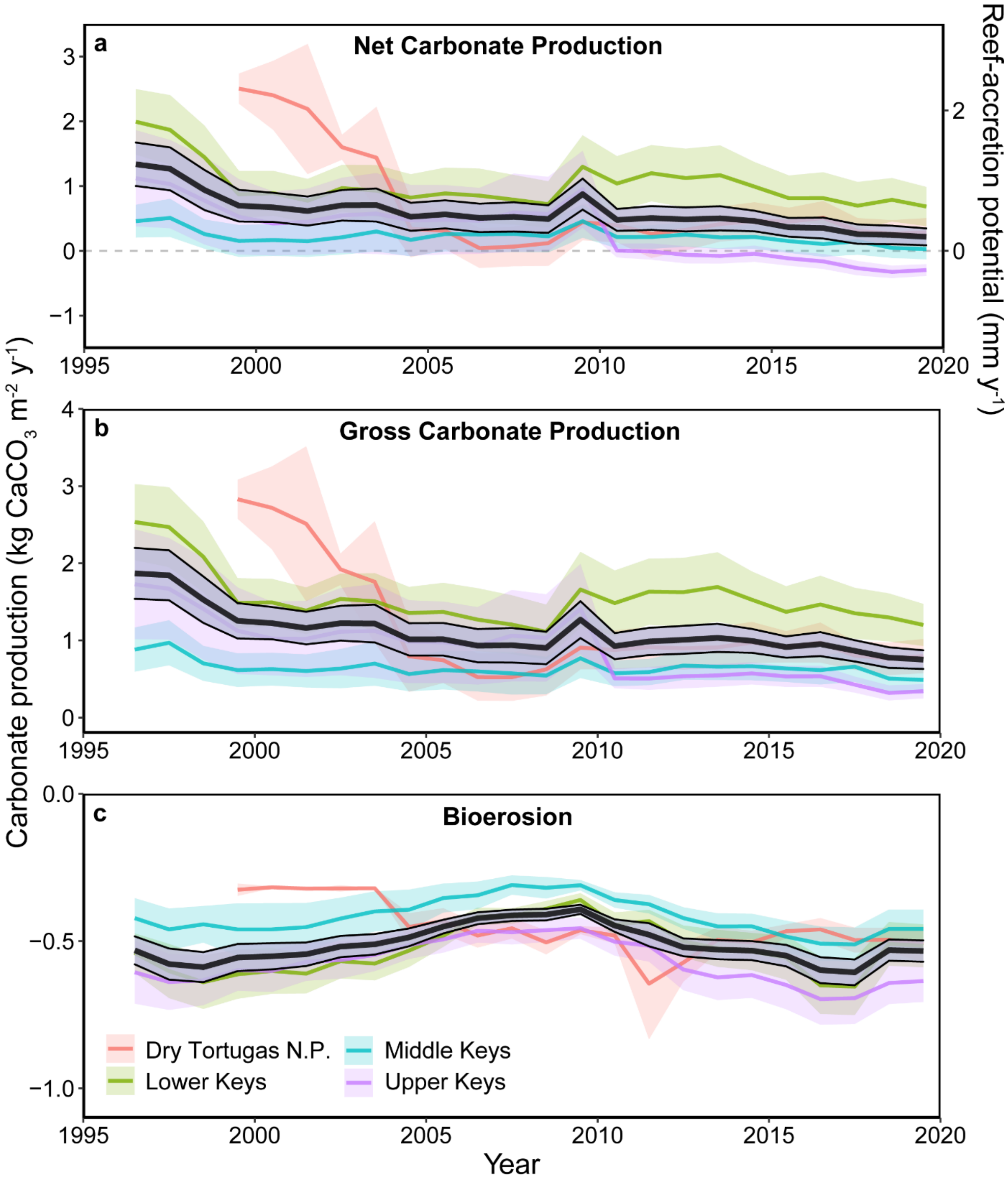

Figure S6. Mean (solid lines) ±1 standard error (SE; shading) a) net carbonate production, b) gross carbonate production, and c) bioerosion over time summarized by subregion compared with region-wide trends (black line and gray shading).

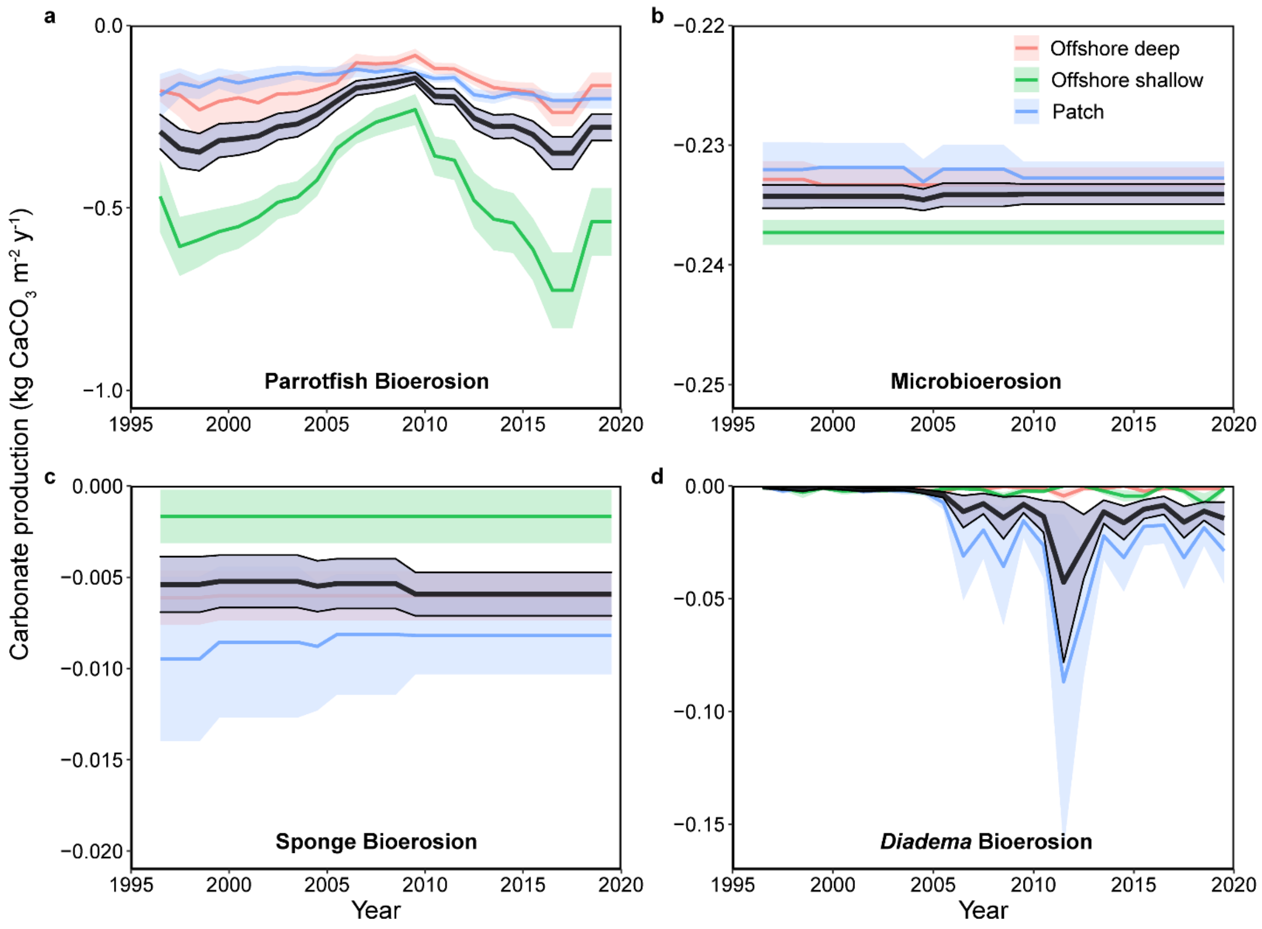

Figure S7. Mean (solid lines) ±1 standard error (SE; shading) bioerosion by a) parrotfishes, b) microbioeroders, c) sponges, and d) *Diadema antillarum* over time summarized by habitat compared with region-wide trends (black line and gray shading).

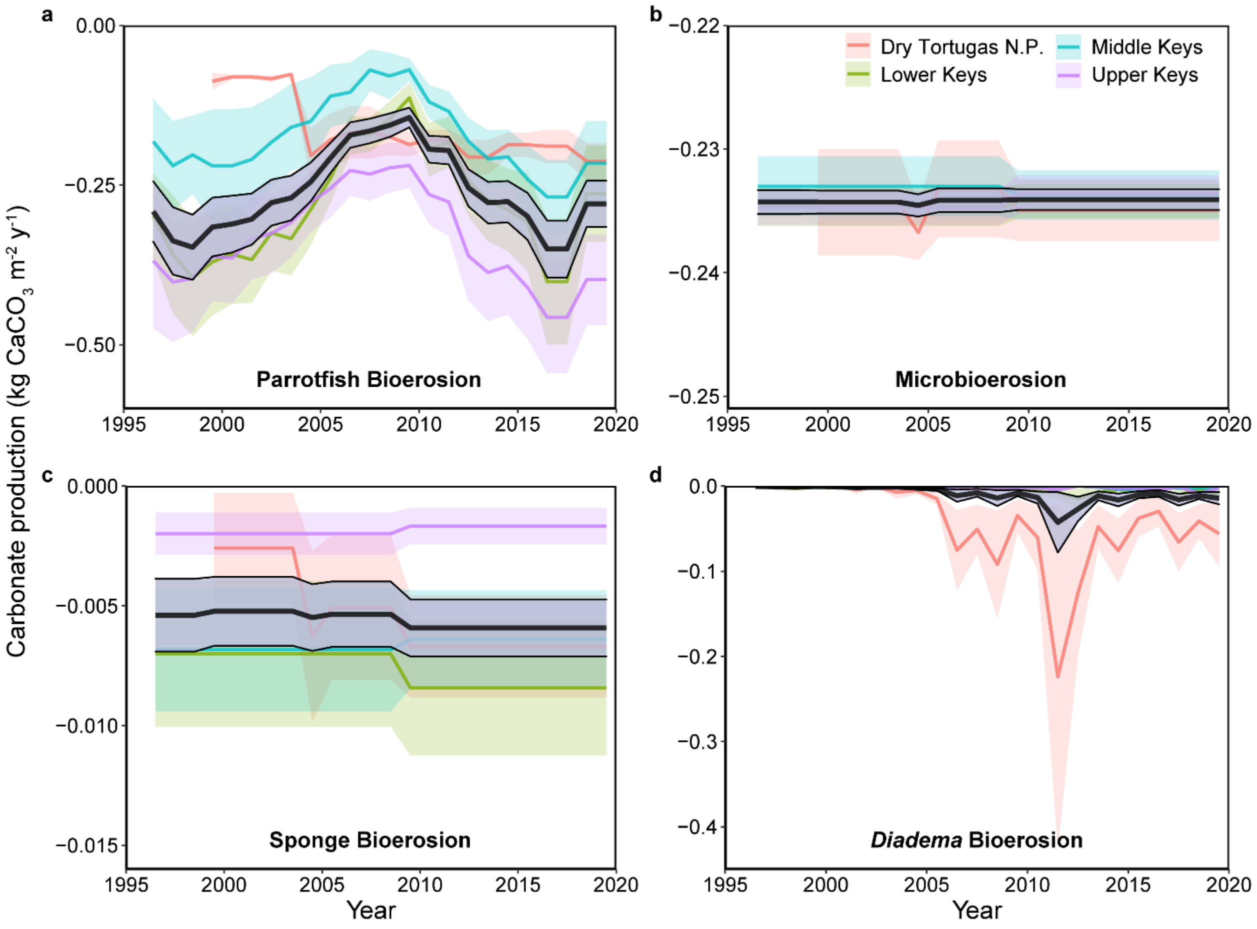

Figure S8. Mean (solid lines) ±1 standard error (SE; shading) bioerosion by a) parrotfishes, b) microbioeroders, c) sponges, and d) *Diadema antillarum* over time summarized by subregion compared with region-wide trends (black line and gray shading).

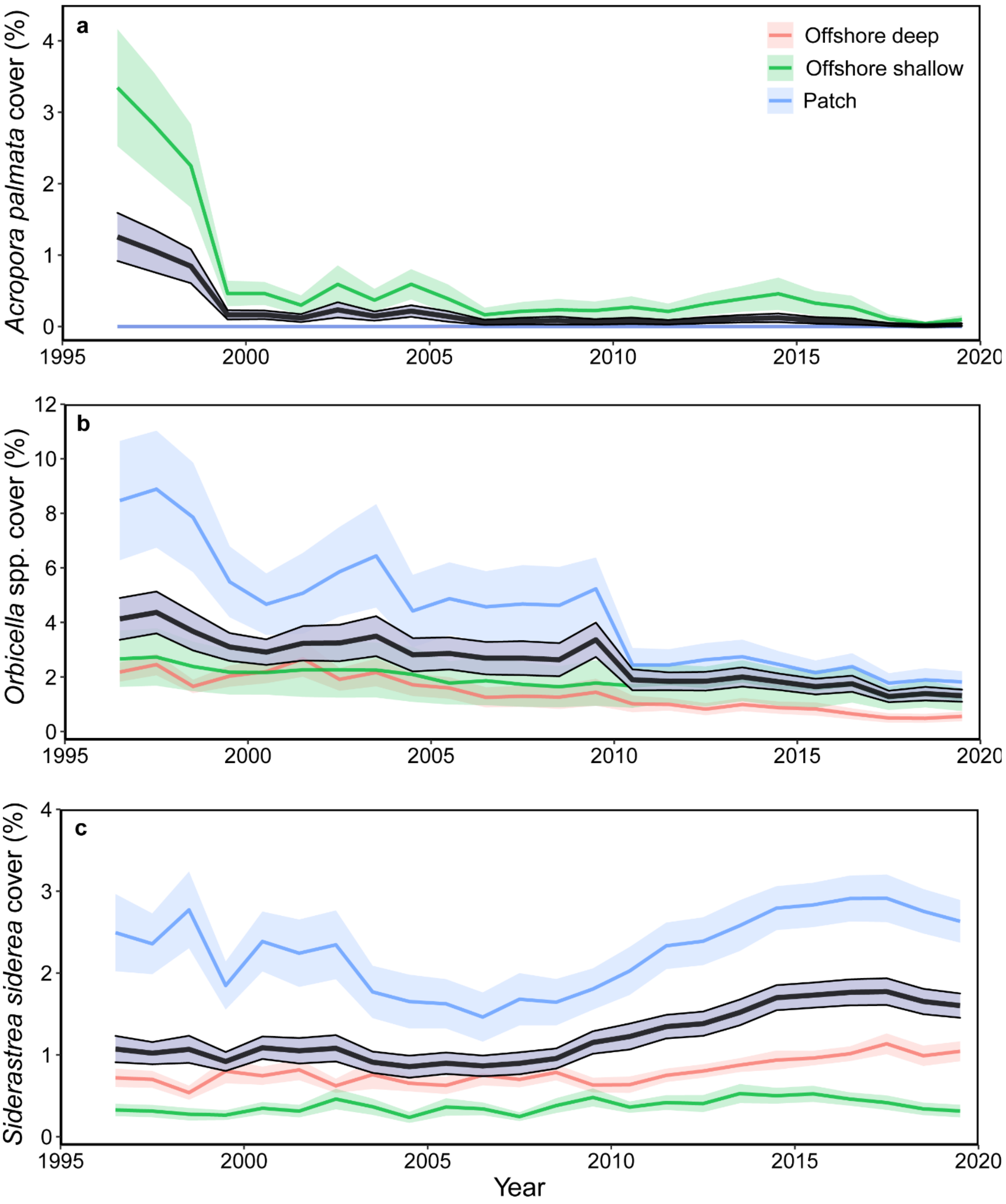

Figure S9. Mean (solid lines) ±1 standard error (SE; shading) percent cover of a) *Acropora palmata*, b) *Orbicella* spp., and c) *Siderastrea siderea* over time summarized by habitat compared with region-wide trends (black line and gray shading).

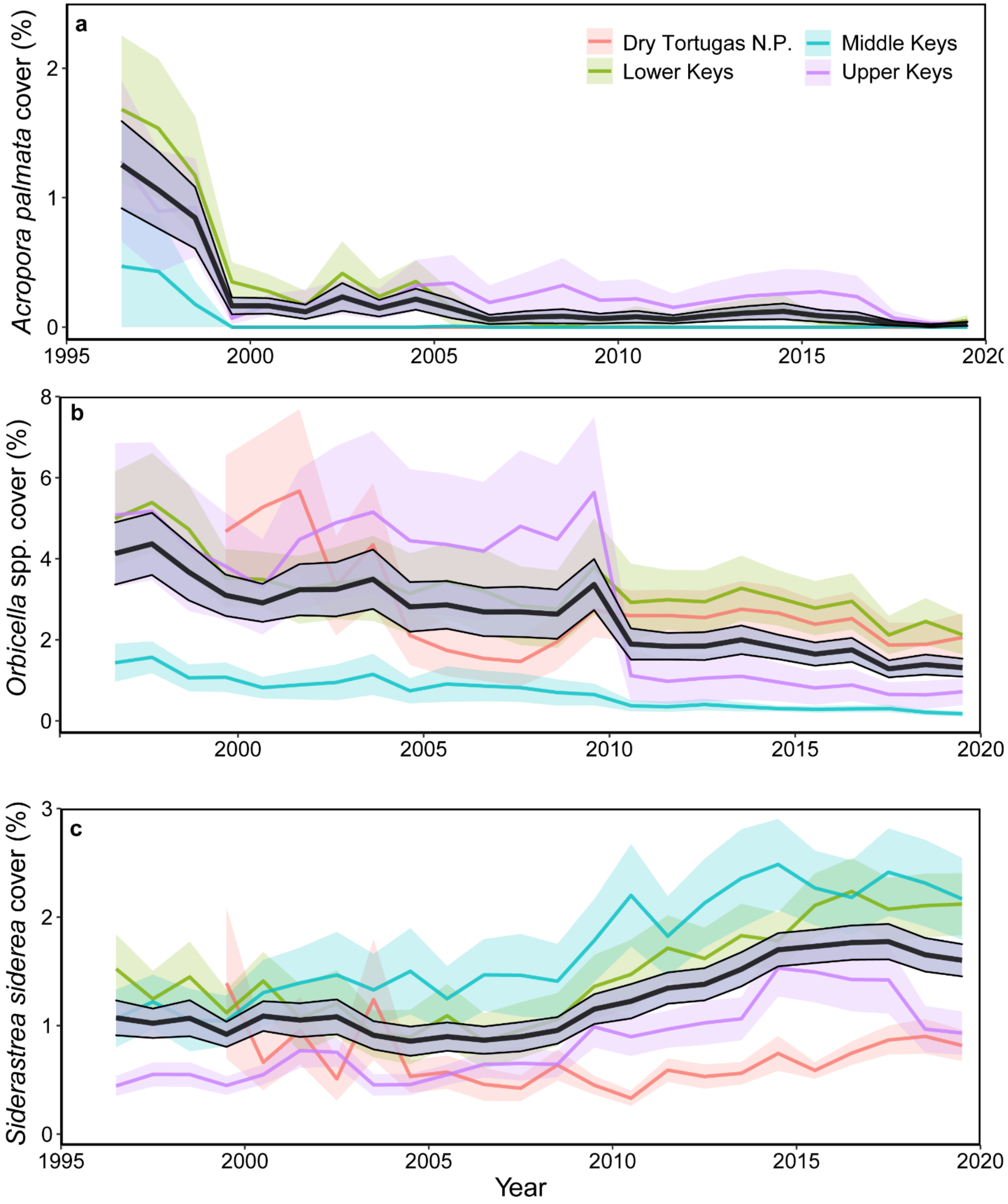

Figure S10. Mean (solid lines) ±1 standard error (SE; shading) percent cover of a) *Acropora palmata*, b) *Orbicella* spp., and c) *Siderastrea siderea* over time summarized by habitat compared with region-wide trends (black line and gray shading).

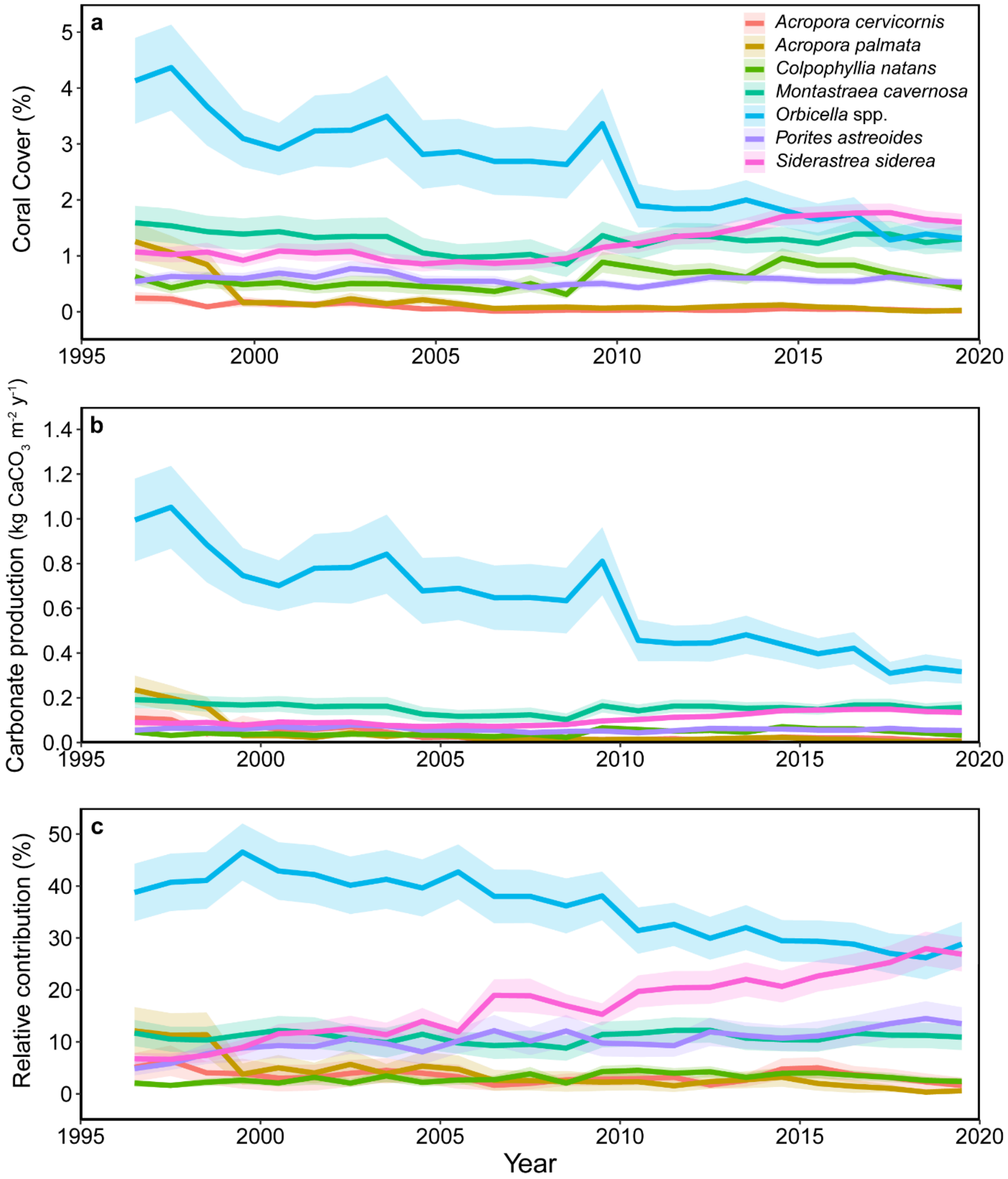

Figure S11. Trends in mean (solid lines) ± standard error (SE; shaded areas) a) percent cover, b) gross carbonate production, and c) relative percent contribution to gross carbonate production of the seven coral taxa that had mean carbonate production rates >0.05 kg m^{-2} y^{-1} during any year of the study.

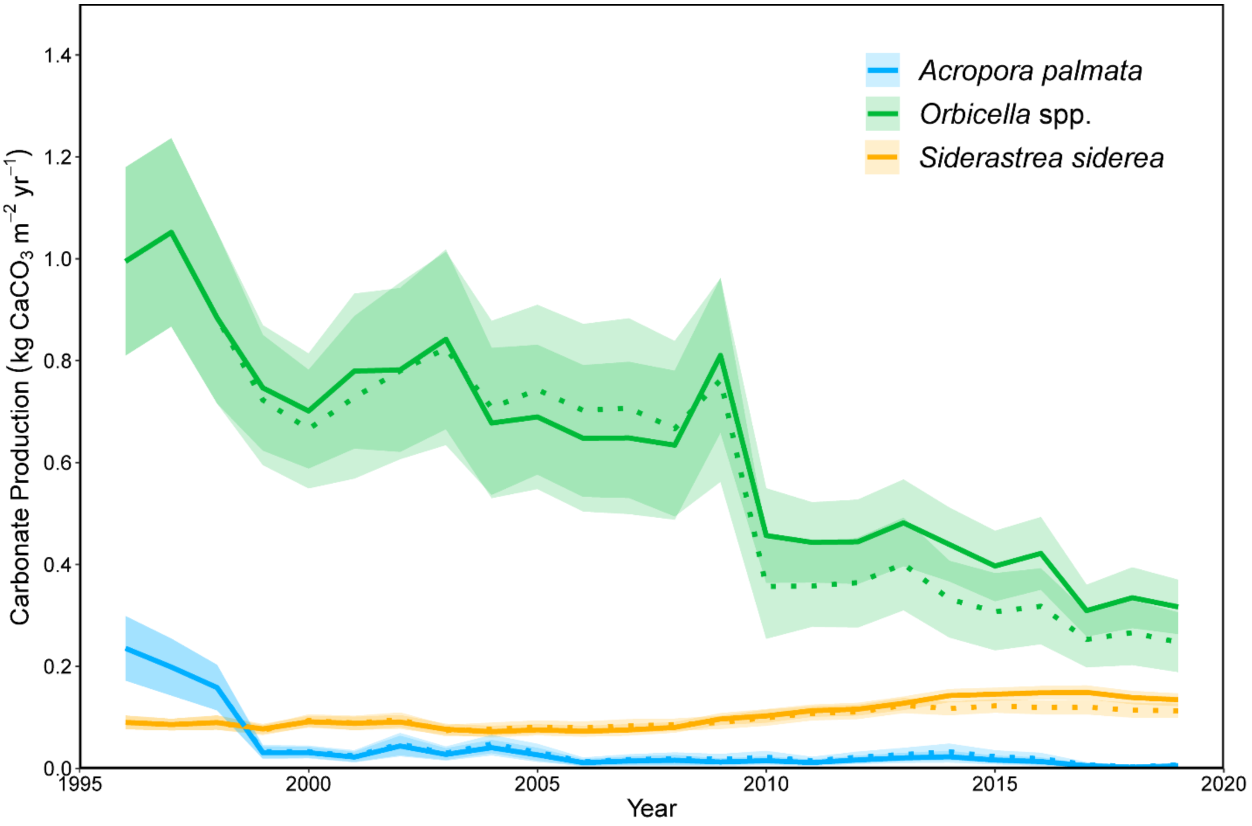

Figure S12. Trends in mean (solid lines) ± standard error (SE; shaded areas) carbonate production by *Acropora palmata*, *Orbicella* spp., and *Siderea siderea*.

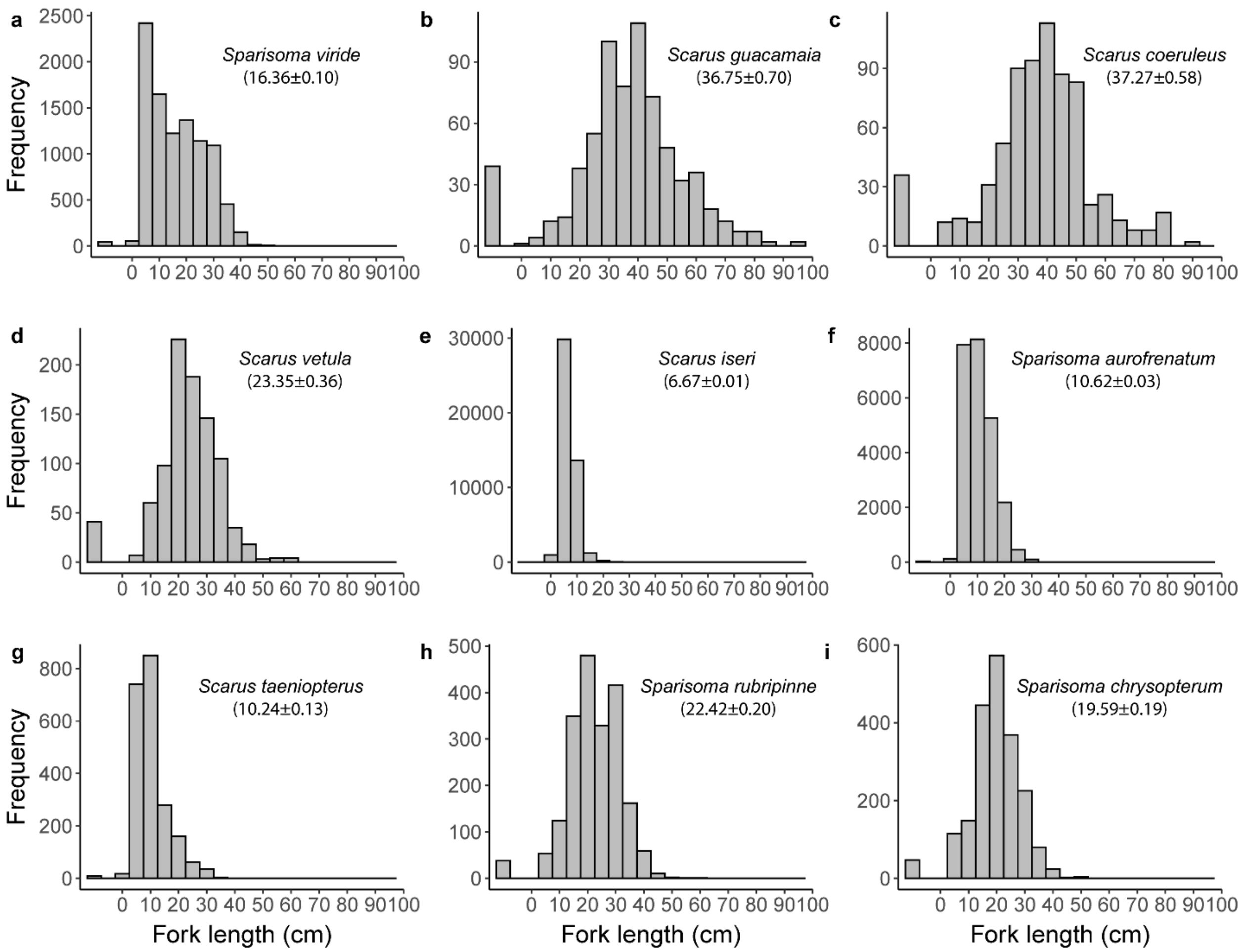

Figure S13. Size-frequency distributions of bioeroding parrotfishes in the Florida Keys based on the Reef Visual Census data aligned with our sites. Species are listed in order of their relative contribution to bioerosion.

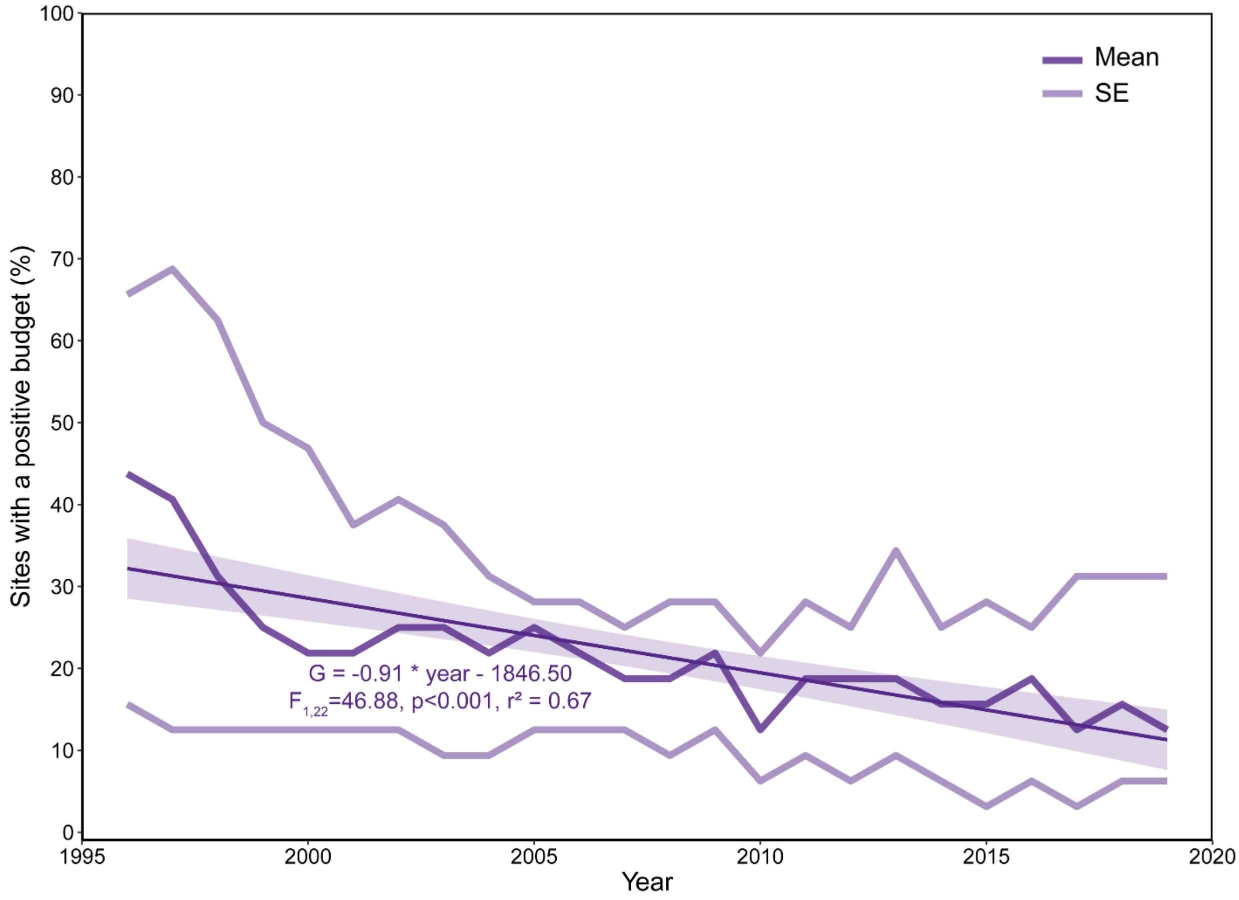

Figure S14. Changes in the mean (dark purple lines) ± standard error (SE; light purple lines) percentage of the 32 sites surveyed from 1996–2019 with net positive carbonate production (G) using the western Atlantic mean bioerosion rate reported in (Perry et al., 2018).

Disclaimer: Any use of trade, firm, or product names is for descriptive purposes only and does not imply endorsement by the U.S. Government.

Supplementary References

Adam, T. C., Kelley, M., Ruttenberg, B. I., & Burkepile, D. E. (2015). Resource partitioning along multiple niche axes drives functional diversity in parrotfishes on Caribbean coral reefs. *Oecologia, 179*(4), 1173-1185. doi:10.1007/s00442-015-3406-3

Bohnsack, J. A., & Bannerot, S. P. (1986). A stationary visual census technique for quantitatively assessing community structure of coral reef fishes. https://repository.library.noaa.gov/view/noaa/1005.

Brandt, M. E., Zurcher, N., Acosta, A., Ault, J. S., Bohnsack, J. S., Feeley, M. W., . . . Smith, S. G. (2009). A Cooperative Multi-agency Reef Fish Monitoring Protocol for the Florida Keys Coral Reef Ecosystem. *Natural Resource Report NPS/SFCN/NRR—2009/150* https://www.coris.noaa.gov/activities/fish_monitoring_protocol/.

Burkepile, D. E. (2012). Context-dependent corallivory by parrotfishes in a Caribbean reef ecosystem. *Coral Reefs, 31*(1), 111-120.

Callahan, M. K. (2005). *Distribution of Clionid Sponges in the Florida Keys National Marine Sanctuary (FKNMS), 2001-2003.* (M.S.), University of South Florida, St. Petersburg, FL.

Chazottes, V., Campion-Alsumard, T. L., & Peyrot-Clausade, M. (1995). Bioerosion rates on coral reefs: interactions between macroborers, microborers and grazers (Moorea, French Polynesia). *Palaeogeography, Palaeoclimatology, Palaeoecology, 113*(2), 189-198. https://doi.org/10.1016/0031-0182(95)00043-L.

Chazottes, V., Le Campion-Alsumard, T., Peyrot-Clausade, M., & Cuet, P. (2002). The effects of eutrophication-related alterations to coral reef communities on agents and rates of bioerosion (Reunion Island, Indian Ocean). *Coral Reefs, 21*(4), 375-390.

Chiappone, M., Rutten, L. M., Swanson, D. W., & Miller, S. L. (2008). *Population status of the urchin Diadema antillarum in the Florida Keys 25 years after the Caribbean mass mortality.* Paper presented at the Proceedings of the 11th International Coral Reef Symposium, Ft. Lauderdale.

Courtney, T. A., Chan, S., Lange, I. D., Perry, C. T., Kriegman, D. J., & Andersson, A. J. (2021). Area-normalized scaling of ReefBudget calcification, macrobioerosion, and microbioerosion rates for use with CoralNet Version 1.0. https://doi.org/10.5281/zenodo.5140477.

de Bakker, D. M., Webb, A. E., van den Bogaart, L. A., van Heuven, S. M. A. C., Meesters, E. H., & van Duyl, F. C. (2018). Quantification of chemical and mechanical bioerosion rates of six Caribbean excavating sponge species found on the coral reefs of Curaçao. *PLoS One, 13*(5), e0197824. doi:10.1371/journal.pone.0197824

Enochs, I. C., Manzello, D. P., Carlton, R. D., Graham, D. M., Ruzicka, R., & Colella, M. A. (2015). Ocean acidification enhances the bioerosion of a common coral reef sponge: implications for the persistence of the Florida Reef Tract. *Bulletin of Marine Science, 91*(2), 271–290.

Gintert, B. E., Precht, W. F., Fura, R., Rogers, K., Rice, M., Precht, L. L., . . . Robbart, M. L. (2019). Regional coral disease outbreak overwhelms impacts from a local dredge project. *Environmental Monitoring and Assessment, 191*(10), 630. doi:10.1007/s10661-019-7767-7

Grove, L. J. W., Blondeau, J., & Ault, J. S. (2021). *National Coral Reef Monitoring Program's Reef fish Visual Census Metadata for the U.S. Caribbean*. Retrieved from https://www.fisheries.noaa.gov/inport/item/65840.

Hubbard, D. K., Miller, A. I., & Scaturo, D. (1990). Production and cycling of calcium carbonate in a shelf-edge reef system (St. Croix, U.S. Virgin Islands): applications to the nature of reef systems in the fossil record. *Journal of Sedimentary Petrology, 60*(3), 335–360.

Jokiel, P. L., Rodgers, K. S., Kuffner, I. B., Andersson, A. J., Cox, E. F., & Mackenzie, F. T. (1978). Coral growth: buoyant weight technique. In D. R. Stoddart & R. E. Johannes (Eds.), *Coral reefs: research methods* (pp. 529–541). Paris: UNESCO.

Kinsey, D. W. (1985). Metabolism, calcification and carbon production I: system level studies. *Proceedings of the Fifth International Coral Reef Congress, Tahiti, 4*, 505–526.

Kuffner, I. B., Hickey, T. D., & Morrison, J. M. (2013). Calcification rates of the massive coral *Siderastrea siderea* and crustose coralline algae among the Florida Keys (USA) outer-reef tract. *Coral Reefs, 32*, 987–997.

Kuffner, I. B., Toth, L. T., Hudson, J. H., Goodwin, W. B., Stathakopoulos, A., Bartlett, L. A., & Whitcher, E. M. (2019). Improving estimates of coral reef construction and erosion with in situ measurements. *Limnology and Oceanography, 64*(5), 2283-2294. https://doi.org/10.1002/lno.11184.

Lenz, E. A., Bartlett, L. A., Stathakopoulos, A., & Kuffner, I. B. (2021). Physiological Differences in Bleaching Response of the Coral Porites astreoides Along the Florida Keys Reef Tract During High-Temperature Stress. *Frontiers in Marine Science, 8*(660). doi:10.3389/fmars.2021.615795

Manzello, D. P., Enochs, I. C., Kolodziej, G., & Carlton, R. (2015). Recent decade of growth and calcification of *Orbicella faveolata* in the Florida Keys: an inshore-offshore comparison. *Marine Ecology Progress Series, 521*, 81–89.

Manzello, D. P., Enochs, I. C., Kolodziej, G., Carlton, R., & Valentino, L. (2018). Resilience in carbonate production despite three coral bleaching events in 5 years on an inshore patch reef in the Florida Keys. *Marine Biology, 165*(6), 99. doi:10.1007/s00227-018-3354-7

Molina-Hernández, A., González-Barrios, F. J., Perry, C. T., & Álvarez-Filip, L. (2020). Two decades of carbonate budget change on shifted coral reef assemblages: are these reefs being locked into low net budget states? *Proceedings of the Royal Society B, 287*, 20202305.

Muller, E. M., Sartor, C., Alcaraz, N. I., & van Woesik, R. (2020). Spatial Epidemiology of the Stony-Coral-Tissue-Loss Disease in Florida. *Frontiers in Marine Science, 7*(163). doi:10.3389/fmars.2020.00163

NOAA. (2021). Restoring Seven Iconic Reefs: A Mission to Recover the Coral Reefs of the Florida Keys. https://www.fisheries.noaa.gov/southeast/habitat-conservation/restoring-seven-iconic-reefs-mission-recover-coral-reefs-florida-keys.

Pante, E., & Dustan, P. (2012). Getting to the Point: Accuracy of Point Count in Monitoring Ecosystem Change. *Journal of Marine Biology, 2012*, 802875. doi:10.1155/2012/802875

Perry, C. T., Alvarez-Filip, L., Graham, N. A. J., Mumby, P. J., Wilson, S. K., Kench, P. S., . . . Macdonald, C. (2018). Loss of coral reef growth capacity to track future increases in sea level. *Nature, 558*(7710), 396-400. doi:10.1038/s41586-018-0194-z

Perry, C. T., & Lange, I. D. (2019). ReefBudget: Methodology Caribbean Version 2. https://geography.exeter.ac.uk/reefbudget/.

Perry, C. T., Murphy, G., Edinger, E., Kench, P., Mumby, P., Smithers, S., & Steneck, R. (2012). ReefBudget: Methodolgy. http://geography.exeter.ac.uk/reefbudget/.

Perry, C. T., Murphy, G. N., Kench, P. S., Edinger, E. N., Smithers, S. G., Steneck, R. S., & Mumby, P. J. (2014). Changing dynamics of Caribbean reef carbonate budgets: emergence of reef bioeroders as critical controls on present and future reef growth potential. *Proceedings of the Royal Society of London B, 281*.

Perry, C. T., Murphy, G. N., Kench, P. S., Smithers, S. G., Edinger, E. N., Steneck, R. S., & Mumby, P. J. (2013). Caribbean-wide decline in carbonate production threatens coral reef growth. *Nature Communications, 4*. https://doi.org/10.1038/ncomms2409.

Porter, J. (2001). Detection of coral reef change by the Florida Keys coral reef monitoring project. In *The everglades, Florida Bay, and coral reefs of the Florida Keys* (pp. 773-794): CRC Press.

Rohatgi, A. (2021). WebPlotDigitizer v. 4.5. https://automeris.io/WebPlotDigitizer. Retrieved from https://automeris.io/WebPlotDigitizer.

Rose, C. S., & Risk, M. J. (1985). Increase in Cliona delitrix Infestation of Montastrea cavernosa Heads on an Organically Polluted Portion of the Grand Cayman Fringing Reef. *Marine Ecology, 6*(4), 345-363. doi:https://doi.org/10.1111/j.1439-0485.1985.tb00142.x

Ruzicka, R. R., Semon, K., Colella, M., Brinkhuis, V., Kidney, J., Morrison, J., . . . Colee, J. (2009). *Coral Reef Evaulation and Monitoring Project: 2009 Annual Report*. Retrieved from https://ocean.floridamarine.org/fknms_wqpp/docs/cremp/reports/2009CREMP_Annual_Report.pdf

Sale, P. F. (1997). *Visual census of fishes: how well do we see what is there.* Paper presented at the Proceedings of the 8th International Coral Reef Symposium.

Scoffin, T. P., Stearn, C. W., Boucher, D., Frydl, P., Hawkins, C. M., Hunter, I. G., & MacGeachy, J. K. (1980). Calcium carbonate budget of a fringing reef on the west coast of Barbados. Part II-Erosion, sediments and internal structure. *Bulletin of Marine Science, 30*, 475–508.

Shantz, A. A., Ladd, M. C., & Burkepile, D. E. (2020). Overfishing and the ecological impacts of extirpating large parrotfish from Caribbean coral reefs. *Ecological Monographs, 90*(2), e01403. https://doi.org/10.1002/ecm.1403.

Smith, S. G., Ault, J. S., Bohnsack, J. A., Harper, D. E., Luo, J., & McClellan, D. B. (2011). Multispecies survey design for assessing reef-fish stocks, spatially explicit management performance, and ecosystem condition. *Fisheries Research, 109*(1), 25-41. https://doi.org/10.1016/j.fishres.2011.01.012.

Toth, L.T. and Courtney, T.A. (2022). Keys carbonate budget calculations and analysis. *U.S. Geological Survey software release*, https://doi.org/10.5066/P9APPZHJ.

Toth, L. T., Kuffner, I. B., & Stathakopoulos, A. (2018). Descriptive Core Logs, Core Photographs, Radiocarbon Ages, and Data on Reef Development for Cores of Holocene Reef Framework from the Florida Keys Reef Tract. *U.S. Geological Survey data release*. https://doi.org/10.5066/F7NV9HJX.

Toth, L. T., Kuffner, I. B., Stathakopoulos, A., & Shinn, E. A. (2018). A 3,000-year lag between the geological and ecological shutdown of Florida's coral reefs. *Global Change Biology, 24*(11), 5471-5483. https://doi.org/10.1111/gcb.14389.

Tribollet, A., & Golubic, S. (2005). Cross-shelf differences in the pattern and pace of bioerosion of experimental carbonate substrates exposed for 3 years on the northern Great Barrier Reef, Australia. *Coral Reefs, 24*(3), 422-434. doi:10.1007/s00338-005-0003-7

Ward-Paige, C., Mills Flemming, J., & Lotze, H. K. (2010). Overestimating Fish Counts by Non-Instantaneous Visual Censuses: Consequences for Population and Community Descriptions. *PLoS One, 5*(7), e11722. doi:10.1371/journal.pone.0011722

Wheaton, J., Jaap, W. C., Porter, J. W., Kosminyn, V., Hackett, K., Lybolt, M., . . . Tsokos, C. (2001). *EPA/FKNMS Coral Reef Monitoring Project.* Paper presented at the FKNMS symposium: an ecosystem report card, Washington DC. https://ocean.floridamarine.org/fknms_wqpp/docs/cremp/reports/2001CREMPExecutiveSummary.pdf.
